# Supplementary material for: A gut microbiota rheostat forecasts responsiveness to PD-L1 and VEGF blockade in mesothelioma
Source: Nat Commun. 2024 Aug 21;15:7187. doi: 10.1038/s41467-024-49842-5 (PMC11339264; doi:10.1038/s41467-024-49842-5)
Supplement: Supplementary file 1 — Supplementary Information [file 41467_2024_49842_MOESM1_ESM.pdf]

A Gut Microbiota Rheostat Forecasts Responsiveness to PD-L1 and VEGF Blockade in Mesothelioma

Min Zhang, Aleksandra Bzura, Jan Rogel, Zisen Zhou, Essa Y. Baitei, Jake Spicer, Charlotte Poile, Amy Branson, Amy King, Shaun Barber, Tamihiro Kamata, Joanna Dzialo, James Harber, Alastair Greystoke, Nada Nusrat, Daniel Faulkner, Qianqian Sun, Luke Nolan, Jens C. Hahne, Molly Scotland, Harriet Walter, Liz Darlison, Bruno Morgan, Amrita Bajaj, Cassandra Brookes, Edward J. Hollox, Dominika Lubawska, Maymun Jama, Gareth Griffiths, Apostolos Nakas, Kudzayi Kutywayo, Jin-Li Luo, Astero Klampatsa, Andrea Cooper, Koirobi Halder, Peter Wells-Jordan, Huiyu Zhou, Frank Dudbridge, Anne Thomas, Catherine Jane Richards, Catrin Pritchard, Hongji Yang, Michael Barer, Dean A. Fennell

SUPPLEMENTARY INFORMATION

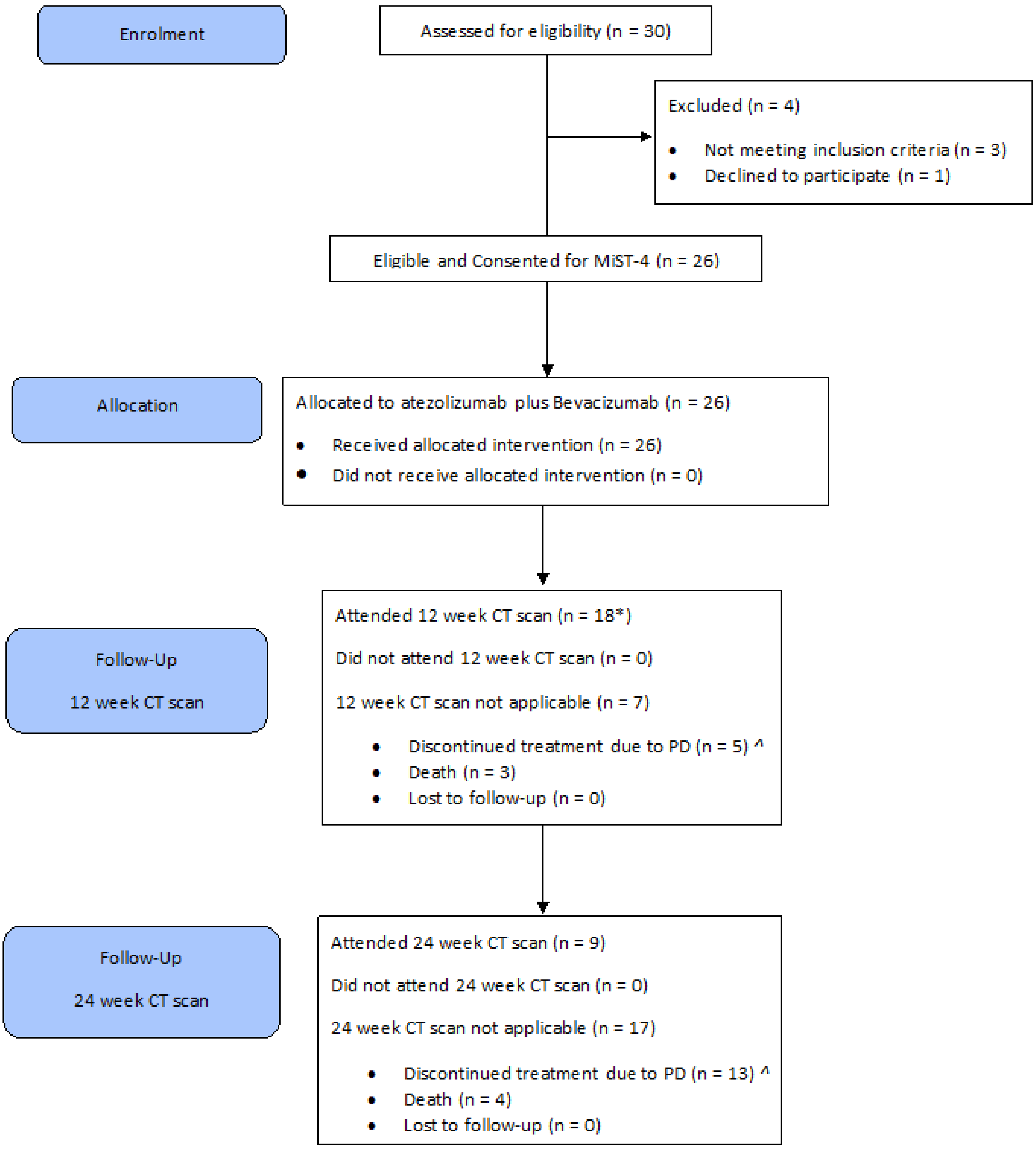

Supplementary Figure 1
Consort diagram for MIST4.
*Two of the 12 week CT scans were carried out earlier than the time window (around 10.7 weeks).
^includes clinical disease progression

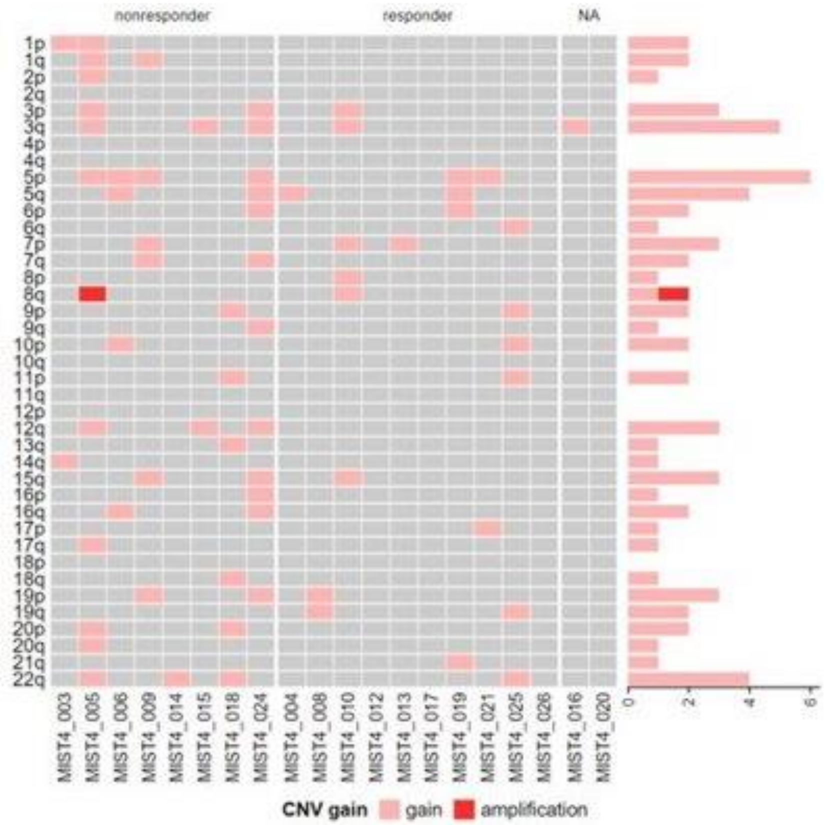

Supplementary figure 2.
Copy number gains correlate with response to PDL1-VEGF blockade.
Heatmap showing the relative burden of copy gain (light red)/amplification (dark red) in NR versus R subgroup.

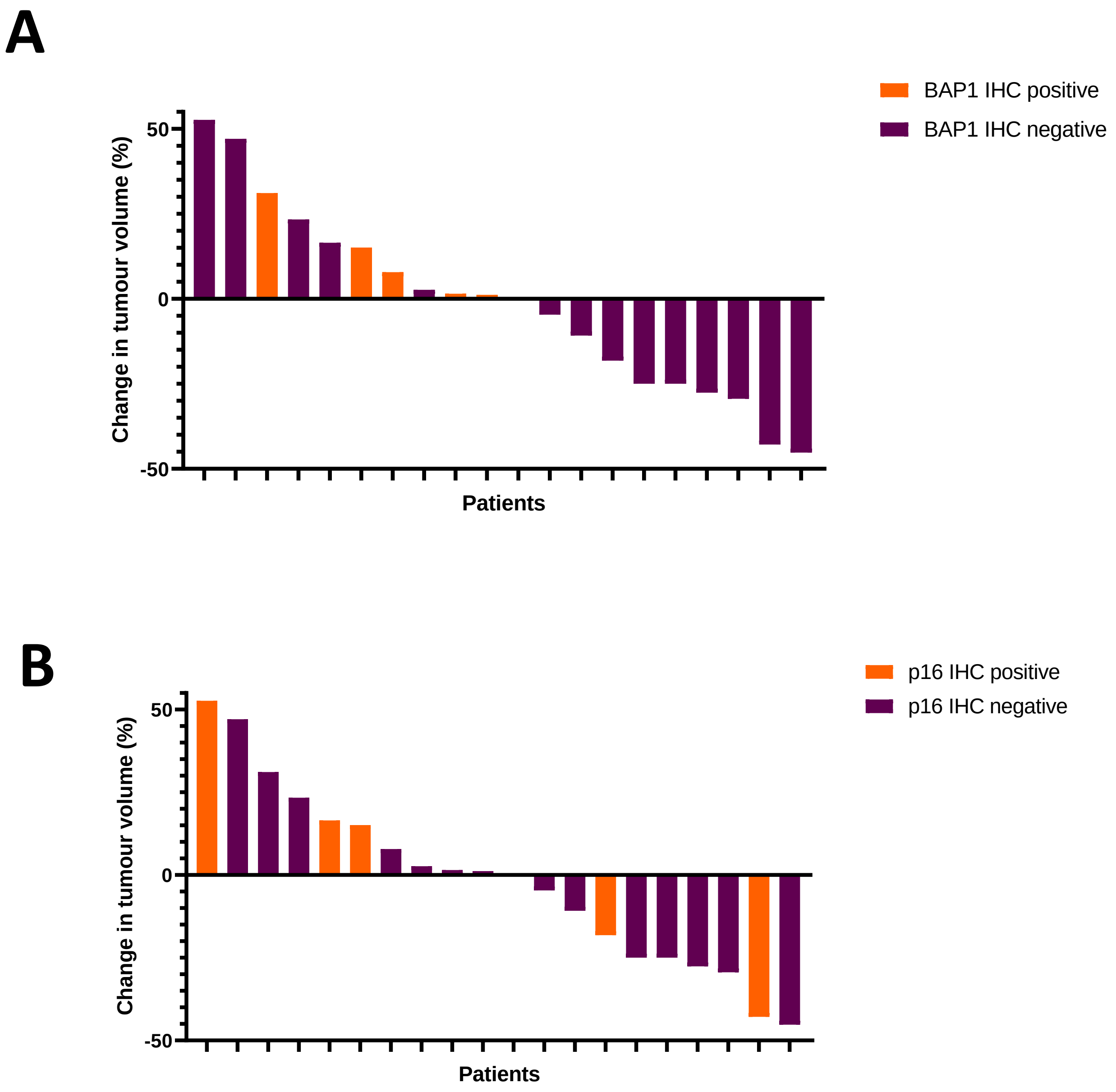

Supplementary figure 3.
BAP1 and p16ink4a expression and response in MIST4
A. A waterfall plot showing the prevalence of BAP1 deficient mesothelioma (complete loss of expression or loss of nuclear expression in orange; positive expression in blue). Right panel. BAP1 expression was not significantly associated with response (change in tumour volume). B. A waterfall plot showing the prevalence of p16ink4a (p16) deficient mesothelioma. Right panel. P16ink4a expression was not associated with response in patients.

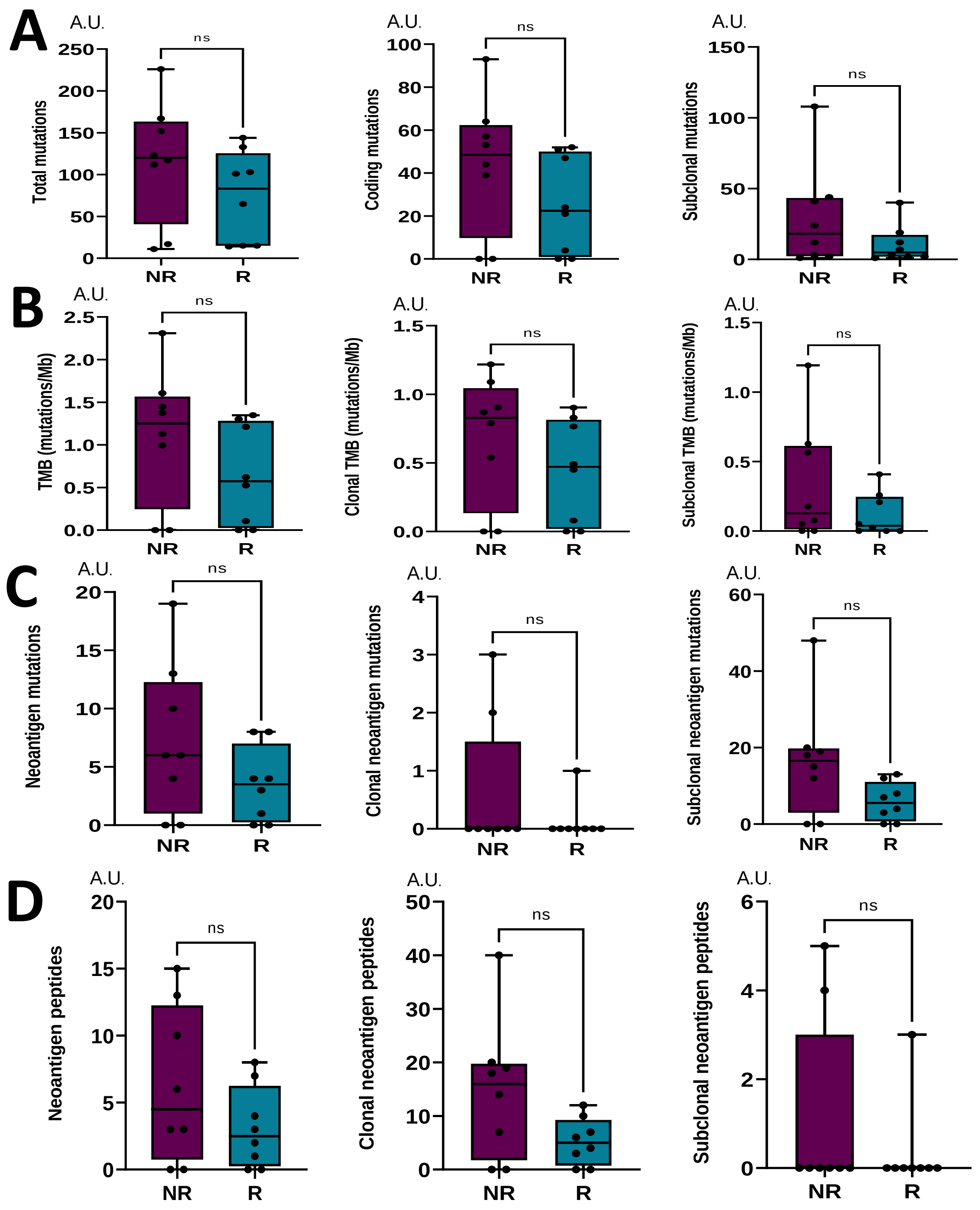

Supplementary figure 4.
Neoantigen burden does not correlate with response to PDL1-VEGF blockade
A. Box plots showing the relative burden of total (left), clonal (middle) and subclonal (right) mutations in the NR versus R subgroups
B. Box plots showing the relative burden of total (left), clonal (middle) and subclonal (right) tumour mutation burden (TMB) in the NR versus R subgroups
C. Box plots showing the relative burden of total (left), clonal (middle) and subclonal (right) predicted neoantigen mutations in the NR versus R subgroups
D. Box plots showing the relative burden of total (left), clonal (middle) and subclonal (right) predicted neoantigen peptides in the NR versus R subgroups

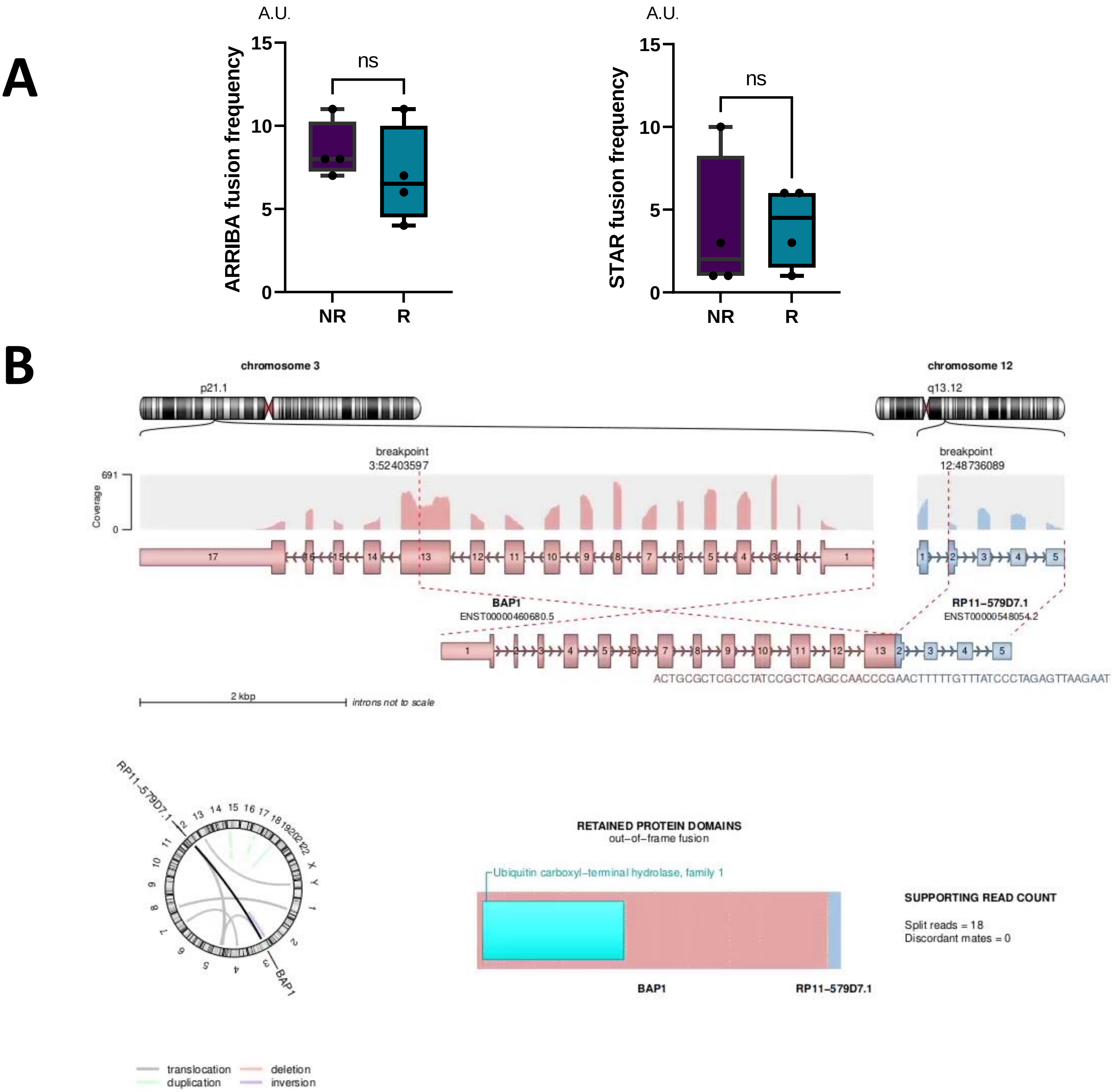

Supplementary figure 5.

Gene rearrangements in MIST4 do not correlate with response

A. Box plots showing the relative frequency of fusion events in NR (purple) versus R subgroups (blue)

B. Identification of putative inactivating BAP1-RP11-579D7.1 fusion illustrating the breakpoint involving translocation involving chromosome 3p21.1 and 12q13.12

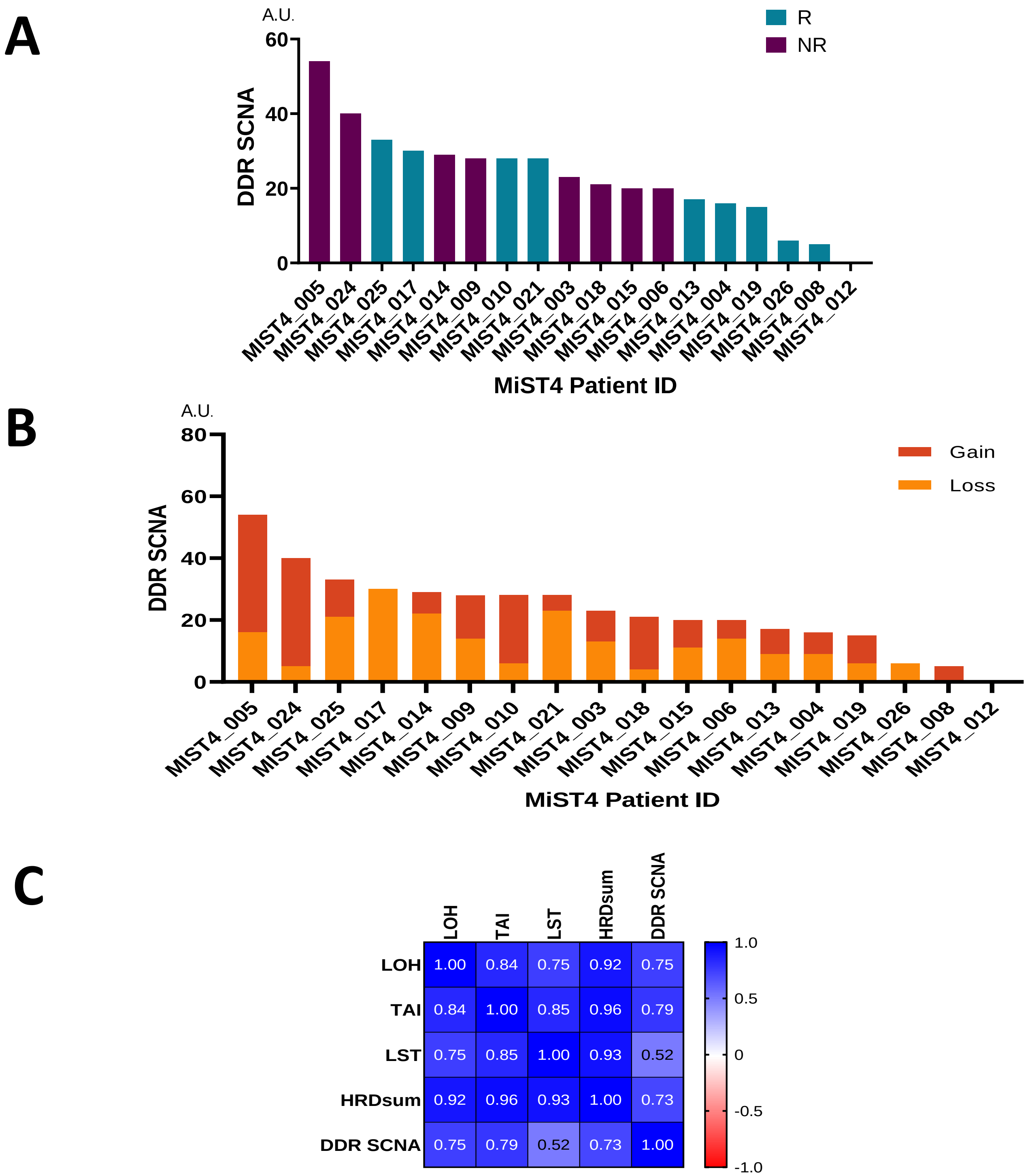

Supplementary figure 6

DDR gene alterations and HRD in the MIST4 cohort

A. Histogram showing the relative frequency of DDR copy number changes in the R-subgroup (blue) versus the NR-subgroup (purple)

B. Histogram showing the relative frequency of DDR somatic copy number alterations (losses in orange, and gains in red) across the MIST4 cohort

C. Correlation matrix showing the positive correlation homologous recombination deficiency signatures LOH, TAI and LST, the sum of these three signatures (HRDsum) and the DDR SCNA frequency

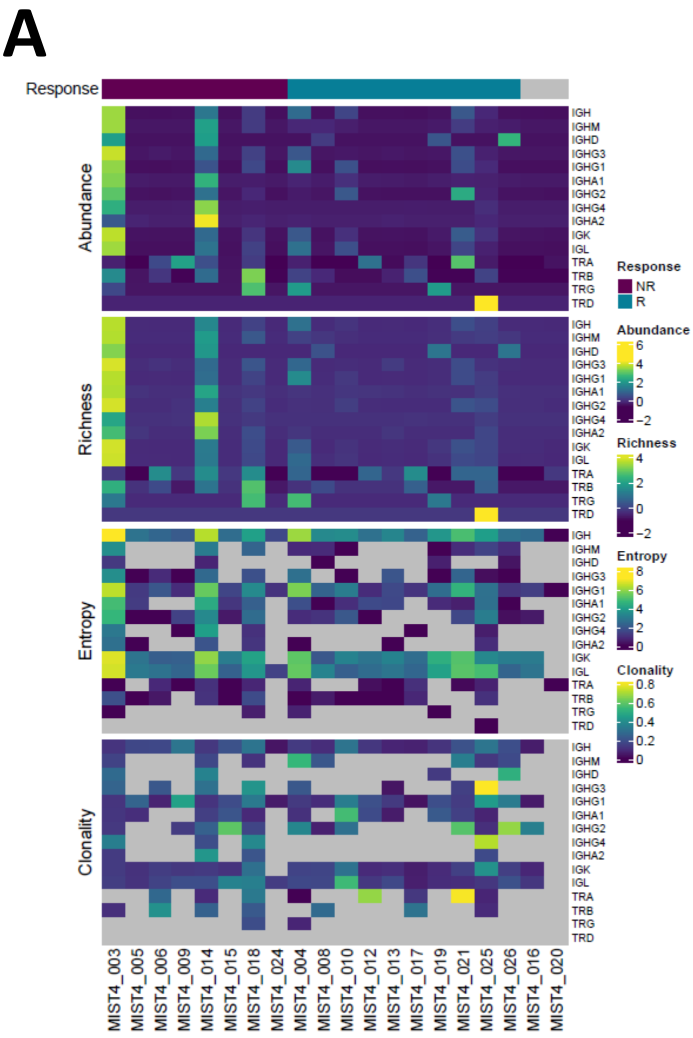

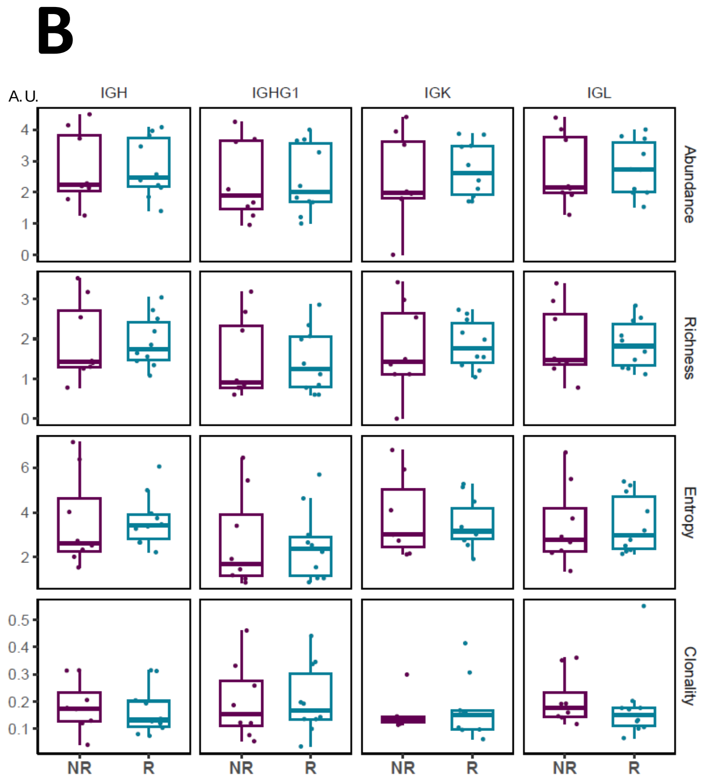

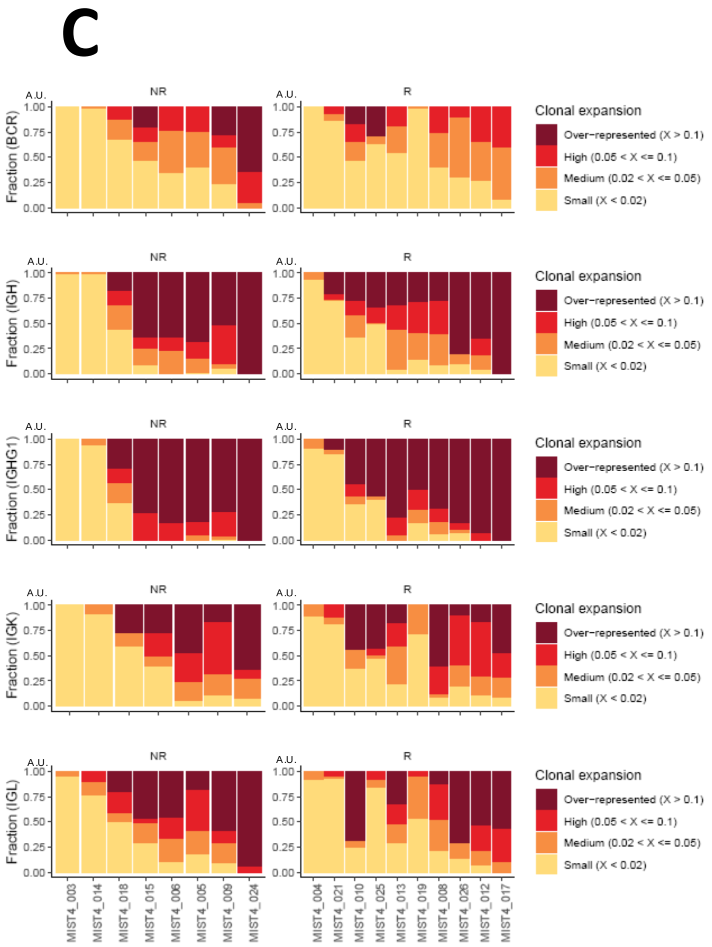

Supplementary figure 7

B and T cell clonality is not enriched in NR versus R-subgroups

A: Heatmap shows immune repertoire metrics of abundance, richness, entropy and clonality reported by TRUST4 between two groups (R vs NR). Grey indicates data that not available (NA).

B: Comparison of BCR metrics of abundance, richness, entropy and clonality in two groups (R vs NR). The boundaries of the boxes indicate the 25th and 75th percentiles, the lines within the boxes indicate the median, and the whiskers mark the range. Data were compared by two-tailed Mann–Whitney test.

C: Proportion of homeostatic space occupied by BCR clonotypes between two groups (R vs NR). Measured as a proportion taken up by over-represented (0.1–1), large (0.05–0.1), medium (0.02–0.05), and small (<0.02) clonotypes.

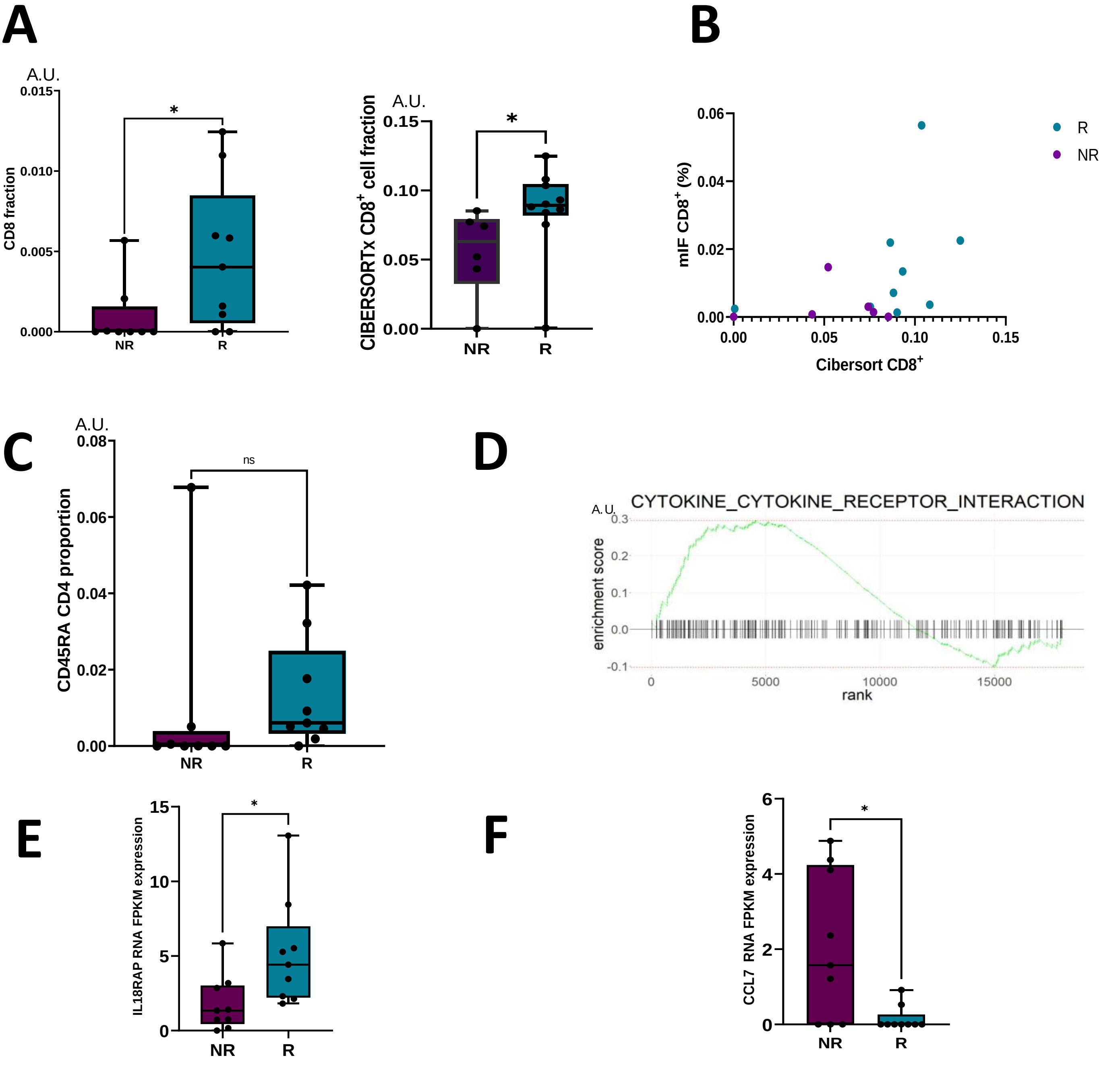

Supplementary figure 8

Immune infiltration and response in MIST4

A. Left. Box plot showing CD8+ T cell enrichment in the R vs NR subgroup. R. Corresponding immune deconvolution showing an enriched T cell fraction in R vs NR. ** denotes p<0.01

B. CD8+ T cells are correlated in immune deconvolution versus multiplex immnunofluorescence microscopy (IF) analyses

C. CD45RA CD4 cells are enriched in the R vs NR subgroup (* denotes p<0.05)

D. GSEA plot showing cytokine-cytokine receptor interaction transcriptional enrichment in R vs NR

E. IL18 RAP expression is higher in R vs NR patients

F. CCL7 expression is higher in NR versus R patients

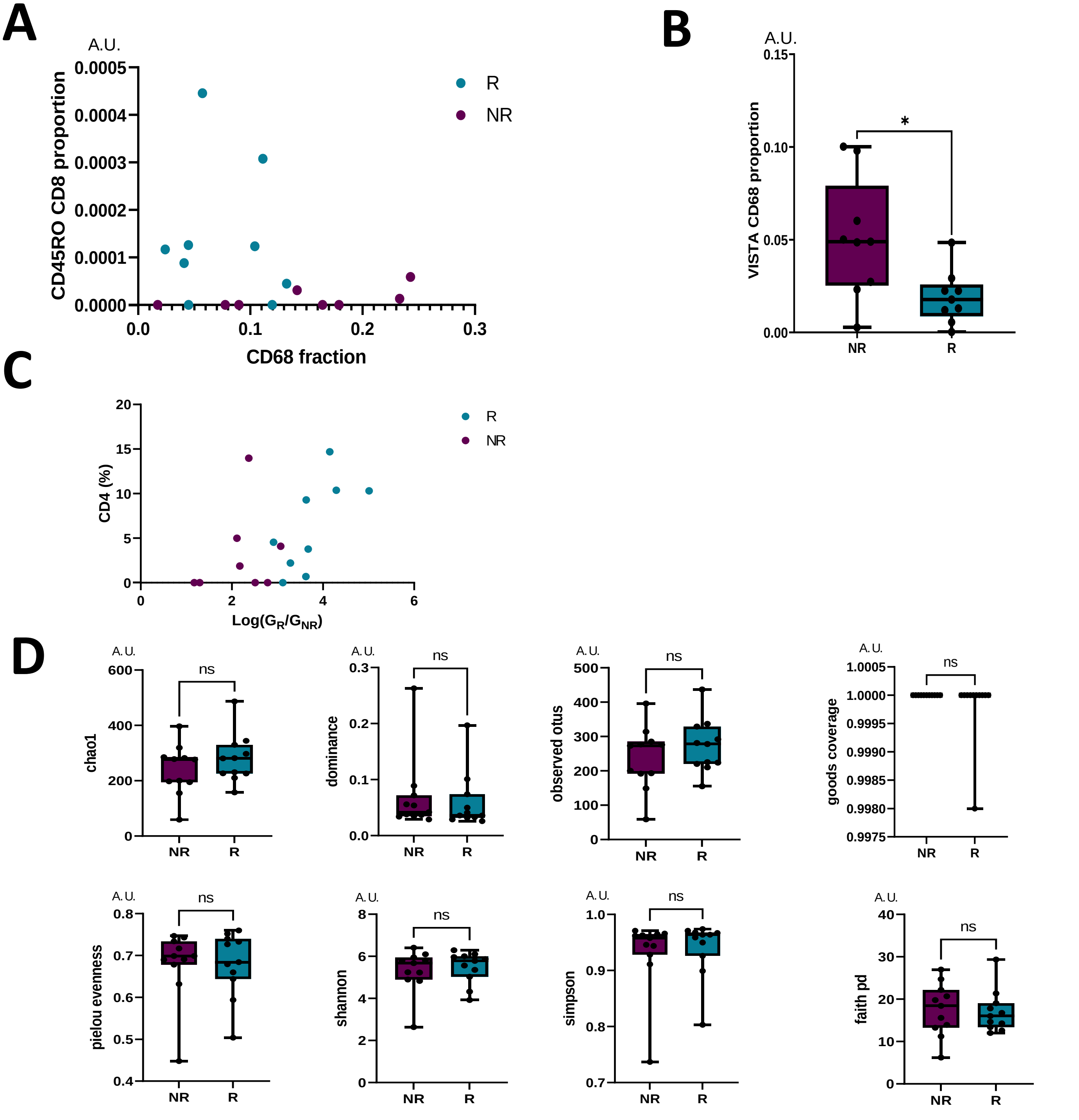

Supplementary figure 9

A. Scatter plot showing a negative correlation between antigen-experienced cytolytic T cells (CD45RO+ CD8+) and CD68 expression. R subgroup patients are shown in blue and NR in purple.

B. Box plot showing a greater proportion of VISTA +CD68 + tumour infiltrating monocytes in NR versus R patients

C. Scatter plot showing a correlation between Log$G_{R/}G_{NR}$ and CD4+ T lymphocytes. R subgroup patients are shown in blue and NR in purple.

D. Box plots comparing the alpha diversity in R vs NR subgroups measured using 8 orthogonal methods Shannon index, Simpson's index Chao1 index, Berger-Parker dominance index, Richness index (Observed-otus), Good's coverage of counts, Pielou's evenness, and Faith's phylogenetic diversity. Groups were compared using Mann Whitney test (NS=not significant, ie. p>0.05).

Characteristics	Descriptive	Allocated to Atezolizumab & Bevacizumab (n = 26)
Age (years)	N	26
	Mean[SD]	67.5 [9.2]
	Median[IQR]	68.0 [67.0, 74.0]
	Min, max	44.0, 80.0
BMI (Kg/M^2)	N	26
	Mean[SD]	25.7 [4.9]
	Median[IQR]	25.7 [21.9, 28.8]
	Min, max	17.0, 36.9
Gender	Male	18 (69.2%)
	Female	8 (30.8%)
Smoking status	Smoker	2 (7.7%)
	Non Smoker	13 (50.0%)
	Ex-smoker	11 (42.3%)
Mesothelioma subtype	Epithelioid	20 (76.9%)
	Biphasic	2 (7.7%)
	Sarcomatoid	3 (11.5%)
	NOS	1 (3.8%)
History of asbestos	No	2 (7.7%)
	Yes	16 (61.5%)
	Unknown	8 (30.8%)
ECOG status	0	4 (15.4%)
	1	22 (84.6%)
Metastases	No	19 (73.1%)
	Yes	7 (26.9%)
Primary tumour site	Thoracic	24 (92.3%)
	Abdominal	1 (3.8%)
	Pelvis	1 (3.8%)
T-stage	T1	2 (7.7%)
	T2	3 (11.5%)
	T3	7 (26.9%)
	T4	13 (50.0%)
	TX	1 (3.8%)
N-stage	N0	11 (42.3%)
	N1	6 (23.1%)
	N2	8 (30.8%)
	N3	1 (3.8%)
M-stage	M0	20 (76.9%)
	M1	6 (23.1%)
P16 negative	Not applicable	9 (34.6%)
	Positive	7 (26.9%)
	Negative	10 (38.5%)
BRCA1	Not applicable	9 (34.6%)
	Positive	11 (42.3%)
	Negative	6 (23.1%)
BAP1	Not applicable	9 (34.6%)
	Positive	4 (15.4%)
	Negative	13 (50.0%)

Supplementary Table 1.
Baseline Patient Characteristics in the MIST4 cohort
Data are n (%), median [IQR], Mean [SD], or min and max;
Abbreviations: BMI= Body Mass Index; ECOG=Eastern Cooperative Oncology Group; NOS= Not Otherwise Specified; IQR=Inter-quartile range; SD=Standard deviation

Characteristics	Descriptive	Total allocated (n = 26)
Number of prior courses of systemic anticancer therapy	One	12 (46.2%)
	Two	8 (30.8%)
	Three	4 (15.4%)
	Four	1 (3.8%)
	Five	1 (3.8%)
Best response of first line therapy	Unobtainable	1 (3.8%)
	Partial response	7 (26.9%)
	Stable disease	12 (46.2%)
	Progressive disease	6 (23.1%)
First line therapy	Pemetrexed and/or Carboplatin	16 (76.2%)
	Pemetrexed and/or Cisplatin	5 (23.8%)
Second line therapy	Pemetrexed and/or Carboplatin	3 (30.0%)
	Pemetrexed and/or Cisplatin	3 (30.0%)
	Vinorelbine	4 (40.0%)

Supplementary table 2.
Number of prior lines of therapy
Summary of prior courses of treatment received with best response to 1st line treatment and the type of systemic therapy received in both 1st and 2nd lines

Participant ID	Time on treatment (weeks)	Reason for discontinuation	Provided 12 week CT scan	Provided 24 week CT scan
**M4-001-016**	3.9	Death	No	No
**M4-001-002**	4.4	Death	No	No
**M4-001-003**	6	Disease Progression[£]	No	No
**M4-001-006**	6	Disease Progression	No	No
**M4-001-014**	7	Disease Progression	No	No
**M4-001-001**	7	Disease Progression[£]	No	No
**M4-001-015**	7	Disease Progression	No	No
**M4-005-020**	8.1	Patient Choice	No	No
**M4-005-004**	10.7	Disease Progression[£]	Yes	No
**M4-005-005**	10.7	Disease Progression[^]	Yes	No
**M4-001-018**	13	Disease Progression	Yes	No
**M4-001-009**	13.1	Disease Progression[£]	Yes	No
**M4-005-024**	13.3	Disease Progression	Yes	No
**M4-001-017**	19	Disease Progression	Yes	No
**M4-001-013**	19	Disease Progression	Yes	No
**M4-001-021**	19	Disease Progression	Yes	No
**M4-001-011**	20.3	Disease Progression	Yes	No
**M4-001-022**	25	Disease Progression	Yes	Yes
**M4-001-019**	27	Disease Progression	Yes	Yes
**M4-005-008**	32.5	Clinical decision	Yes	Yes
**M4-005-010**	39.8	Clinical decision	Yes	Yes
**M4-001-012**	48	Disease Progression	Yes	Yes
**M4-001-007**	49.1	Disease Progression	Yes	Yes
**M4-003-026**	23.7[a]	N/A	Yes	Yes
**M4-001-023**	24[a]	N/A	Yes	Yes
**M4-001-025**	24[a]	N/A	Yes	Yes

Supplementary table 3.
Summary the time on study and reasons for discontinuation
As these three patients were on treatment at data lock, their time was calculated using 24-week scan date as no discontinuation date yet.
^ indicates Clinical disease progression
£ indicates CT scan confirmed disease progression

A

	MiST-4 (n = 26)<br>n (%)
**Patients with any AEs**	23 (88.5%)
Patients with one AE	3 (11.5%)
Patients with two AEs	3 (11.5%)
Patients with three AEs	1 (3.8%)
Patients with four AEs	1 (3.8%)
Patients with five or more AEs	15 (57.7%)
**Patients without AEs**	3 (11.5%)
**Patients with grade 3 and above AEs**	9 (34.6%)

B

		MiST-4 (n = 26)<br>n (%)
Total AEs		130
By CTCAE grade		
	Missing	3 (2.3%)
	1	90 (69.2%)
	2	21 (16.2%).
	3	15 (11.5%)
	4	0 (0.0%)
	5	1 (0.8%)
By relatedness to Atezolizumab		
	Missing	11 (8.5%)
	Not Related	59 (45.4%)
	Unlikely	28 (21.5%)
	Possibly	24 (18.5%)
	Probably	8 (6.2%)
	Definitely	0 (0.0%)
By relatedness to Bevacizumab		
	Missing	12 (9.2%)
	Not Related	61 (46.9%)
	Unlikely	21 (16.2%)
	Possibly	27 (20.8%)
	Probably	9 (6.9%)
	Definitely	0 (0.0%)

Supplementary table 4.
Frequency of Adverse Events (AEs)
A. Number of AEs per patient. AEs were evaluated using the NCI CTCAE, version 4.03 are summarised by descriptive statistics. The table shows the number of patients with AEs and their percentages. 23 (88.5%) patients experienced some AEs. Among these 23 patients, there were a total of 130 AEs. The most prevalent AEs amongst all patients were fatigue occurring in 10 patients (38%) followed by vomiting in 6 patients (23%) and weight loss also in 6 patients (23%).
B. AEs by CTCAE grade and relatedness to AtzBev. Most of the AEs were CTCAE grade 1 (n=90, 69.2%) or CTCAE grade 2 AEs (n=21, 16.2%). There were 16 (12.3%) AEs with grade 3 or more which occurred in nine patients (34.6%), one of which was classified as grade 5.

	MiST-4 (n = 26)					
	Any grade	Split by individuals' highest CTCAE grade				
		Grade 1	Grade 2	Grade 3	Grade 4	Grade 5
Any AEs	23 (88%)	11 (42%)	3 (12%)	8 (31%)	0 (0%)	1 (4%)
AEs by preferred term (for AEs experienced by ≥10% of patients or with at least one event of CTCAE ≥3)						
Fatigue	8 (31%)	6 (23%)	2 (8%)	0 (0%)	0 (0%)	0 (0%)
Weight loss	5 (19%)	3 (12%)	2 (8%)	0 (0%)	0 (0%)	0 (0%)
Vomiting [a]	4 (15%)	2 (8%)	0 (0%)	1 (4%)	0 (0%)	0 (0%)
Constipation [a]	4 (31%)	1 (4%)	2 (8%)	0 (0%)	0 (0%)	0 (0%)
Headache	4 (15%)	4 (15%)	0 (0%)	0 (0%)	0 (0%)	0 (0%)
Confusion	3 (12%)	1 (4%)	1 (4%)	1 (4%)	0 (0%)	0 (0%)
Decreased appetite	3 (12%)	2 (8%)	1 (4%)	0 (0%)	0 (0%)	0 (0%)
Abdominal pain	3 (12%)	3 (12%)	0 (0%)	0 (0%)	0 (0%)	0 (0%)
Rash	3 (12%)	3 (12%)	0 (0%)	0 (0%)	0 (0%)	0 (0%)
Dyspnoea	2 (8%)	1 (4%)	0 (0%)	0 (0%)	0 (0%)	1 (4%)
Lower respiratory tract infection	2 (8%)	0 (0%)	0 (0%)	2 (8%)	0 (0%)	0 (0%)
Hypertension	2 (8%)	1 (4%)	0 (0%)	1 (4%)	0 (0%)	0 (0%)
Joint pain (Shoulder, hip or general)	2 (8%)	1 (4%)	0 (0%)	1 (4%)	0 (0%)	0 (0%)
Agitation	1 (4%)	0 (0%)	0 (0%)	1 (4%)	0 (0%)	0 (0%)
Arthralgia	1 (4%)	0 (0%)	0 (0%)	1 (4%)	0 (0%)	0 (0%)
Bowel obstruction	1 (4%)	0 (0%)	0 (0%)	1 (4%)	0 (0%)	0 (0%)
Dehydration	1 (4%)	0 (0%)	0 (0%)	1 (4%)	0 (0%)	0 (0%)
GI Bleed	1 (4%)	0 (0%)	0 (0%)	1 (4%)	0 (0%)	0 (0%)
Hypophosphatemia	1 (4%)	0 (0%)	0 (0%)	1 (4%)	0 (0%)	0 (0%)
Vena Cava Obstruction	1 (4%)	0 (0%)	0 (0%)	1 (4%)	0 (0%)	0 (0%)

Supplementary table 5.
AEs summarised by symptoms

Adverse events that either happened in at least 3 patients (>= 10%) or were grade 3 or above by CTCAE grade and preferred term. Data are presented as number (n) and percentage (%) of participants

AE, adverse event. a highest-grade column does not add to the total having any grade as one individual has grade missing.

AE System Organ	AE Term	N (%)
Blood and Lymphatic system	Anaemia	2 (1.5%)
	Raised INR	1 (0.8%)
Cardiac disorders	Vena Cava Obstruction	1 (0.8%)
	ECG suggests inferior myocardial infarction, age undetermined	1 (0.8%)
Endocrine disorders	Hyperthyroidism	1 (0.8%)
Eye disorders	conjunctivitis	1 (0.8%)
	Allergic reaction	1 (0.8%)
	dry eyes - intermittent	1 (0.8%)
	Eye infection	1 (0.8%)
Gastrointestinal disorders	Diarrhoea	1 (0.8%)
	Melaena	1 (0.8%)
	GI Bleed	1 (0.8%)
	Mucositis	2 (1.5%)
	Rectal haemorrhage	1 (0.8%)
General disorders and administration site conditions	Abdominal pain	3 (2.3%)
	Constipation	4 (3.1%)
	Oropharyngeal haemorrhage	1 (0.8%)
	Tonsillitis	2 (1.5%)
	Ache around operation site in chest	1 (0.8%)
	Bowel obstruction	1 (0.8%)
	Shin feeling sensitive	1 (0.8%)
	Urine Leukocytes Ca500	1 (0.8%)
	Toothache	1 (0.8%)
	Urine PH 6	1 (0.8%)
	Swelling in thumb	1 (0.8%)
	Vein irritation during infusion	1 (0.8%)
	Changes in vision	1 (0.8%)
	Nausea	2 (1.5%)
	Vomiting	6 (4.6%)
	Fatigue	10 (7.7%)
	Proteinuria	1 (0.8%)
	Epistaxis	2 (1.5%)
	Dry mouth	2 (1.5%)
	Non cardiac chest pain	2 (1.5%)
	General pain	1 (0.8%)
	Joint pain (Shoulder, hip and/or general)	4 (3.1%)
	Mouth ulcer	1 (0.8%)
	increased pain, left nipple	1 (0.8%)
	Haematuria	1 (0.8%)
Hepatobiliary disorders	immunotherapy related hepatitis	1 (0.8%)
Infections and Infestations	Cellulitis	1 (0.8%)
	Shingles	1 (0.8%)
	Upper Respiratory Infection	1 (0.8%)

Supplementary table 6.
AEs summarised by system

Supplementary table 6 continued

AE System Organ	AE Term	N (%)
	Lower respiratory tract infection	2 (1.5%)
Investigations	Increased AST	2 (1.5%)
	Lymphocyte count decreased	1 (0.8%)
	Raised Alkaline Phosphatase (intermittent)	1 (0.8%)
	hypomagnesaemia	1 (0.8%)
	Weight loss	6 (4.6%)
	Raised creatinine	3 (2.3%)
	Increased ALT	1 (0.8%)
Metabolism and Nutrition disorders	hypoalbuminaemia	2 (1.5%)
	hypercalcaemia	1 (0.8%)
	Anorexia	1 (0.8%)
	Hyponatremia	1 (0.8%)
	Decreased appetite	3 (2.3%)
	Hypophosphatemia	1 (0.8%)
	Dehydration	1 (0.8%)
Musculoskeletal and connective tissue disorders	Tingling in feet/hands	2 (1.5%)
	Arthralgia	1 (0.8%)
	Back Pain	1 (0.8%)
Nervous system disorders	Drowsy	1 (0.8%)
	Dizziness	1 (0.8%)
	Headache	4 (3.1%)
Psychiatric disorders	Agitation	1 (0.8%)
	Confusion	4 (3.1%)
Renal and urinary disorders	Urine Infection	1 (0.8%)
	Urinary tract infection	2 (1.5%)
Respiratory, thoracic and mediastinal disorders	Nasal congestion	1 (0.8%)
	Hoarseness of voice	1 (0.8%)
	Dyspnoea	2 (1.5%)
Skin and subcutaneous tissue disorders	Lesion on scalp solar keratosis	1 (0.8%)
	nail discolouration	1 (0.8%)
	Rash	3 (2.3%)
	Pruritus	1 (0.8%)
Vascular disorders	Hypertension	2 (1.5%)
	Hypotension	1 (0.8%)

Data are presented as number (n) and percentage (%) of AEs

AE, adverse event

Supplementary table 6 continued.
AEs summarised by system

A

	MiST-2 (n = 26)<br>n (%)
**Patients with SAEs**	**11 (42.3%)**
Patients with one SAE	6 (32.1%)
Patients with two SAEs	4 (15.4%)
Patients with three SAEs	1 (3.8%)
Patients with more than three SAEs	0 (0%)
**Patients without SAEs**	**15 (57.7%)**
Patients with SAE leading to treatment discontinuation^	4 (15.4%)
Patients with SAE leading to trial withdrawal	0 (0%)
Patients with SAE leading to patient death	1 (3.8%)

^ 3 patients had SAEs leading to permanent treatment discontinuation of both Atezolizumab and Bevacizumab, 1 patient had SAEs that resulted in permanent treatment discontinuation of Bevacizumab and temporary treatment discontinuation of Atezolizumab.

Data are presented as number (n) and percentage (%) of participants

SAE; Serious adverse events

B

**Any SAEs**	**17**
Related to Atezolizumab	
Yes	7 (41.2%)
No	10 (58.8%)
Related to Bevacizumab	
Yes	8 (47.1%)
No	9 (52.9%)
Final grade of SAE	
Grade 1	1 (5.9%)
Grade 2	1 (5.9%)
Grade 3	14 (82.4%)
Grade 5	1 (5.9%)
Final outcome	
Hospitalisation	16 (94.1%)
Fatal	1 (5.9%)

Data are presented as number (n) and percentage (%) of AEs

SAE; Serious adverse events

Supplementary table 7.
Serious adverse events (SAEs)

1.SAEs summarised by frequency

2.SAE relatedness to either atezolizumab or bevacizumab including the final grade of SAE

A

Trial ID	SAE term	CTCAE Grade	Seriousness	Atezolizumab Causality	Bevacizumab Causality	Atezolizumab Expectedness	Bevacizumab Expectedness	Outcome
M4-001-001	Vomiting	3	In-patient hospitalisation or prolongation of existing hospitalisation	Related	Related	Expected	Expected	Resolved
M4-001-001	Vomiting	3	In-patient hospitalisation or prolongation of existing hospitalisation	Related	Related	Expected	Expected	Resolved
M4-001-002	Superior Vena Cava Obstruction	3	In-patient hospitalisation or prolongation of existing hospitalisation	Unrelated	Unrelated	N/A	N/A	Resolved with sequelae
M4-001-003	?GI bleed	3	In-patient hospitalisation or prolongation of existing hospitalisation	Unrelated	Related	N/A	Expected	Resolved
M4-001-002	Breathlessness/dyspnoea	5	Resulted in death	Unrelated	Unrelated	N/A	N/A	Fatal
M4-005-004	Dehydration	3	In-patient hospitalisation or prolongation of existing hospitalisation	Unrelated	Unrelated	N/A	N/A	Resolved
M4-005-005	Pain in left hip	3	In-patient hospitalisation or prolongation of existing hospitalisation	Unrelated	Unrelated	N/A	N/A	Resolved
M4-001-013	Bone pain	3	In-patient hospitalisation or prolongation of existing hospitalisation	Related	Unrelated	Expected	N/A	Resolved
M4-001-013	Lower respiratory tract infection	3	In-patient hospitalisation or prolongation of existing hospitalisation	Unrelated	Unrelated	N/A	N/A	Resolved
M4-001-013	Immunotherapy related hepatitis	1	In-patient hospitalisation or prolongation of existing hospitalisation	Related	Unrelated	Expected	N/A	Resolved
M4-005-020	Confusion	3	In-patient hospitalisation or prolongation of existing hospitalisation	Unrelated	Unrelated	N/A	N/A	Resolved
M4-005-020	Bone pain	2	In-patient hospitalisation or prolongation of existing hospitalisation	Related	Related	Expected	Expected	Resolved with sequelae
M4-001-012	Diarrhoea	3	In-patient hospitalisation or prolongation of existing hospitalisation	Related	Related	Expected	Expected	Resolved
M4-005-010	Meningo encephalitis	3	In-patient hospitalisation or prolongation of existing hospitalisation	Related	Related	Unexpected	Unexpected	Resolved
M4-001-021	Acute atrial fibrilation	3	In-patient hospitalisation or prolongation of existing hospitalisation	Unrelated	Unrelated	N/A	N/A	Resolved
M4-001-019	Enterovesical fistula	3	In-patient hospitalisation or prolongation of existing hospitalisation	Unrelated	Related	N/A	Expected	Resolved
M4-001-019	Small Intestinal Obstruction	3	In-patient hospitalisation or prolongation of existing hospitalisation	Unrelated	Related	N/A	Expected	Resolved

B

DDR Genes (95)									
ABL1	BRCA2	ERCC4	FANCF	MDC1	RAD1	RAD54L	RNF169	SLX1B	USP1
ABL2	BRIP1	EXO1	FANCG	MRE11	RAD17	RAD9A	RNF2	SLX4	WRN
ARID1A	CDK12	FAAP100	FANCI	MUS81	RAD50	RBBP8	RNF4	SMC3	XRCC2
ATM	CHEK1	FAAP20	FANCL	NBN	RAD51	RECQL	RPA1	SMC5	XRCC3
ATR	CHEK2	FAAP24	FANCM	PALB2	RAD51AP1	RECQL4	RPA2	SPIDR	GEN1
ATRIP	DMC1	FAN1	FEN1	PARP1	RAD51B	REV1	RPA3	TIMELESS	
BAP1	DNA2	FANCA	FIGNL1	PIAS1	RAD51C	RIF1	RTEL1	TIPIN	
BARD1	DNMT3A	FANCC	H2AFX	PIAS4	RAD51D	RMI1	SETMAR	TOPBP1	
BLM	EME1	FANCD2	HELQ	POLN	RAD52	RMI2	SFR1	TP53BP1	
BRCA1	EME2	FANCE	KAT5	POLQ	RAD54B	RNF168	SLX1A	UIMC1	

Altered more than once

Altered once

C

Genera	p value	
	Random Forests	LEfSE
Prevotella	0.0025	0.007740253
Butyricicoccus	0.0281	0.006428241
Eubacterium ventriosum group	0.0053	0.016924699
Erysipelatoclostridium	0.0187	0.008814655
Bilophila	0.0188	0.032784246

Supplementary table 8

1.Summary of all SAEs classified by SAE term

2.DNA damage response gene set highlighting and wildtype and somatic alterations

3.Enriched genera and associated p values random forest p values, and LEfSE

Mesothelioma Stratified Therapy (MiST):

A stratified multi-arm phase IIa clinical trial to enable accelerated evaluation of targeted therapies for relapsed malignant mesothelioma

MiST4
Atezolizumab and Bevacizumab in PD-L1 positive relapsed mesothelioma

Sponsor Reference No: 0627

Ethics Ref: 18/EM/0118

Eudract Number: 2017-003353-41

ISRCTN: 39816629

Date and Version No: Version: 1.0, dated 14.06.2019

Chief Investigator:	Professor Anne Thomas PhD FRCP<br>Director, Leicester Cancer Research Centre<br>Professor of Cancer Therapeutics
Sponsor:	University of Leicester
Funder:	The British Lung Foundation
Signatures:	The approved protocol should be signed by author(s) and/or person(s) authorised to sign the protocol

Confidentiality Statement

All information contained within this protocol is regarded as, and must be kept confidential. No part of it may be disclosed by any Receiving Party to any Third Party, at any time, or in any form without the express written permission from the Chief Author/Investigator and / or Sponsor.

AUTHORS

Professor Dean Fennell PhD FRCP

Professor of Thoracic Medical Oncology

Director, Leicester Mesothelioma Research Programme

Amy King MSc

Manager, Leicester Mesothelioma Research Programme

Dr Harriet Walter PhD MRCP
Medical Oncology Consultant

Professor Anne Thomas PhD FRCP

Professor of Cancer Therapeutics

Director, Leicester Cancer Research Centre

SIGNATURE PAGE

Chief Investigator Name: **Professor Anne Thomas**

Chief Investigator signature: ____

Date: ____

Sponsor Representative Name: ____

Sponsor Representative signature: ____________________________________

Date: ____

Principal Investigator Name:

Principal Investigator signature: ______________________________________

Date: ____

ABBREVIATIONS

Term	Definition
AE	Adverse Event
AR	Adverse Reaction
ARD	Asbestos Related Disease
BAP1	BRCA1 associated protein-1
BID	Twice Daily
BLF	British Lung Foundation
CF	Consent Form
CI	Chief Investigator
CNV	Copy Number Variation
CNS	Central Nervous System
Cohort	Molecular Cohort
CR	Complete Response
CRF	Case Report Form
CRN	Clinical Research Network
CT Scan	Computed tomography
CTA	Clinical Trials Authorisation
CV	Curriculum Vitae
DCR	Disease control rate
DMC	Data Monitoring Committee
DPA	Data Protection Act
DSMC	Data Safety Monitoring Committee
EC	Ethics Committee
eCRF	Electronic Case Report Form
EME	Efficacy Mechanism Evaluation
EudraCT	European Union Drug Regulatory Agency Clinical Trial
EU	European Union
FDA	Federal Drugs Administration
FFPE	Formalin Fixed Paraffin Embedded
GCP	Good Clinical Practice
HDPE	High Density Polyethylene
HRA	Health Research Authority
ICH	International Conference on Harmonisation
ID	Identification
IDMC	Independent Data Monitoring Committee
IMP	Investigational Medicinal Product
LCTU	Leicester Clinical Trials Unit
LOH	Loss of Heterozygosity
MiST	Mesothelioma Stratified Therapy
MHRA	Medicines and Healthcare products Regulatory Agency
MREC	Main Research Ethics Committee
MPM	Malignant Pleural Mesothelioma
NCRI	National Cancer Research Institute
NHS	National Health Service
NIHR	National Institute for Health Research
NIHR CRN	National Institute for Health Research Clinical Research Network

ONS	Office of National Statistics
PD	Progressive Disease (RECIST)
PD-L1	Programmed Death-Ligand 1
PFS	Progression-Free Survival (the primary outcome)
PI	Principal Investigator
PIC	Patient Identification Centre
PIS	Patient Information Sheet
PR	Partial Response (RECIST)
PS	Performance Status
QA	Quality Assurance
QC	Quality Control
QL	Quality of Life
R&D	Research and Development
REC	Research Ethics Committee
Modified RECIST 1.1	Response Evaluation Criteria 1.1 In Solid Tumours
RGC	Research Governance Committee
RGF	Research Governance Framework (for Health and Social Care)
SAE	Serious Adverse Event
SD	Stable Disease (modified RECIST)
SOP	Standard Operating Procedure
SUSAR	Suspected unexpected serious adverse reaction
TM	Trial Manager
TMF	Trial Master File
TMG	Trial Management Group
TSC	Trial Steering Committee
US	Ultrasound
UoL	University of Leicester (Sponsor)
WHO	World Health Organization
WOCBP	Women of Child Bearing Potential

AMENDMENT HISTORY

Amendment No.	Protocol Version No.	Date issued	Author(s) of changes	Details of Changes made

List details of all protocol amendments here whenever a new version of the protocol is produced

TRIAL SYNOPSIS

**Study Title**	**MiST4**<br>Atezolizumab and Bevacizumab in PD-L1 positive relapsed mesothelioma
**Internal ref. no.**	0627
**Clinical Phase**	Drug development phase IIa
**Trial Design**	Two stage, single arm phase IIa clinical trial of atezolizumab and bevacizumab in relapsed mesothelioma.
**Trial Participants**	PD-L1 positive relapsed mesothelioma patients in patients previously treated with platinum doublet therapy
**Planned Sample Size**	26
**Safety Follow-up duration**	30 days after patient's last dose
**Follow up duration**	6 months after patient's last dose
**Planned Trial Period**	18 months
**Primary Objective**	To establish the 12 week disease control rate of atezolizumab/bevacizumab in patients with PD-L1 positive mesothelioma
**Secondary Objectives**	To establish the safety, toxicity and objective response rate, and 24 week disease control rate of atezolizumab/bevacizumab in patients with PD-L1 positive relapsed mesothelioma.
**Primary Endpoint**	Disease control rate at 12 weeks
**Secondary Endpoints**	• Safety and toxicity<br>• Objective response rate measured by modified RECIST 1.1 at 24 weeks<br>• Disease control rate at 24 weeks
**Translational Studies**	Genome wide copy analysis of tumour tissue will be conducted to interrogate the genomic correlates of exceptional response to atezolizumab/ bevacizumab.<br><br>Immune regulated gene signature. Tumour samples will have RNA extracted, and gene expression analysis will be conducted to correlate the impact of tumour infiltrating lymphocytes on response.<br><br>On obtaining patient consent, research blood samples of plasma and serum will be collected prior to treatment and following disease progression. These samples will be used to support future correlative studies.<br><br>To assess correlation between gut microbiome composition and response.
**Investigational Medicinal Products**	Atezolizumab and bevacizumab supplied by Roche/Genentech.
**Form**	Intravenous administration
**Dose**	• Atezolizumab will be given as a fixed dose of 1200 milligrams (mg) via intravenous (IV) infusion on day 1 of a 21-day cycle.<br>• Bevacizumab will be given as 15 milligrams per kilogram (mg/kg) via IV infusion on Days 1 of a 21-day cycle.
**Route**	Intravenous administration

SCHEDULE OF EVENTS:

	Screening / Baseline (Up to - 28 days)	*Cycle 1 Day 1 (- 3 days)	Cycle 1 Day 15 (+/- 3 days)	*Cycle 2 Day 1 (+/- 3 days)	Cycle 2 Day 15 (+/- 3 days)	*Cycle 3 Day 1 (+/- 3 days)	*Cycle 4 Day 1 (+/- 3 days)	*Cycle 5 Day 1 (+/- 3 days)	*Cycle 6 Day 1 (+/- 3 days)	*Cycle 7 Day 1 (+/- 3 days)	*Cycle 8 Day 1 (+/- 3 days)	Cycle 8 Day 21 (EoT) (+/- 3 days)	Disease Progressio n (+/-7 days)	30 Day Follow Up (+/-7 days)	6 month Follow Up (+/-7 days)
Informed Consent	X														
Medical History	X														
Inclusion & Exclusion Criteria	X														
Physical Examination	X	X		X		X	X	X	X	X	X		X	X	X
Vital Signs (Including height (baseline only), weight, temperature, blood pressure, heart rate and respiratory frequency)	X	X[a]		X[a]		X[a]	X[a]	X[a]	X[a]	X[a]	X[a]	X	X	X	X
Concomitant Medication Record	X	X		X		X	X	X	X	X	X		X	X	X
Full Blood Count	X[b]	X[b]	X[b]	X[b]	X[b]	X[b]	X[b]	X[b]	X[b]	X[b]	X[b]		X	X	
Biochemistry Profile (Including: Urea, Electrolytes, calcium phosphase and Liver Function Test, Thyroid function test) LDH & CRP to be included at baseline only)	X[b]	X[b]	X[b]	X[b]	X[b]	X[b]	X[b]	X[b]	X[b]	X[b]	X[b]		X	X	
Pregnancy Test	X[c]	X	X	X	X	X	X	X	X	X	X			X	
Urinalysis	X	X		X		X	X	X	X	X	X		X	X	
CT Scan	X[d]				X[e]			X[e]				X[f]	X[f]		
Scan results reported to Modified RECIST 1.1 Criteria.	X				X[e]			X[e]				X[e]	X[e]		

	Screening / Baseline (Up to -28 days)	*Cycle 1 Day 1 (- 3 days)	Cycle 1 Day 15 (+/- 3 days)	*Cycle 2 Day 1 (+/- 3 days)	Cycle 2 Day 15 (+/- 3 days)	*Cycle 3 Day 1 (+/- 3 days)	*Cycle 4 Day 1 (+/- 3 days)	*Cycle 5 Day 1 (+/- 3 days)	*Cycle 6 Day 1 (+/- 3 days)	*Cycle 7 Day 1 (+/- 3 days)	*Cycle 8 Day 1 (+/- 3 days)	Cycle 8 Day 21 (EoT) (+/- 3 days)	Disease Progressio n (+/-7 days)	30 Day Follow Up (+/-7 days)	6 month Follow Up (+/-7 days)
ECG	X	X[g]		X[g]		X[g]	X[g]	X[g]	X[g]	X[g]	X[g]				
Research Blood	X												X		
Archival Tissue Biopsy	X														
Research biopsy of tissue (optional)													X[h]		
Stool collection/microbio me	X														
Drug Administration		X		X		X	X[i]	X	X	X	X[i]				
Toxicity Assessment		X		X		X	X	X	X	X	X		X	X	X
Survival Status														X	X

<u>* Cycle duration is 21 days</u>

Xa = Vital signs required pre and post dose of each administration of atezolizumab
Xb = To be conducted no more than 72 hours prior to treatment
Xc = Serum pregnancy test required
Xd = Standard CT scan results can be used unless scan was conducted over 28 days before study entry. After 28 days a re-scan will be required for research purposes.
Xe = To be conducted every 6 weeks – (can be conducted +/- 7 days of the scheduled CT scan)
Xf = Scan to be conducted at end of treatment (EoT) or at Disease Progression (DP) – whichever comes first.
Xg = ECG required at baseline. To continue ECG investigation at day 1 of each cycle **<u>only</u>** if clinically indicated
Xh = Optional biopsy – check consent form
Xi= Patient may remain on atezolizumab/bevacizumab after cycle 8 on a named patient basis if seen to be receiving clinical benefit – Clinicians decision. **Note to study teams:** If patients continue to be benefitting from atezolizumab/bevacizumab at cycle 4, please commence the “special needs request” documentation in preparation for the provision of treatment post cycle 8.

Contents

AUTHORS II

SIGNATURE PAGE III

ABBREVIATIONS IV

AMENDMENT HISTORY VI

TRIAL SYNOPSIS VII

SCHEDULE OF EVENTS: VIII

1. INTRODUCTION & SCIENTIFIC BACKGROUND 1

1.1 Disease setting 1

1.2 Background underpinning the proposed research 2

2. OBJECTIVES 7

2.1 Primary Objective 7

2.2 Secondary Objectives 7

2.3 Exploratory Objectives 7

3. STUDY DESIGN 8

3.1 Summary of trial design 8

3.2 Flow chart of trial design 10

3.3 Primary Endpoint/Outcome Measures 11

3.3.1 Primary Endpoint/Outcome Measure 11

3.3.2 Secondary Endpoints/Outcome Measures 11

4. TRIAL PARTICIPANTS 12

4.1 Overall description of trial Patients 12

4.2 Inclusion Criteria 13

4.3 Exclusion Criteria 15

5. STUDY PROCEDURES 21

5.1 Informed Consent 21

5.2 Screening and Eligibility Assessment 21

6. TRIAL ASSESSMENTS 22

6.1 Baseline Assessments 22

6.2 Assessments on Treatment 23

6.3 End of Treatment (Cycle 8 – Day 21). 26

6.4 Assessments at Disease Progression 26

6.5 Follow Up 30 Days Post Progression/withdrawal OR End of 8 Cycles of Treatment 27

6.6 Follow-up Assessment 6 Months after Disease Progression/End of treatment 28

7. COMPLETION OF CRFS 29

8. DEFINITION OF END OF TRIAL 30

9. DISCONTINUATION/WITHDRAWAL OF PARTICIPANTS FROM trial TREATMENT 31

10. LOST TO FOLLOW-UP 33
11. TREATMENT OF TRIAL PARTICIPANTS 34
11.1 Description of Atezolizumab 34
11.1.1 Preparation and Administration of Atezolizumab 34
11.2 Description of Bevacizumab 35
11.2.1 Preparation and Administration of Bevacizumab 35
11.3 Criteria for re-treatment with both agents: 36
12. DOSE MODIFICATIONS AND DELAYS 38
12.1 Atezolizumab 38
12.1.1 Atezolizumab dose modifications: 38
12.1.2 Management of Atezolizumab specific adverse events 38
12.1.3 Ocular Events 39
12.1.4 Dermatological Events 40
12.1.5 Renal Events 42
12.1.6 Systemic immune activation 43
12.2 Management of overdoses 48
12.3 Bevacizumab 48
12.3.1 Bevacizumab dose and dose interval modification 48
12.4 Pregnancy or Drug Exposure during Pregnancy 53
13. PRIOR AND CONCOMITANT THERAPIES 57
13.1 Atezolizumab 58
13.1.1 Concomitant, Anticancer or Experimental Therapy 58
13.1.2 Excluded and cautionary therapy for atezolizumab treated patients 58
13.1.3 Hematopoietic Growth Factors and Blood Products 59
13.1.4 Drug interactions 59
13.1.5 Bisphosphonates 59
13.1.6 Other Concomitant Medications 59
13.2 Bevacizumab 60
13.2.1 Concomitant, Anticancer or Experimental Therapy 60
13.2.2 Other Concomitant Medications 60
14. DOSAGE, FORM, PACKAGING, AND LABELLING OF IMP. 61
14.1 Storage of study treatment 61
14.2 Accountability of the Study Treatment 61
15. SAFETY REPORTING 63
15.1 Serious Adverse Event 63
15.1.1 Definitions 63
15.2 Safety Assessments 67
15.3 Clinical Laboratory Assessments as Adverse Events 68
15.4 Causality and Expectedness Assessments 68

15.4.1 15.4.1 Reference Safety information 68
15.4.2 Causality 68
Outcome and Action Taken for an Adverse Event 70
15.7 Pregnancy or Drug Exposure During Pregnancy 72
16. STATISTICS 74
16.1 Primary Outcome 74
16.2 Hypothesis Tested 74
17. DIRECT ACCESS TO SOURCE DATA/DOCUMENTS 77
18. QUALITY CONTROL AND QUALITY ASSURANCE PROCEDURES 78
19. CODES OF PRACTICE AND REGULATIONS 79
19.1 Ethics 79
19.2 Sponsor Standard Operating Procedures 79
19.3 Declaration of Helsinki 79
19.4 ICH Guidelines for Good Clinical Practice 79
19.5 Approvals 79
19.6 Participant Confidentiality 80
20. SPONSORSHIP 81
20.1 Insurance & Indemnity 81
21. UNIVERSITY OF LEICESTER DELEGATED RESPONSIBILITIES 82
21.1 University of Leicester has delegated the following responsibilities to the Chief Investigator: 82
21.2 Responsibilities Delegated to the University Hospitals of Leicester NHS Trust and the University of Leicester Laboratory 82
22. DATA HANDLING AND RECORD KEEPING 83
23. STUDY GOVERNANCE 84
23.1 Trial Management Group (TMG) 84
23.2 Trial Steering Committee (TSC) 84
23.3 Data Safety Monitoring Committee (DSMC) 84
24. TRANSLATIONAL RESEARCH 85
25. FINANCE 86
26. PUBLICATION POLICY 87
27. REFERENCES 88
28. APPENDIX A: 90
29. APPENDIX B: 93

1. INTRODUCTION & SCIENTIFIC BACKGROUND

1.1 Disease setting

Mesothelioma

Mesothelioma is an aggressive malignancy associated in the majority of cases with prior asbestos exposure. It arises from mesothelial cells within the parietal or visceral pleura of the thorax or peritoneum and is commonly described as a locally infiltrative disease; pleural mesothelioma may invade the chest wall, mediastinum, pericardium and diaphragm. Whilst nodal metastases can commonly occur, metastatic spread has been less commonly described.

The Mesothelioma Epidemic

Although considered a relatively rare disease, the incidence of mesothelioma in the UK is the highest in the world and is increasing (3.4/100,000 compared with 2.3/100,000 in France and 3.2/100,000 in the Netherlands). In the UK, 65,000 people are expected to die by 2050 (after 2001), making mesothelioma one of the few predicted cancer epidemics. In 2012 the incidence was 2535 deaths per year. The World Health Organisation (WHO) estimates asbestos-related disease (ARD) accounts for 92,250 deaths per year globally, a greater than doubling from 1994-2008 [1], and the continued use of asbestos in the developing world could lead to a global epidemic of mesothelioma.

Paucity of Effective Therapy for Mesothelioma

Mesothelioma is an apoptosis resistant cancer [2]. There have been no new approved treatments for mesothelioma for over 12 years. Single agent chemotherapy has shown limited efficacy in MPM, with disappointing response rates (RRS) of <20%. The first chemotherapy combination of cisplatin and pemetrexed was approved in 2004, based on the EMPHACIS phase III randomised trial of pemetrexed/cisplatin. This trial enrolled 456 patients, demonstrating with the combination of cisplatin and pemetrexed improved overall survival (12.1 months as compared to 9.3 months in the cisplatin only arm ($p=0.02$, 2-sided log rank test), hazard ratio 0.77) [3]. Progression free survival increased from 3.9 to 5.7 months ($p=0.01$) and a significant increase in RR (41.3% vs 16.6%, $P<0.0001$) was observed. The combination remains the only National Comprehensive Center Network category 1 recommendation for patients with MPM who can tolerate both drugs. For patients who are unable to tolerate cisplatin, the combination of carboplatin and pemetrexed has shown similar outcomes in Phase II trials, with median OS of 12–14 months.

There is currently no standard option for relapsed mesothelioma, despite the several non-randomised phase II trials conducted over the last 3 decades [4]. Individual patient meta-analysis of 16 clinical trials did however show that radiological response is an independent predictor of longer survival [5]. Accordingly, increasing response rates may improve overall survival, either through identification of agents with improved pro-apoptotic efficacy or molecular stratification of therapy, as evidenced in the management of non-squamous lung cancer[6-8] [9, 10]

1.2 Background underpinning the proposed research

Immune checkpoint blockade as molecular target

The landscape of cancer therapy has been recently transformed by the emergence of immunotherapy involving the targeting of immune checkpoints [11-20]. Programmed cell death 1 (PD-1) is a 55kDa transmembrane inhibitory immunoreceptor expressed by activated T cells that negatively regulates immune responses required for peripheral self-tolerance [21].

PD-1 interacts with its ligand PD-L1, a member of the B7 gene family [22]. The PD-1-PD-L1 axis mediates an inhibitory signal to T cells leading to induction of apoptosis via PD-1 activation [23, 24]. Blockade of PD-1 or PD-L1, de-represses T cell activation, unleashing a clinical immune response with tumour regression [25-27].

Atezolizumab

Atezolizumab is a fully humanized, non glycolysated PD-L1 monoclonal antibody of IgG1 isotype, consisting of 2 heavy chains (448 amino acids) and 2 light chains (214 amino acids). Single amino acid substitution at position 298 on the heavy chain, results in minimal binding to the Fc receptor and results in no detectable Fc-effector function. Atezolizumab targets programmed death-ligand (PD-L1) on tumour infiltrating cells (TICs) and tumour cells (TCs), preventing interaction with its receptors Programmed Death 1 (PD-1) and B7.1 (CD80), present on T cells and other immune cells.

PD-L1 is expressed by tumours and expression is over-expression in certain cancer types, has been associated with a poor prognosis. Atezolizumab is currently being evaluated in clinical trials as an immunotherapy for several types of solid tumours and haematological malignancies by Genentech/Roche. In May 2016 Atezolizumab gained FDA approval for bladder cancer treatment and subsequent approval in October 2016 was granted in metastatic non-small cell lung cancer (NSCLC) in patients progressing after a platinum containing regimen.

Proof of concept: PD-L1 inhibitors exhibited efficacy in mesothelioma

PD-L1 blockade has demonstrated promising efficacy in patients with mesothelioma (updated results ASCO 2016). In a phase 1b study, 53 patients were treated with avelumab, with histologically or cytologically confirmed unresectable mesothelioma (pleural or peritoneal) that progressed after prior platinum-pemetrexed–containing regimen or platinum-based regimen followed by pemetrexed (MSB0010718C, EMD Serono, NCT01772004). Avelumab was administered at a dose of 10mg/kg two weekly until confirmed progression, unacceptable toxicity, or any criteria for withdrawal occurred. Median progression free

survival was 17.1 weeks (95% CI: 6.1-30.1). Patients had received a median of 1.5 prior treatments (range, 0-7-4). Histology was epithelial (81.1%), mixed (11.3%), or sarcomatoid (3.8%). Treatment-related adverse events of any grade occurred in 41 patients (77.4%); the most common (>10%) were infusion-related reactions (20 [37.7%]), fatigue (8 [15.1%]), chills (8 [15.1%]), and pyrexia (6 [11.3%]), all grade 1/2. Four patients (7.5%) experienced treatment-related grade ≥3 adverse effects (colitis, decreased lymphocyte count, and increased GGT or blood creatine phosphokinase; each 1 event). No treatment-related adverse effects had a fatal outcome. Objective responses were observed in 9.4% pts (5 PRs; 95% CI 3.1, 20.7); all were partial responses. Stable disease (SD) was observed in 9 additional patients (45%). The overall disease control rate was 56.6% (30 patients).

Predicting PD-1/PD-L1 checkpoint inhibition:

PD-L1 has been implicated as a putative biomarker of efficacy for PD-L1 and PD-1 inhibitors from correlative analysis of several clinical studies [25]; however, the association between PD-L1 expression and efficacy remains controversial. Several trials have investigated the role of PD-1/PD-L1 inhibitors in MPM. Nivolumab in the Phase II single arm NivoMes trial (NCT02497508) and avelumab in the Phase I JAVELIN trial (NCT01772004) recruited patients regardless of PD-L1 expression. The Keynote 028 study failed to demonstrate a clear association between the level of PDL1-1 expression and efficacy of pembrolizumab in MPM, as did the positive study of nivolumab in squamous NSCLC (checkmate 017).

Keynote 028 showed a disease control rate (DCR) of 72% (n = 25) and median OS of 18 months, whereas patients treated with nivolumab in the NivoMes study exhibited a DCR of 50% (n = 34). A DCR of 60% was found in 20 patients with MPM treated with avelumab in the JAVELIN trial. To date, however, unlike in NSCLC, there is still no approval for PD-1/PD-L1 inhibitors in MPM.

Mutation burden is associated with increased neoantigen load and sensitivity to PD-1 blockade [16, 19, 20]. We and others have shown that a subset of mesotheliomas exhibit significant genomic instability and high mutational burden, with shorter survival [28]; however, neither the correlation with expression of PD-L1 or response to PD-L1/PD-1 inhibition has been explored.

In June 2017, an Australian group showed that in a cohort of 46 patients with mesothelioma, a subset of 29% of patients had high expression of PDL1 (>50% of tumour cells positive). This was associated with a very high response rate of 50% and a trend to improved progression free and overall survival. This data provides the most convincing evidence to date that a subset of PDL1 high expressing mesotheliomas, are the most responsive to pembrolizumab. The evidence is currently lacking for nivolumab,

In summary, PD-L1 is therefore emerging as a potentially useful biomarker for selection of immunotherapy where the likelihood of benefit could be assessed based on the level of expression, with <1% being less likely to benefit compared with >50% expression, where the benefit (based on early evidence) could be considerable.

Bevacizumab is active in mesothelioma and clinically potentiates Atezolizumab

Angiogenesis plays an important role in mesothelioma. Several studies have shown that mesothelioma cell lines produce large amounts of proangiogenic factors, including VEGF, express VEGFR-1 and VEGFR-2 and respond to VEGF through increasing proliferation. Blocking VEGFRs with a monoclonal antibody inhibits cell growth. Analysis of mesothelioma tumour samples from patients have shown increased expression of multiple pro-angiogenic cytokines including VEGF, associated with worse patient survival. Expression of VEGR receptors in patient mesothelioma samples varies between 20-70%.

Bevacizumab is a recombinant humanised monoclonal immunoglobulin antibody against VEGF-A ligand. The MAPs clinical trial has recently reported efficacy of bevacizumab in mesothelioma when combined with pemetrexed and cisplatin, versus cisplatin alone [29]. After 6 cycles of chemotherapy, bevacizumab maintenance every 3 weeks was continued in the combination arm until PD or unacceptable toxicity. Of the 448 patients enrolled into this study, overall survival increased from 16.1 months from the control arm versus 18.8 months for the bevacizumab arm (Hazard ratio 0.77).

A multi-arm open label phase 1b study (GP28328 study, NCT01633970) evaluated the safety and preliminary efficacy of atezolizumab with bevacizumab in multiple solid tumours types. A single dose of bevacizumab on C1D1 followed by combined administration of atezolizumab and bevacizumab every 3 weeks was administered. In the cohort of patients with metastatic

renal cell carcinoma (mRCC), tumour biopsies were collected before treatment, 15–18 days following bevacizumab and 4–6 weeks after initiation of atezolizumab and bevacizumab combination treatment (PMCID: PMC5013615). Partial responses were observed in 4 of 10 patients, with an additional 4 patients achieving prolonged stable disease In the Phase II study in mRCC 305 patients with mRCC were treated in a 1:1:1 ratio to receive standard-of-care sunitinib vs atezolizumab monotherapy vs atezolizumab plus bevacizumab. Crossover to the combination therapy arm was permitted at disease progression. Median progression-free survival was 6.1 months for atezolizumab alone, 8.4 months with sunitinib, and 11.7 months for the combination of atezolizumab plus bevacizumab.

A striking difference was observed in PD-L1–positive patients, with a 36% reduction in the risk of death or disease progression favouring the combination. In PD-L1–positive patients, median progression-free survival was 5.5 months for atezolizumab, 7.8 months for sunitinib, and **14.7 months for atezolizumab and bevacizumab**. The highest overall response rate was 46% in PD-L1–positive patients in the combination arm and 28% in this subgroup with atezolizumab alone https://meetinglibrary.asco.org/record/144688/abstract.

Proof of concept regarding combination VEGF inhibition and PDL1 inhibition has been reported in the IMPOWER150 randomised clinical trial (NCT02366143). In this study 1202 patients received atezolizumab 1200 mg + C AUC 6 + P 200 mg/m2 (Arm A) or atezolizumab + CP + bevacizumab 15 mg/kg (Arm B) vs CP + bevacizumab (Arm C) IV q3w for 4 or 6 cycles per investigator (INV); then maintenance atezolizumab, atezolizumab + bevacizumab, or bevacizumab, respectively. Co-primary endpoints were investigator-assessed PFS in the intention to treat wild type (EGFR/ALK WT) and in wild type patients with expression of a tumour T-effector gene signature (Teff-high WT) and OS in the ITT-WT. Data cut off: 1/22/2018. With 13.5 mo min follow up OS was improved in Arm B vs C (HR, 0.78 [95% CI: 0.64, 0.96]; P = 0.016) in the ITT-WT; Arm A vs C OS HR was 0.88 (95% CI: 0.72, 1.08; P = 0.204). IMpower150 therefore showed a significant OS benefit with atezolizumab + CP + bevacizumab vs CP + bevacizumab in 1L NSQ NSCLC, with no new safety signals seen.

Collectively, this data suggests that combined bevacizumab combination has the potential to potentiate P-DL1 inhibition by atezolizumab in mesothelioma. The aim of this study is therefore to determine the response rate and safety and efficacy of combined PD-L1 blockade/anti-angiogenesis.

2. OBJECTIVES

2.1 Primary Objective

To establish the 12 week disease control rate of atezolizumab and bevacizumab in patients with relapsed PD-L1 positive mesothelioma.

2.2 Secondary Objectives

To establish the safety, toxicity, objective response rate and 24 week disease control rate of atezolizumab and bevacizumab in patients with PD-L1 positive relapsed mesothelioma.

2.3 Exploratory Objectives

Tumour mutation burden will be assessed by genome-wide copy number analysis will be conducted to genotype responders to atezolizumab and bevacizumab and allow correlation of LOH, mutation burden and efficacy.

Further analysis of PD-L1 expression (1-49% versus > 50%) and its correlation with response will be assessed as an exploratory outcome. Response will also be correlated with prior response to first line platinum based therapy.

Immune regulated gene signature. Tumour samples will have RNA extracted, and gene expression analysis will be conducted to correlate the impact of tumour infiltrating lymphocytes on response.

To assess correlation between gut microbiome composition and response to therapy.

3. STUDY DESIGN

3.1 Summary of trial design

MiST4 is a single arm, open label phase IIa clinical trial. Approximately 26 patients with relapsed mesothelioma will be entered following informed consent to receive atezolizumab and bevacizumab. Based on the preliminary results from the Phase II study of atezolizumab and bevacizumab in mRCC with a reduction in the risk of death and improvement in progression free survival according to PDL1 status, enrolment will be restricted to patients who are PD-L1 positive (defined as >1% staining by IHC using the 22C3 PHARMDX PD-L1 antibody). There are limited data on the prevalence and the prognostic role of PD-L1 expression in MPM. Emerging data suggests that PDL1 expression is around 49% in mesothelioma (enriched for biphasic and sarcomatoid subtypes, where expression is almost 100%). High expression of PD-L1 is seen in around 29% of patients with mesothelioma (http://ascopubs.org/doi/abs/10.1200/JCO.2017.35.15_suppl.8514.).

In stage 1 (MiST Pre-screening), all patients consented to their stored archived tissue (FFPE) being accessed for molecular analysis and treatment stratification purposes, during stage 2 (treatment) patients will be asked to provide blood samples at baseline and at disease progression for research purposes. In addition, patients will be asked to provide another tissue sample (FFPE) via CT or Ultrasound (US) guided biopsy at disease progression **if they have had a prior response to atezolizumab and bevacizumab assessed by modified RECIST 1.1**; however, this procedure is optional.

In stage 2 (treatment), **atezolizumab** will be given as a fixed dose of 1200 milligrams (mg) via intravenous (IV) infusion on day 1 of a 21-day cycle every 21 and **Bevacizumab** will be given as 15 milligrams per kilogram (mg/kg) via IV infusion on Days 1 of a 21-day cycle.

Patients will receive atezolizumab and bevacizumab for 8 cycles (24 weeks) or until disease progression, loss of clinical benefit, symptomatic deterioration, unacceptable toxicity, patient withdrawal or death, whichever comes first.

Patients will be monitored by CT scan every 6 weeks for 24 weeks, when they will be managed according to local practice.

Patients who are benefiting from atezolizumab and bevacizumab beyond 24 weeks may be able to continue treatment on a "named" patient basis until disease progression, loss of clinical benefit, symptomatic deterioration, unacceptable toxicity, patient withdrawal or death, whichever comes first, this will be "requested as a bona fide 7 unsolicited order, formulated in accordance with the specifications of an authorised healthcare professional and for use by an individual patient under his or her direct personal responsibility as a special needs request." This is described in Guidance Note 14 on the MHRA website:

https://assets.publishing.service.gov.uk/government/uploads/system/uploads/attachment_data/file/373505/The_supply_of_unlicensed_medicinal_products__specials_.pdf

Patients who discontinue either atezolizumab or bevacizumab may be able to continue on single agent therapy until disease progression or loss of clinical bene fit, whichever comes first. Guidelines for dosage modification, treatment interruption or discontinuation and the management of specific adverse events are provided in section 12.

Follow-up will be at 30 days and 6 months after the participant's last dose of atezolizumab and/or bevacizumab, up until death or patient withdrawal (whichever comes first). Patients for whom there is no date of death will be traced via an NHS information centre.

Patients who discontinue for loss of clinical benefit, unacceptable toxicity or symptomatic deterioration may be considered for eligibility for an alternative MiST trial arm. However they must meet the eligibility criteria specific to the study arm.

3.2 Flow chart of trial design

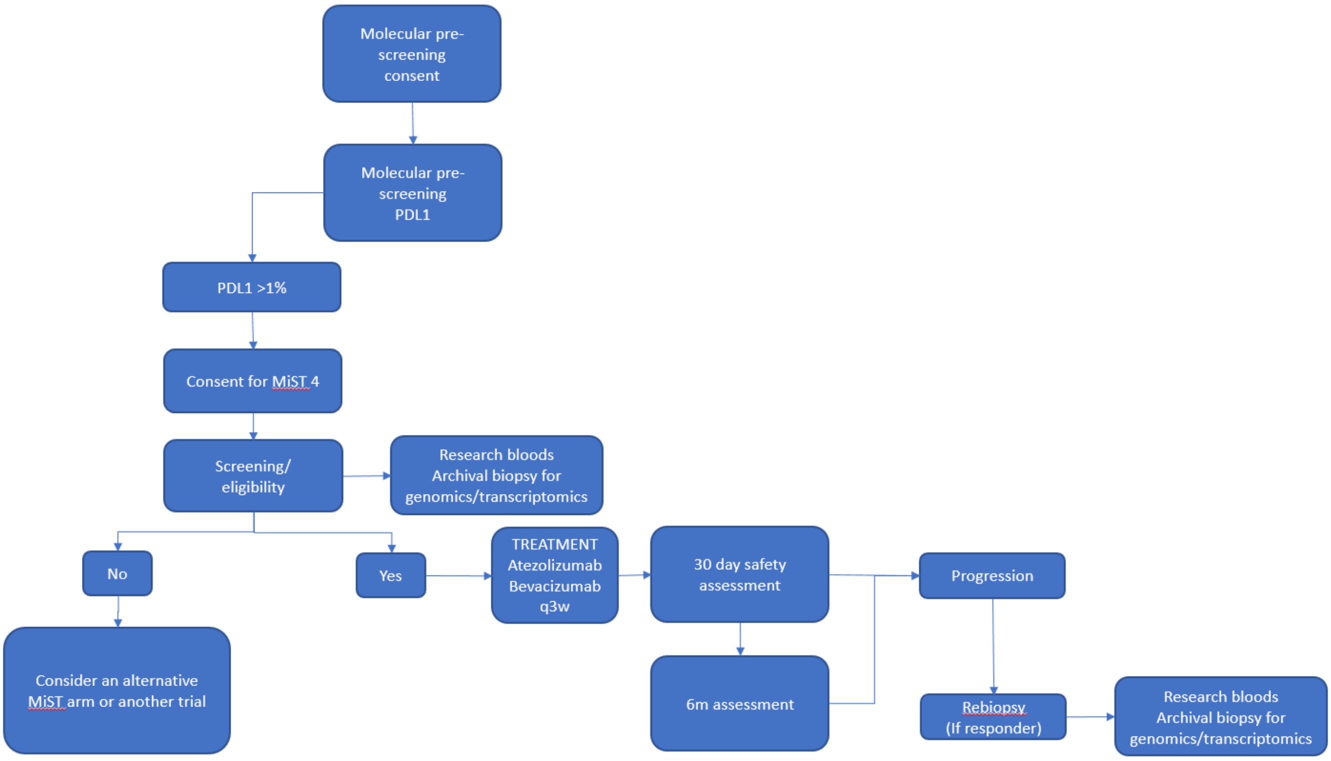

3.3 Primary Endpoint/Outcome Measures

3.3.1 Primary Endpoint/Outcome Measure

- To establish the 12 week disease control rate of atezolizumab and bevacizumab as assessed by modified RECIST 1.1, in patients with relapsed mesothelioma with tumours that are PD-L1 positive (defined as >1% IHC using Dako clone 22C3).

3.3.2 Secondary Endpoints/Outcome Measures

- To establish the safety and toxicity of atezolizumab and bevacizumab in patients with relapsed mesothelioma.
- To establish the 24 week disease control rate by modified RECIST 1.1 in patients receiving atezolizumab and bevacizumab in patients with relapsed mesothelioma.
- To establish objective response rate of atezolizumab and bevacizumab in patients with relapsed mesothelioma within 24 weeks.

4. TRIAL PARTICIPANTS

4.1 Overall description of trial Patients

Patients with clinically confirmed, relapsed mesothelioma will be approached to take part in this study. This is an open label clinical trial, in which up to 26 eligible patients will be treated with atezolizumab and bevacizumab.

4.2 Inclusion Criteria

Patients meeting the following criteria may be included in to the study:

1. Confirmed histological diagnosis of malignant mesothelioma (pleural or peritoneal) with any histological subtype.
2. Subjects must be positive/proficient in PDL1 expression confirmed via IHC testing, performed by the central lab.
3. Patients must have received prior treatment with first-line standard platinum doublet based chemotherapy (**PLEASE NOTE:** patients that have received prior treatment with platinum+pemetrexed and bevacizumab as a combination therapy are **NOT** permitted). Re-challenge with first-line chemotherapy is allowed. There is no restriction on the line of therapy.
4. Evidence of disease progression according to modified RECIST 1.1 reporting of CT scan up to a maximum of 28 days prior to treatment
5. Life expectancy ≥ 12 weeks
6. ECOG performance status 0-1
7. Men or women aged 18 years or over
8. Willing to consent and able to undergo required procedures to provide blood and tissue for translational research
9. Disease which is measurable using modified RECIST 1.1
10. Adequate haematologic and end-organ function, defined by the following laboratory test results, obtained within 14 days prior to initiation of study treatment:
    - Absolute neutrophil count ≥ 1.5 × 10^9/L (1500/μL) without granulocyte colony-stimulating factor support
    - Lymphocyte count ≥ 0.5 × 10^9/L (500/μL)
    - Platelet count ≥ 100 × 10^9/L (100,000/μL) without transfusion
    - Haemoglobin ≥ 90 g/L (9g/dL)

    Patients may be transfused to meet this criterion.
    - AST, ALT, and alkaline phosphatase (ALP) ≤ 2.5 × upper limit of normal (ULN), with the following exceptions:
        - Patients with documented liver metastases: AST and ALT ≤ 5 × ULN
        - Patients with documented liver or bone metastases: ALP ≤ 5 × ULN
    - Serum bilirubin ≤ 1.5 × ULN with the following exception:

Patients with known Gilbert disease: serum bilirubin level $\leq 3 \times$ ULN

- {Serum creatinine $\leq 1.5 \times$ ULN} **[or]** {Creatinine clearance > 45ml/min mL/min (calculated using the Cockcroft-Gault formula)}
- Serum albumin $\geq$ 25 g/L (2.5 g/dL)
- For patients not receiving therapeutic anticoagulation: INR or aPTT $\leq 1.5 \times$ ULN
- For patients receiving therapeutic anticoagulation: stable anticoagulant regimen

11. **For women** who are not postmenopausal (≥12 months of non-therapy-induced amenorrhea) or surgically sterile (absence of ovaries and/or uterus): agreement to remain abstinent (refrain from heterosexual intercourse) or use single or combined contraception methods that result in a failure rate of <1% per year during the treatment period and for at least 6 months after the last dose of atezolizumab and bevacizumab. Abstinence is only acceptable if it is in line with the preferred and usual lifestyle of the patient. Periodic abstinence (e.g., calendar, ovulation, symptothermal, or postovulation methods) and withdrawal are not acceptable methods of contraception. Examples of contraceptive methods with a failure rate of <1% per year include tubal ligation, male sterilization, hormonal implants, established, proper use of combined oral or injected hormonal contraceptives, and certain intrauterine devices.

For men, agreement to remain abstinent or use a condom during the treatment period and for at least 90 days after the last dose of atezolizumab or bevacizumab and agreement to refrain from donating sperm during this same period. Men with a pregnant partner must agree to remain abstinent or use a condom for the duration of the pregnancy. Abstinence is only acceptable if it is in line with the preferred and usual lifestyle of the patient. Periodic abstinence (e.g., calendar, ovulation, symptothermal, or postovulation methods) and withdrawal are not acceptable methods of contraception.

12. Patients must provide informed consent before any study specific procedures. The PI must confirm the eligibility of a patient in the patient's medical notes before enrolment.

4.3 Exclusion Criteria

Disease specific exclusions and general medical exclusions:

1. History of malignancy within 5 years prior to screening, with the exception of malignancies with a negligible risk of metastasis or death (e.g., 5-year OS rate > 90%), such as adequately treated carcinoma in situ of the cervix, non-melanoma skin carcinoma, localized prostate cancer, ductal carcinoma in situ, or Stage I uterine cancer.
2. Patient's negative/deficient in PDL1 expression (results show <u><</u>1% PDL1 expression) confirmed via IHC testing, performed by the central lab.
3. Positive pregnancy test for females and male or female patients refusing to adhere to use highly effective method of contraception or to practice true abstinence defined in accordance with the Clinical Trials Facilitation Group as in the context of this guidance sexual abstinence is considered a highly effective method only if defined as refraining from heterosexual intercourse during the entire period of risk associated with the study treatments. The reliability of sexual abstinence needs to be evaluated in relation to the duration of the clinical trial and the preferred and usual lifestyle of the subject.
4. Symptomatic, untreated, or actively progressing central nervous system (CNS) metastases. Asymptomatic patients with treated CNS lesions are eligible, provided that all of the following criteria are met:
    - Measurable disease, per RECIST v1.1, must be present outside the CNS.
    - The patient has no history of intracranial haemorrhage or spinal cord haemorrhage.
    - The patient has not undergone stereotactic radiotherapy within 7 days prior to initiation of study treatment, whole-brain radiotherapy within 14 days prior to initiation of study treatment, or neurosurgical resection within 28 days prior to initiation of study treatment.
    - The patient has no on-going requirement for corticosteroids as therapy for CNS disease. Anticonvulsant therapy at a stable dose is permitted.

5. Palliative radiotherapy within the RECIST 1.1 area in the 4 weeks prior to baseline CT scan.
6. Significant cardiovascular disease (such as New York Heart Association Class II or greater cardiac disease, myocardial infarction, or cerebrovascular accident) within 3 months prior to initiation of study treatment, unstable arrhythmia, or unstable angina.
7. Patients with severe hepatic insufficiency or severe renal impairment.
8. Patients requiring long term oxygen therapy.
9. Any other significant disease or disorder which, in the opinion of the Investigator, may either put the participants at risk because of participation in the study, or may influence the result of the study, or the participant's ability to participate in the study.
10. All toxicities attributed to prior anti-cancer therapy not resolved to grade 1 or less before administration of study drug.
11. Treatment with a live, attenuated vaccine within 4 weeks prior to initiation of study treatment, or anticipation of need for such a vaccine during atezolizumab treatment or within 5 months after the final dose of atezolizumab.
12. Patients, who have received prior treatment with chemotherapy or an investigational agent within 14 days of receiving the first dose of study treatment.
13. Patients, who have received prior treatment with either an anti-VEGF or anti-PDL1 inhibitor.
14. History of leptomeningeal disease.
15. Uncontrolled tumour-related pain. Patients requiring pain medication must be on a stable regimen at study entry.
16. Symptomatic lesions (e.g., bone metastases or metastases causing nerve impingement) amenable to palliative radiotherapy should be treated prior to enrolment. Patients should be recovered from the effects of radiation. There is no required minimum recovery period. Asymptomatic metastatic lesions that would likely cause functional deficits or intractable pain with further growth (e.g., epidural metastasis that is not currently associated with spinal cord compression) should be considered for loco-regional therapy if appropriate prior to enrolment.
17. Uncontrolled pleural effusion, pericardial effusion, or ascites requiring recurrent drainage procedures (once monthly or more frequently). Patients with indwelling catheters (e.g., PleurX®) are allowed.

18. Uncontrolled or symptomatic hypercalcemia (ionized calcium > 1.5 mmol/L, calcium > 12 mg/dL or corrected serum calcium > ULN).
19. History of idiopathic pulmonary fibrosis, organizing pneumonia (e.g., bronchiolitis obliterans), drug-induced pneumonitis, or idiopathic pneumonitis, or evidence of active pneumonitis on screening chest computed tomography (CT) scan. History of radiation pneumonitis in the radiation field (fibrosis) is permitted.
20. Active tuberculosis.
21. Major surgical procedure, other than for diagnosis, within 4 weeks prior to initiation of study treatment, or anticipation of need for a major surgical procedure during the study.
22. Severe infection within 4 weeks prior to initiation of study treatment, including, but not limited to, hospitalization for complications of infection, bacteremia, or severe pneumonia.
23. Treatment with therapeutic oral or IV antibiotics within 2 weeks prior to initiation of study treatment.
24. Patients receiving prophylactic antibiotics (e.g., to prevent a urinary tract infection or chronic obstructive pulmonary disease exacerbation) are eligible for the study.
25. Prior allogeneic stem cell or solid organ transplantation.
26. Any other disease, metabolic dysfunction, physical examination finding, or clinical laboratory finding that contraindicates the use of an investigational drug, may affect the interpretation of the results, or may render the patient at high risk from treatment complications.
27. Breastfeeding
28. Hypersensitivity to the active substances or to any of their excipients

Treatment Specific Exclusion Criteria:

Prior treatments

1. CD137 agonists, anti−CTLA-4, anti−PD-1, or anti−PD-L1 therapeutic antibody or pathway-targeting agents.
2. Treatment with systemic immunostimulatory agents (including but not limited to IFN α, IL-2) for the treatment of non-malignant conditions within 6 weeks or five half-lives of the drug, whichever is shorter, prior to Cycle 1, Day 1.
3. Treatment with systemic immunosuppressive medications (including but not limited to prednisone, dexamethasone cyclophosphamide, azathioprine, methotrexate, thalidomide, and anti-tumour necrosis factor [anti−TNF] agents) within 2 weeks prior to Cycle 1, Day 1. Patients who have received acute, low-dose, systemic immunosuppressant medications (e.g., a one-time dose of dexamethasone for nausea) or physiologic replacement doses (i.e., prednisone 5−7.5 mg/day) for adrenal insufficiency may be enrolled in the study. The use of inhaled corticosteroids, physiologic replacement doses of glucocorticoids (i.e., for adrenal insufficiency), and mineralocorticoids (e.g., fludrocortisone) is allowed.

Hypersensitivity

4. History of severe allergic, anaphylactic, or other hypersensitivity reactions to chimeric or humanized antibodies or fusion proteins.
5. Known hypersensitivity or allergy to biopharmaceuticals produced in Chinese hamster ovary cells or any component of the atezolizumab or bevacizumab formulation.

Autoimmune related disease

6. Including but not limited to myasthenia gravis, myositis, autoimmune hepatitis, systemic lupus erythematosus, rheumatoid arthritis, inflammatory bowel disease, vascular thrombosis associated with antiphospholipid syndrome, Wegener's granulomatosis, Sjögren's syndrome, Guillain-Barré syndrome, multiple sclerosis, vasculitis, or glomerulonephritis. Patients with a history of autoimmune-related hypothyroidism on a stable dose of thyroid replacement hormone are eligible for this study. Patients with controlled Type I diabetes mellitus on a stable dose of insulin regimen may be eligible for this study.

7. Patients with eczema, psoriasis, lichen simplex chronicus, or vitiligo with dermatologic manifestations only (e.g., patients with psoriatic arthritis are excluded) are eligible for the study provided all of following conditions are met:
    - Rash must cover < 10% of body surface area.
    - Disease is well controlled at baseline and requires only low-potency topical corticosteroids.
    - No occurrence of acute exacerbations of the underlying condition requiring psoralen plus ultraviolet A radiation, methotrexate, retinoids, biologic agents, oral calcineurin inhibitors, or high potency or oral corticosteroids within the previous 12 months.

Infection

8. Patients with active bacterial infection (requiring intravenous [IV] antibiotics at time of initiating study treatment), fungal infection. Screening is not required for enrolment.
9. Known history of testing positive for human immunodeficiency virus (HIV) or known acquired immunodeficiency syndrome (AIDS).
10. Any positive test for hepatitis B virus or hepatitis C virus indicating acute or chronic infection.

Cardiovascular

11. Inadequately controlled hypertension (defined as systolic blood pressure > 150 mmHg and/or diastolic blood pressure > 100 mmHg). Anti-hypertensive therapy to maintain a systolic blood pressure <150mmHg and/or diastolic blood pressure <100mmHg is permitted.
12. Prior history of hypertensive crisis or hypertensive encephalopathy.
13. History of stroke or transient ischemic attack within 6 months prior to Cycle 1, Day 1.
14. Significant vascular disease (e.g., aortic aneurysm requiring surgical repair or recent peripheral arterial thrombosis) within 6 months prior to Cycle 1, Day 1.
15. Patients with a baseline ECG demonstrating a QTc >460 ms.

16. Evidence of bleeding diathesis or clinically significant coagulopathy (in the absence of therapeutic anticoagulation).
17. Current or recent (within 10 calendar days prior to Cycle 1, Day 1) use of dipyramidole, ticlopidine, clopidogrel, or cilostazol. Prophylactic or therapeutic use of low molecular−weight heparin (e.g., enoxaparin), direct thrombin inhibitors, or warfarin are permitted, provided, where appropriate anticoagulation indices are stable. Patients should have been on a stable dose (for therapeutic use) for at least two weeks (or until reaching steady state level of the drug) prior to the first study treatment.

Bevacizumab Specific Restrictions:

18. Core biopsy or other minor surgical procedure, excluding placement of a vascular access device, within 7 calendar days prior to the first dose of bevacizumab.
19. History of abdominal or tracheoesophageal fistula or gastrointestinal perforation within 6 months prior to Cycle 1, Day 1.
20. Clinical signs or symptoms of gastrointestinal obstruction or requirement for routine parenteral hydration, parenteral nutrition, or tube feeding.
21. Evidence of abdominal free air not explained by paracentesis or recent surgical procedure.
22. Serious, non-healing or dehiscing wound, active ulcer, or untreated bone fracture
23. Proteinuria, as demonstrated by urine dipstick or > 1.0 g of protein in a 24-hour urine collection.
24. All patients with ≥2+ protein on dipstick urinalysis at baseline must undergo a 24-hour urine collection for protein.

5. STUDY PROCEDURES

5.1 Informed Consent

All patients with relapsed mesothelioma will be given the Patient Information Sheet about this study during their routine clinic visits by a delegated member of the research team. The participant must personally sign and date the latest approved version of the informed consent form before any study specific procedures are performed. Written and verbal versions of the participant information and informed consent will be presented to the participants detailing no less than: the exact nature of the study; the implications and constraints of the protocol; the known side effects and any risks involved in taking part. It will be clearly stated that the participant is free to withdraw from the study at any time for any reason without prejudice to future care, and with no obligation to give the reason for withdrawal.

The participant will be given a minimum of 24 hours (but as much time as wished) to consider the information, and given the opportunity to question the Investigator, their GP or other independent parties to decide whether they will participate in the study. Written Informed Consent will then be obtained by means of participant dated signature and dated signature of the person who presented and obtained the informed consent. The person who obtained the consent must be suitably qualified and experienced, and have been authorised to do so by the Chief/Principal Investigator as detailed on the Delegation of Authority and Signature log for the study. The original signed form will be retained at the study site within the Investigator Site File (ISF). A copy of the signed Informed Consent form will be given to participants and a copy retained in the participants medical notes.

5.2 Screening and Eligibility Assessment

Any queries about whether a patient is eligible to enter the trial should be discussed with the CI/PI before study entry. Any issues or concerns should be raised with the CI or one of the clinical Co-Investigators in the CI's absence. Protocol waivers are not allowed. Patients are eligible for the trial if all the inclusion criteria (Section 4.2) are met and none of the exclusion criteria (Section 4.3) apply.

6. TRIAL ASSESSMENTS

Once participants have provided written informed consent, they are only required initially to attend one study visit, in order to conduct baseline investigations and confirm their eligibility into the study. This visit will take place by appointment soon after written informed consent has been obtained. This visit can adhere to the normal patient care pathway.

Prior to baseline assessment, any potential patient should be entered onto the MiST4 screening log and filed in the ISF.

The following procedures must be performed **after consent** in order to confirm patient eligibility:

6.1 Baseline Assessments

- Inclusion/exclusion and trial entry verification (including archival tissue based molecular stratification). If no archival tissue is available a screening biopsy will be required to enable patient participation
- Medical history
- Physical examination
- Vital signs (height, weight, temperature, blood pressure, heart rate and respiratory frequency)
- CT assessment (thorax/abdo +/- pelvis) of measurable disease by mRECIST1.1. CT scan should be performed within 28 days. If a routine diagnostic scan has been performed prior to consent and within 28 days of C1D1 then repeat imaging is not required.
- ECOG performance status
- Haematological function including:
  - Full Blood Count (FBC) (no more than 72 hours before treatment) including:
    - Haemoglobin
    - Lymphocyte count
    - White blood count
    - Neutrophil count

        - Platelet count
    - Biochemistry Profile (no more than 72 hours before treatment) including:
        - Serum bilirubin
        - ALP
        - AST and ALT
        - Corrected calcium
        - Serum creatinine/creatinine clearance
        - Potassium
        - Sodium
        - Albumin
        - Urea
        - INR
        - APPT
        - C-reactive protein
        - LDH
        - Urinalysis (specific gravity, pH, glucose, protein, ketones, and blood)
        - Thyroid function testing (thyroid stimulating hormone [TSH], free T3, free T4)
- Concomitant medication record
- Serum pregnancy test
- Research blood sample
- Stool collection/microbiome

Once the study visit is complete and the investigations have confirmed eligibility, study related activity will be focused on commencing treatment.

6.2 Assessments on Treatment

The following procedures must be performed prior to each treatment cycle:

- Physical examination

- Vital signs (weight, temperature, blood pressure, heart rate and respiratory frequency)

Please note: Vital signs are required pre and post dose of each administration of atezolizumab.

- ECG (at day 1 of each cycle **only** if clinically indicated)

- CT scan carried out **every six weeks** with full clinical evaluation to evaluate disease response by modified RECIST 1.1. CT scans can be conducted +/- 7 days of the scheduled scan date.

- Haematological function (no more than 72 hours prior to treatment) consisting of:

o Full Blood Count (FBC): Required the day of, or up to 72 hours before day 1 of each treatment cycle. Investigations also to be conducted on **day 15** of the first and second cycle only. Haemoglobin ≥ 90 g/l, white blood count ≥ 3 x 10^9/l, neutrophil count ≥ 1.5 x 10^9/l, Lymphocyte count ≥ 0.5 × 10^9/L (500/μL), Platelet count ≥100 x 10^9/L, will be required for retreatment.

  - Haemoglobin
  - White blood count
  - Lymphocyte count
  - Neutrophil count
  - Platelet count

o Biochemistry Profile: Required the day of, or up to 72 hours before day 1 of each treatment cycle. Investigations also to be conducted on **day 15** of the first and second cycle only.

  - Bilirubin
  - ALP
  - AST or ALT
  - Corrected calcium
  - Serum creatinine/creatinine clearance
  - Potassium

- Sodium
- Albumin
- Urea
- Urinalysis (specific gravity, pH, glucose, protein, ketones, and blood)
- Thyroid function testing (thyroid stimulating hormone [TSH], free T3, free T4)

- Pregnancy test (urine or serum)
- Concomitant medication record
- ECOG performance status
- Evaluation of toxicities using NCI CTCAE (v4.03)

If treatment is delayed for an adverse event, assessments should continue to be performed while treatment is on hold. All visits must occur within ± 3 days from the scheduled date unless otherwise noted. All assessments will be performed on the day of the specified visit unless a time window is specified. Assessments scheduled on the day of study treatment administration (Day 1) of each cycle should be performed prior to study treatment infusion unless otherwise noted. Local laboratory assessments from each cycle must be reviewed prior to study treatment administration for each cycle.

The following assessments may be performed ≤ 3 days before Day 1 of each cycle:

- ECOG performance status
- Physical examination
- Local laboratory tests.

If scheduled dosing and study assessments are precluded because of a holiday, weekend, or other event, then dosing may be postponed to the soonest following date, with subsequent dosing continuing on a regular dosing schedule. If treatment was postponed for fewer than 2 days, the patient can resume the original schedule.

After two complete cycles, treatment can be delayed by 1 week to allow for vacations.

6.3 End of Treatment (Cycle 8 – Day 21).

- Vital signs (weight, temperature, blood pressure, heart rate and respiratory frequency)
- ECOG performance status
- CT scan carried out with full clinical evaluation to evaluate disease response by modified RECIST 1.1.
- Evaluation of toxicities using NCI CTCAE (v4.03)

6.4 Assessments at Disease Progression

- Haematological function consisting of:
  - Full Blood Count (FBC) including:-
    - Haemoglobin
    - White blood count
    - Lymphocyte count
    - Neutrophil count
    - Platelet count
  - Biochemistry Profile including:
    - Serum bilirubin
    - ALP
    - AST or ALT
    - Corrected calcium
    - Serum creatinine/creatinine clearance
    - Potassium
    - Sodium
    - Albumin
    - Urea
    - INR
    - APPT
    - Urinalysis (specific gravity, pH, glucose, protein, ketones, and blood)

        - Thyroid function testing (thyroid stimulating hormone [TSH], free T3, free T4)

- Concomitant medication record
- ECOG performance status
- CT scan with modified RECIST 1.1 reporting
- Evaluation of toxicities using NCI CTCAE (v4.03)
- Research blood
- Optional tissue biopsy

6.5 Follow Up 30 Days Post Progression/withdrawal OR End of 8 Cycles of Treatment

- Physical examination
- Vital signs (weight, temperature, blood pressure, heart rate and respiratory frequency)
- Evaluation of toxicities using NCI CTCAE (v4.03)
- Concomitant medication record Survival status
- Haematological function consisting of:
    - Full Blood Count (FBC) including:-
        - Haemoglobin
        - White blood count
        - Lymphocyte count
        - Neutrophil count
        - Platelet count
    - Biochemistry Profile including:
        - Serum bilirubin
        - ALP
        - AST or ALT
        - Corrected calcium
        - Serum creatinine/creatinine clearance

- Potassium
- Sodium
- Albumin
- Urea
- INR
- APPT
- Urinalysis (specific gravity, pH, glucose, protein, ketones, and blood)
- Thyroid function testing (thyroid stimulating hormone [TSH], free T3, free T4)
- Pregnancy test (urine or serum)
- Survival status
- Treatment outcome assessment

6.6 Follow-up Assessment 6 Months after Disease Progression/End of treatment

- Physical examination
- Vital signs (weight, temperature, blood pressure, heart rate and respiratory frequency)
- Survival status
- Evaluation of toxicities

7. COMPLETION OF CRFS

The CRF should be completed within 7 days of the visit. According to the principles of GCP, the PI is responsible for ensuring accuracy, completeness, legibility and timeliness of the data reported in the CRFs. CRF data received by the MiST Programme Team for this study will be checked for missing, illegible or unusual values (range checks) and consistency over time. If missing or questionable data are identified, a data query will be raised by the MiST Programme Team on a data clarification form.

The data clarification form will be sent to the relevant research team. The research team will be required to answer the data query or correct data via the data clarification form. The case report form pages should not be altered. All answered data queries and corrections should be signed off and dated by a delegated member of the research team. The completed data clarification form should be returned to the MiST Programme Team and a copy retained at the site along with the participants' CRFs. The MiST Programme Team will send reminders for any overdue data. It is the site's responsibility to submit complete and accurate data in timely manner.

8. DEFINITION OF END OF TRIAL

Patients will be treated until completion of 24 weeks (8 cycles), treatment withdrawal, disease progression or death (whichever comes first).

The end of trial will be 6 months after the last data capture. This will allow sufficient time for the completion of protocol procedures, data collection and data input.

9. DISCONTINUATION/WITHDRAWAL OF PARTICIPANTS FROM TRIAL TREATMENT

In consenting to the trial, participants are consenting to trial treatment, trial follow-up and data collection. Each participant has the right to withdraw from the trial at any time without giving reason. There can be different levels of participant withdrawal from the trial including:

Level 1: Discontinue from receiving trial treatment
Patients will discontinue the trial treatment but are happy to continue providing us with information at follow-up visits and (in some cases) blood/tissue samples.

OR

Level 2: Withdraw consent from the translational study
Patients will remain on trial treatment and follow-up but won't give any extra blood/tissue samples above what is routine practice.

OR

Level 3: Withdraw consent from the translational study and discontinue trial treatment
Patients will discontinue the trial treatment and won't give blood/tissue samples above what is routine practice, but they are happy to continue providing us with information at follow-up visits.

OR

Level 4: Complete withdrawal of consent from the trial
Patients will discontinue the trial treatment and won't come back for extra follow-up visits nor provide any blood/tissue samples above what is routine practice.

If a participant wishes to withdraw from trial treatment, participating sites should nevertheless explain the importance of remaining on trial follow up for the purposes of data capture only. Withdrawal for any reason requires a completed withdrawal CRF and a hard

copy to be sent to the MiST Programme Team. Participants do not have to give a reason for their withdrawal but research staff should make a reasonable attempt to find out why.

In addition, the investigator may discontinue a participant from the study at any time if the investigator considers it necessary for any reason including:

- Intolerance to treatment (including SAEs and toxicities)
- Clinician's decision
- Non-compliance with protocol treatment
- Pregnancy
- Loss of Capacity

10. LOST TO FOLLOW-UP

If a participant is lost to follow up the MiST Programme Team will request that the PI/research team contacts the participant's GP or other central UK NHS bodies to obtain information on the participant's status. This will enable the research team to trace the participants cause and date of death. Patients will be asked to consent at registration for access to this information.

11. TREATMENT OF TRIAL PARTICIPANTS

11.1 Description of Atezolizumab

Atezolizumab is supplied by Roche/Genentech. The atezolizumab drug product is provided in a single-use, 20-mL USP/Ph. Eur. Type 1 glass vial as a colorless-to-slightly-yellow, sterile, preservative-free clear liquid solution intended for IV administration. The vial is designed to deliver 20 mL (1200 mg) of atezolizumab solution, but may contain more than the stated volume to enable delivery of the entire 20 mL volume. The atezolizumab drug product is formulated as 60 mg/mL atezolizumab in 20 mM histidine acetate, 120 mM sucrose, 0.04% polysorbate 20, pH 5.8 (Phase III formulation).

Atezolizumab must be refrigerated at 2°C−8°C (36°F−46°F) upon receipt until use.

Atezolizumab vials should not be used beyond the expiration date provided by the manufacturer. No preservative is used in the atezolizumab drug product; therefore, each vial is intended for single use only. Vial contents should not be frozen or shaken and should be protected from direct sunlight.

For detailed instructions, see the Atezolizumab Pharmacy Manual and IB.

11.1.1 Preparation and Administration of Atezolizumab

The dose level of atezolizumab is 1200 mg (equivalent to an average body weight based dose of 15 mg/kg) administered by IV infusion Day1 of a 21 day cycle (±3 days).

Administration of atezolizumab should be performed in a setting with emergency medical facilities and staff who are trained to monitor for and respond to medical emergencies.

The initial dose of atezolizumab should be delivered over 60 (±15) minutes. If the first infusion is tolerated without infusion-associated adverse events, the second infusion may be delivered over 30 (± 10) minutes. If the 30-minute infusion is well tolerated, all subsequent infusions may be delivered over 30 (± 10) minutes. At all infusions, the patient's vital signs (heart rate, blood pressures, and temperature) should be determined before and after the infusion.

No pre-medication is allowed for the first dose of atezolizumab. Pre-medication may be administered for Cycles ≥ 2 at the discretion of the treating physician. The management of infusion-related reactions will be according to severity as follows:

- In the event that a patient experiences a mild (NCI CTCAE Grade 1) infusion-related event, the infusion rate should be reduced to half the rate being given at the time of event onset. Once the event has resolved, the investigator should continue to deliver the infusion at the reduced rate for 30 minutes. If tolerated, the infusion rate may then be increased to the original rate.
- In the event that a patient experiences a moderate infusion-related event (NCI CTCAE Grade 2) or flushing, fever, or throat pain, the patient should have his or her infusion immediately interrupted and should receive aggressive symptomatic treatment. The infusion should be restarted only after the symptoms have adequately resolved to the baseline grade. The infusion rate at restart should be half of the rate that was in progress at the time of the onset of the infusion-related event.
- For severe or life-threatening infusion-related events (NCI CTCAE Grade 3 or 4), the infusion should be stopped immediately, and aggressive resuscitation and supportive measures should be initiated. Patients experiencing severe or life-threatening infusion-related events will not receive further infusion and will be further managed as clinically indicated until the event resolves.

11.2 Description of Bevacizumab

Bevacizumab is supplied by Roche/Genentech as a clear-to-slightly-opalescent, sterile liquid ready for parenteral administration. Each 400-mg (25-mg/mL) glass vial contains 16 mL of bevacizumab (25 mg/mL) and each 100mg (25-mg/mL) glass vial contains 4 mL of bevacizumab (25 mg/mL) with a vehicle consisting of sodium phosphate, trehalose, polysorbate 20, and Sterile Water for Injection, USP. Vials contain no preservative and are for single use only. For further details, see the current bevacizumab Investigator Brochure.

Bevacizumab is intended for use solely in clinical trials. The drug provided for clinical trial use is expected to be very similar in safety and activity to the commercially marketed drug (Avastin®).

11.2.1 Preparation and Administration of Bevacizumab

The dose of bevacizumab in this study is 15 mg/kg administered by IV infusion every 3 weeks on Days 1 of each 21-day cycle.. The bevacizumab dose will be based on the patient's weight

at baseline (Cycle 1, Day 1) and will remain the same throughout the study unless there is a weight change of > 10% from baseline. Doses can be banded to the nearest measurable dose.

The initial dose of bevacizumab will be delivered over 90 (± 15) minutes. If the first infusion is tolerated without infusion-associated adverse events (fever and/or chills), the second infusion may be delivered over 60 (± 10) minutes. If the 60-minute infusion is well tolerated, all subsequent infusions may be delivered over 30 (± 10) minutes.

The patient should be observed for at least 2 hours after the first administration of the combination and for at least 1 hour for subsequent infusions.

If a patient experiences an infusion-associated adverse event, he or she may be pre-medicated for the next bevacizumab infusion; however, the infusion time may not be decreased for the subsequent infusion. If the next infusion is well tolerated with pre-medication, the subsequent infusion time may then be decreased by 30 minutes as long as the patient continues to be pre-medicated. If a patient experiences a second episode of an infusion-associated adverse event with the 60-minute infusion, all subsequent doses should be given over 90 (± 15) minutes. Similarly, if a patient experiences a second episode of an infusion-associated adverse event with the 30-minute infusion, all subsequent doses should be given over 60 (± 10) minutes.

Upon receipt of the bevacizumab, vials are to be refrigerated at 2−8°C (36−46°F) and should remain refrigerated until use. Vials should be protected from light. DO NOT FREEZE. DO NOT SHAKE. VIALS ARE FOR SINGLE USE ONLY. Vials used for one patient may not be used for any other patient.

11.3 Criteria for re-treatment with both agents:

Specific criteria apply to each IMP. Unless otherwise specified in toxicity management guidelines for each drug (see Section 12), a new cycle of treatment with atezolizumab and bevacizumab may begin if:

- ANC ≥ 1.0 x 10^9/L;
- Platelet count ≥ 100 x 10^9/L;

- Non-haematologic toxicities have returned to baseline or ≤ Grade 1 severity (or, at the investigator's discretion, ≤ Grade 2 severity if not considered a safety risk for the patient).

12. DOSE MODIFICATIONS AND DELAYS

Toxicities should be managed as described below according to grade and attribution of the toxicity. This section provides guidance on the monitoring and management of expected or potential toxicities associated with atezolizumab and bevacizumab.

12.1 Atezolizumab

12.1.1 Atezolizumab dose modifications:

There are no dose modifications or reductions permitted for atezolizumab.

Patients may temporarily suspend study drug if they experience toxicity that is considered to be related to study drug. If atezolizumab is held because of related adverse events for >42 day beyond when the next dose would have been given, then the patient will be discontinued from atezolizumab. If, in the judgement of the investigator, the patient is likely to derive clinical benefit from resuming atezolizumab after a hold of >42 days, the study drug may be restarted with the approval of the Chief Investigator.

If patients must be tapered off steroids, used to treat adverse events, study treatment may be held for >42 days until steroids are discontinued or reduced to a prednisolone dose (or dose equivalent) $\leq$ 10mg/day. The acceptable length of interruption must be agreed with the Chief Investigator.

Dose interruptions for reasons other than adverse events, such as surgical procedures may be allowed with Chief Investigator approval. The acceptable length of interruption must be agreed with the Chief Investigator. Patients who discontinue atezolizumab either transiently or permanently may continue on bevacizumab until disease progression if there is felt to be clinical benefit.

12.1.2 Management of Atezolizumab specific adverse events

Toxicities associated or possibly associated with atezolizumab treatment should be managed according to standard medical practice.

Current management of immune-related pneumonitis, hepatitis, colitis, endocrinopathies, meningoencephalitis, neuropathies, pancreatitis, myocarditis and infusion related reactions are described in the following link:

https://www.medicines.org.uk/emc/product/8442/rmms

Management of ocular, dermatological, renal overdose and systemic immune activation (SIA) are described below.

12.1.3 Ocular Events

An ophthalmologist should evaluate visual complaints (e.g., uveitis, retinal events). Management guidelines for ocular events are provided below.

Table 1: Management of atezolizumab associated ocular events

**Severity**	**Management**
Ocular event, Grade 1	• Continue atezolizumab.<br>• Patient referral to ophthalmologist is strongly recommended.<br>• Initiate treatment with topical corticosteroid eye drops and topical immunosuppressive therapy.<br>• If symptoms persist, treat as a Grade 2 event.
Ocular event, Grade 2	• Withhold atezolizumab for up to 12 weeks after event onset. [a]<br>• Patient referral to ophthalmologist is strongly recommended.<br>• Initiate treatment with topical corticosteroid eye drops and topical immunosuppressive therapy.<br>• If event resolves to Grade 1 or better, resume atezolizumab. [b]<br>• If event does not resolve to Grade 1 or better while withholding atezolizumab, permanently discontinue atezolizumab and contact the MiST Programme Team.[c]

Ocular event, Grade 3 or 4	• Permanently discontinue atezolizumab and contact The MiST Programme Team.[c]<br>• Refer patient to ophthalmologist.<br>• Initiate treatment with corticosteroids equivalent to 1–2 mg/kg/day oral prednisone.<br>• If event resolves to Grade 1 or better, taper corticosteroids over ≥ 1 month.

[a] Atezolizumab may be withheld for a longer period of time (i.e., > 12 weeks after event onset) to allow for corticosteroids (if initiated) to be reduced to the equivalent of ≤ 10 mg/day oral prednisone. The acceptable length of the extended period of time must be agreed upon by the investigator and the The MiST Programme Team.

[b] If corticosteroids have been initiated, they must be tapered over ≥ 1 month to the equivalent of ≤ 10 mg/day oral prednisone before atezolizumab can be resumed.

[c] Resumption of atezolizumab may be considered in patients who are deriving benefit and have fully recovered from the immune-related event. Patients can be re-challenged with atezolizumab only after approval has been documented by both the MiST Programme Team.

12.1.4 Dermatological Events

Treatment-emergent rash has been associated with atezolizumab. The majority of cases of rash were mild in severity and self-limited, with or without pruritus. A dermatologist should evaluate persistent and/or severe rash or pruritus. A biopsy should be considered unless contraindicated.

Table 2: Management of dermatological events

**Severity**	**Management**
Grade 1	• Continue atezolizumab.<br>• Consider treatment with topical corticosteroids and/or other symptomatic therapy (e.g., antihistamines).

Grade 2	• Continue atezolizumab.<br>• Consider patient referral to dermatologist.<br>• Initiate treatment with topical corticosteroids.<br>• Consider treatment with higher-potency topical corticosteroids if event does not improve.
Grade 3	• Withhold atezolizumab for up to 12 weeks after event onset. [a]<br>• Refer patient to dermatologist.<br>• Initiate treatment with corticosteroids equivalent to 10 mg/day oral prednisone, increasing dose to 1–2 mg/kg/day if event does not improve within 48–72 hours.<br>• If event resolves to Grade 1 or better, resume atezolizumab. [b]<br>• If event does not resolve to Grade 1 or better while withholding atezolizumab, permanently discontinue atezolizumab and contact The MiST Programme Team. [c]
Grade 4	• Permanently discontinue atezolizumab and contact the MiST Programme Team. [c]

[a] Atezolizumab may be withheld for a longer period of time (i.e., > 12 weeks after event onset) to allow for corticosteroids (if initiated) to be reduced to the equivalent of ≤ 10 mg/day oral prednisone. The acceptable length of the extended period of time must be agreed upon by the investigator and the MiST Programme Team.

[b] If corticosteroids have been initiated, they must be tapered over ≥ 1 month to the equivalent of ≤10 mg/day oral prednisone before atezolizumab can be resumed.

[c] Resumption of atezolizumab may be considered in patients who are deriving benefit and have fully recovered from the immune-related event. Patients can be re-challenged with atezolizumab only after approval has been documented by the MiST Programme Team.

12.1.5 Renal Events

Immune-related nephritis has been associated with the administration of atezolizumab. Eligible patients must have adequate renal function, and renal function, including serum creatinine, should be monitored throughout study treatment. Patients with abnormal renal function should be evaluated and treated for other more common etiologies (including prerenal and postrenal causes, and concomitant medications such as non steroidal anti-inflammatory drugs). Refer the patient to a renal specialist if clinically indicated. A renal biopsy may be required to enable a definitive diagnosis and appropriate treatment. Patients with signs and symptoms of nephritis, in the absence of an identified alternate etiology, should be treated according to the guidelines in Table 3.

Table 3 Management Guidelines for Renal Events

Event	Management
Renal event,<br>Grade 1	· Continue atezolizumab.<br>· Monitor kidney function, including creatinine, closely until values resolve to within normal limits or to baseline values.
Renal event,<br>Grade 2	· Withhold atezolizumab for up to 12 weeks after event onset. [a]<br>· Refer patient to renal specialist.<br>· Initiate treatment with corticosteroids equivalent to 1-2 mg/kg/day oral prednisone.<br>· If event resolves to Grade 1 or better, resume atezolizumab. [b]<br>· If event does not resolve to Grade 1 or better while withholding atezolizumab, permanently discontinue atezolizumab and contact MiST Programme Team. [c]
Renal event,<br>Grade 3 or 4	· Permanently discontinue atezolizumab and contact MiST Programme Team.<br>· Refer patient to renal specialist and consider renal biopsy.

	· Initiate treatment with corticosteroids equivalent to 1-2 mg/kg/day oral prednisone.<br>· If event does not improve within 48 hours after initiating corticosteroids, consider adding an immunosuppressive agent.<br>· If event resolves to Grade 1 or better, taper corticosteroids over ≥1 month.

[a] Atezolizumab may be withheld for a longer period of time (i.e., > 12 weeks after event onset) to allow for corticosteroids (if initiated) to be reduced to the equivalent of ≤10 mg/day oral prednisone. The acceptable length of the extended period of time must be agreed upon by the investigator and the Chief Investigator.

[b] If corticosteroids have been initiated, they must be tapered over ≥1 month to the equivalent of ≤10 mg/day oral prednisone before atezolizumab can be resumed.

[c] Resumption of atezolizumab may be considered in patients who are deriving benefit and have fully recovered from the immune-related event. Patients can be re-challenged with atezolizumab only after approval has been documented the investigator (or an appropriate delegate) and the Chief Investigator.

12.1.6 **Systemic immune activation**

Systemic immune activation is a rare condition characterized by an excessive immune response. Given the mechanism of action of atezolizumab, systemic immune activation is considered a potential risk when atezolizumab is given in combination with other immunomodulating agents.

Recommendations regarding early identification and management of systemic immune activation are provided below. In the event of suspected systemic immune activation, atezolizumab should be withheld and clinical specialists (e.g., rheumatology, clinical immunology, or solid organ or hematopoietic stem cell transplant specialists) and the MiST Programme Team should be consulted for additional guidance.

Early disease recognition is critical, and systemic immune activation should be suspected if, in the absence of an alternative etiology, the patient meets two or more of the following criteria:

- Hypotension that is refractory to aggressive IV fluid challenge

    Vasopressor support may be required.

- Respiratory distress that requires aggressive supportive care

    Supplemental oxygen and intubation may be required.

- Fever > 38.5°C
- Acute renal or hepatic failure
- Bleeding from coagulopathy
- Any of the following unexplained laboratory abnormalities (change from baseline): cytopenias (in two or more lineages), significant transaminitis, or coagulopathy
- For patients with suspected systemic immune activation, an initial evaluation should include the following:
- FBC with peripheral smear
- PT, PTT, fibrinogen, and D-dimer
- Ferritin
- Soluble interleukin 2 (IL-2) receptor (soluble CD25)
- Triglycerides
- AST, ALT, and total bilirubin
- LDH
- Complete neurologic and abdominal examination (assess for hepatosplenomegaly)

Laboratory tests with normal results should be repeated frequently in patients for whom a high clinical suspicion of systemic immune activation exists.

If neurologic abnormalities are present, consider cerebrospinal fluid analysis and/or an MRI of the brain.

If cytopenias are present (Grade ≥ 2 in two or more lineages) or ferritin is ≥ 3000 ng/mL, the following evaluations should also be performed:

- Bone marrow biopsy and aspirate (assess for evidence of hemophagocytosis)
- Soluble interleukin 2 (IL-2) receptor (sCD25)
- Natural killer cell activity
- Adenovirus, cytomegalovirus, Epstein-Barr virus, herpes-simplex virus, and human herpesvirus 6, 7, and 8 evaluation (for reactivated or active disease)

Diagnostic criteria and recommended management for systemic immune activation are provided in Table 4. The diagnostic criteria apply only when alternative etiologies have been excluded.

An adverse event of systemic immune activation should be reported on the Adverse Event eCRF if it meets the criteria for "consistent with systemic immune activation" or "probable systemic immune activation" as outlined in Table 4.

Table 4 Diagnostic Criteria and Recommended Management for Systemic Immune Activation

Systemic Immune Activation Diagnostic Criteria (applicable only when alternative etiologies have been excluded)	
Major Criteria	Minor Criteria
• Fever ≥ 38.5°C on more than one occasion<br>• Ferritin ≥ 3000 ng/mL<br>• Cytopenias (Grade ≥ 2 in two or more lineages)<br>• Age-adjusted soluble interleukin-2 receptor elevated by ≥ 2 standard deviations<br>• Severe (Grade ≥ 3) or progressive dysfunction in two or more organs<br>• Decreased fibrinogen	• Splenomegaly<br>• Hemophagocytosis in bone marrow, spleen, or lymph nodes<br>• Elevated γ-glutamyl transpeptidase (GGT) or liver function tests (AST, ALT, or direct bilirubin)<br>• Elevated triglycerides<br>• Elevated LDH<br>• Decreased natural killer cell activity

Diagnosis and Management of Systemic Immune Activation		
Number of Criteria	Diagnosis	Action to Be Taken
≥ 4 major criteria	Consistent with systemic immune activation	• Permanently discontinue atezolizumab.<br>• Consider treatment with an immunosuppressive agent (i.e., cytokine inhibitors) and IV corticosteroids (i.e., methylprednisolone 1 g once daily or equivalent, or dexamethasone ≥ 10 mg/m^2 once daily if neurologic abnormalities are present).

		• Contact the MiST Programme Team for additional recommendations.<br>• Consider HLH-2004 protocol if there is no clinical improvement.
3 major criteria<br><u>OR</u><br>2 major plus<br>≥ 3 minor criteria	Probable systemic immune activation	• Depending on clinical severity, follow guidelines for "Consistent with systemic immune activation" or "Possible systemic immune activation" diagnosis.<br>• Clinical specialists and the MiST Programme Team may be contacted for recommendations.
2 major plus<br>≤ 2 minor criteria<br><u>OR</u><br>1 major plus<br>≥ 4 minor criteria	Possible systemic immune activation	• Withhold atezolizumab.<br>• Consider treatment with IV corticosteroids.<br>• Clinical specialists and the MiST Programme Team may be contacted for additional recommendations.<br>• Follow guidelines for "Consistent with systemic immune activation" diagnosis if there is no clinical improvement or if clinical worsening occurs.<br>• If clinical improvement occurs, atezolizumab may be resumed following a benefit-risk assessment by the MiST Programme Team.

Notes: Criteria are adapted from a Delphi Survey of 26 experts who provided helpful criteria in the positive diagnosis of hemophagocytic syndrome in adult patients (Hejblum et al. 2014).

Grades are based on National Cancer Institute Common Terminology Criteria for Adverse Events.

These recommendations do not replace clinical judgment and are intended as suggested guidance.

12.2 Management of overdoses

No information on overdose has been established at this time. In the clinical development for atezolizumab, the highest dose that has been exposed to patients is 1200mg every 3 weeks.

12.3 Bevacizumab

12.3.1 Bevacizumab dose and dose interval modification

The bevacizumab dose will be based on the patient's weight at baseline (Cycle 1, Day 1) and will remain the same throughout the study, unless there is a weight change of >10% from baseline. It is not necessary to correct on the basis of ideal weight. Management of bevacizumab toxicity should be managed as per Manufacturer guidelines (Please refer to SPC). Suggested guidelines for management are summarised in table 5.

Patients who discontinue bevacizumab transiently or permanently may continue on single agent atezolizumab until disease progression or if there is felt to be clinical benefit. Patients with Grade ≥3 toxicities attributable to bevacizumab should hold atezolizumab until those toxicities have improved to Grade ≤2 (exception for grade 3 hypertension). If bevacizumab is permanently discontinued but there is felt to be clinical benefit from atezolizumab, this may be continued.

Temporary suspension of bevacizumab must occur if a patient experiences a serious adverse event or a Grade 3 or 4 non serious adverse event assessed by the investigator as related to bevacizumab. If the event resolves to ≤1, bevacizumab may be restarted at the same dose level. Patients who develop grade 4 toxicities related to bevacizumab for > 21 days should permanently discontinue bevacizumab.

The appropriate interval between the last dose of bevacizumab and major surgery is unknown. For elective surgery, bevacizumab should be withheld for a 28 day peri-operative period. Re-initiation should not occur until wounds have fully healed. Re-initiation after surgery requires documented approval from the MiST Programme Team.

Infusion of bevacizumab should be interrupted in patients who develop dyspnoea or clinically significant hypotension. Patients who experience an NCI CTCAE grade 3 or 4 allergic reaction/hypersensitivity, adult respiratory distress syndrome, or bronchospasm (regardless of grade) will be discontinued from bevacizumab treatment.

Bevacizumab infusion should be slowed to $\leq$ 50% or interrupted for patients who experience any infusion-associated symptoms not specified above. If the infusion is interrupted, it may be resumed at $\leq$ 50% of the rate prior to reaction after the patient's symptoms have adequately resolved and increased in 50% increments up to the full rate if tolerated. Infusions may with subsequent cycles be restarted at the full rate if the full rate is tolerated again previously.

Table 5: Recommended guidelines for management of suspected bevacizumab toxicities

Event	Action to be taken
**Hypertension**	
Grade 1 (asymptomatic, transient [< 24 hr] blood pressure increase by > 20 mmHg (diastolic) or to > 150/100 mmHg if previously within normal limits)	No modifications
Grade 2 (recurrent or persistent [> 24 hr] or symptomatic increase by > 20 mmHg (diastolic) or to > 150/100 mmHg if previously within normal limits)	Hold bevacizumab. Start anti-hypertensive therapy per institutional guidelines. After blood pressure is <150/100 mmHg, patient may continue bevacizumab.
Grade 3	Requires more than one antihypertensive drug or more intensive therapy than previously: If not controlled to 150/100 mmHg with medication, discontinue bevacizumab.
Grade 4 (including hypertensive encephalopathy)	Discontinue bevacizumab.

Haemorrhage	
Grade 1 or 2 non-pulmonary or non-CNS events	No bevacizumab modifications
Grade 3 non-pulmonary or non-brain or non-spinal cord haemorrhage	Hold bevacizumab until all of the following criteria are met:<br>• The bleeding has resolved and haemoglobin is stable.<br>• There is no bleeding diathesis that would increase the risk of therapy.<br>• There is no anatomic or pathologic condition that significantly increases the risk of haemorrhage recurrence.<br>Patients who experience a repeat Grade 3 haemorrhagic event will be discontinued from bevacizumab.
Grade 4 non-pulmonary or non-brain or non-spinal cord haemorrhage.	Discontinue bevacizumab
Grade 1 pulmonary or brain or spinal cord haemorrhage	Hold bevacizumab until all of the following criteria are met:<br>• The bleeding has resolved and haemoglobin is stable.<br>• There is no bleeding diathesis that would increase the risk of therapy.<br>• There is no anatomic or pathologic condition that significantly increases the risk of hemorrhage recurrence.
Grade 2,3, or 4 pulmonary or brain or spinal cord haemorrhage	Discontinue bevacizumab
**Venous thrombo-embolism event**	
Grade 1 or 2	No bevacizumab modification

Grade 3 or asymptomatic Grade 4	If the planned duration of full-dose anticoagulation is < 2 weeks, bevacizumab should be held until the full-dose anticoagulation period is over. If the planned duration of full-dose Anticoagulation is > 2 weeks, bevacizumab may be resumed after 2 weeks of full-dose anticoagulation if all of the following criteria are met:<br>• The patient must have an in-range INR (usually between 2 and 3) if on warfarin; LMWH, warfarin, or other anticoagulant dosing must be stable prior to restarting study treatment.<br>• The patient must not have had a Grade 3 or 4 haemorrhagic event while on anticoagulation.
Symptomatic Grade 4	Discontinue bevacizumab
**Arterial thromboembolic event**<br>(new onset, worsening, or unstable angina, myocardial infarction, transient ischemic attack, cerebrovascular accident, and any other arterial thromboembolic event)	
Any grade	Discontinue bevacizumab
**Congestive heart failure** (left ventricular systolic dysfunction)	
Grade 1 or 2	No bevacizumab modifications.
Grade 3	Hold bevacizumab until resolution to Grade ≤ 1.
Grade 4	Discontinue bevacizumab.
**Proteinuria**	

Grade 1 (urine dipstick 1 + or urine collection 0.15 to 1.0 g/24 hr)	No bevacizumab modifications.
Grade 2 (urine dipstick 2 + to 3 + or urine collection > 1.0 to 3.5 g/24 hr)	For 2 + dipstick, may administer bevacizumab and obtain 24-hour urine prior to next dose.<br>For 3 + dipstick, obtain 24-hour urine prior to administration of bevacizumab.<br>Hold bevacizumab for proteinuria >2 g/24hr and resume when proteinuria is ≤ 2 g/24 hr.
Grade 3 (urine dipstick 4 + or urine collection >3.5 g/24 hr)	Hold bevacizumab. Resume when proteinuria is ≤ 2 g/24 hr.
Grade 4 (nephrotic syndrome)	Discontinue bevacizumab
**GI perforation**	
Any grade	Discontinue bevacizumab.
**Fistula**	
Any grade tracheoesophageal fistula	Discontinue bevacizumab.
Grade 4 fistula (other than tracheoesophageal)	Discontinue bevacizumab.
**Bowel obstruction**	
Grade 1	Continue patient on study for partial obstruction <u>not</u> requiring medical intervention.
Grade ≥2	Discontinue bevacizumab.
**Wound dehiscence**	
Any grade (requiring medical or surgical therapy)	Discontinue bevacizumab
**Reversible posterior leukoencephalopathy**	
Any grade	Discontinue bevacizumab
**Fatigue/asthenia**	

Grade 1 or 2	No bevacizumab modification
Grade 3	No bevacizumab modification
Grade 4	No bevacizumab modification
**Hand-foot syndrome**	
Grade 1 or 2	No bevacizumab modification
Grade 3	No bevacizumab modification
**Stomatitis**	
Grade 1 or 2	No bevacizumab modification
Grade 3	No bevacizumab modification
Grade 4	No bevacizumab modification
**Hematologic toxicities (excluding anemia)**	
Grade 1 or 2	No bevacizumab modification
Grade 3 or 4	No bevacizumab modification

12.4 Pregnancy or Drug Exposure during Pregnancy

The Investigator must collect pregnancy information for female trial subjects or female partners of male trial subjects. This includes subjects who become pregnant while they (or their male partner) are participating in a clinical trial of an investigational medicinal product (IMP) or during a stage where the foetus could have been exposed to the IMP.

Fertile male participant must agree to use condoms as a method of contraception, refrain from fathering a child and/or donating sperm during the trial and for 6 months following the last dose of IMP trial treatment.

The reproductive and teratogenicity effects of atezolizumab and bevacizumab are not known therefore patients of childbearing potential (as defined in section 4.2) and their partners, who are sexually active, must agree to the use of 2 highly effective forms of

contraception throughout their participation in the trial and for 6 months after last dose of trial drug. The necessity of adherence to the contraception requirements should be part of the consent process by explaining clearly to the patient the potential dangers of becoming pregnant and also providing each patient with information about appropriate medically approved contraception. Birth control methods which are considered as highly effective include:

- Combined (estrogen and progestogen containing)[1]:
    - o oral
    - o intravaginal
    - o transdermal
    - o Injectable

***Please Note:** hormonal methods are acceptable in WOCBP participants, and female partners of male participant, **only** if associated with the inhibition of ovulation.*

- progestogen-only hormonal contraception [1]:
    - o oral
    - o injectable
    - o implantable [2]

***Please Note:** hormonal methods are acceptable in WOCBP participants, and female partners of male participant, **only** if associated with the inhibition of ovulation.*

- intrauterine device (IUD) [2]
- intrauterine hormone-releasing system (IUS) [2]
- bilateral tubal occlusion [2]
- vasectomised partner [2, 3]
- sexual abstinence [4]

1 Hormonal contraception may be susceptible to interaction with the IMP, which may reduce the efficacy of the contraception method

2 Contraception methods that in the context of this guidance are considered to have low user dependency.

3 Vasectomised partner is a highly effective birth control method provided that partner is the sole sexual partner of the WOCBP trial participant and that the vasectomised partner has received medical assessment of the surgical success.

4 In the context of this guidance sexual abstinence is considered a highly effective method only if defined as refraining from heterosexual intercourse during the entire period of risk associated with the study treatments. The reliability of sexual abstinence needs to be evaluated in relation to the duration of the clinical trial and the preferred and usual lifestyle of the subject

It is currently unknown whether atezolizumab and bevacizumab may reduce the effectiveness of systemically acting hormonal contraceptives, and therefore women using systemically acting hormonal contraceptives will be advised to add a barrier method.

Contraceptives should be effective before Day 1 of drug administration, throughout the trial and for 6 months after the last IMP dose.

Female partners of male participants must follow **highly effective** methods of contraception as detailed above. It should be explained to male patients that if his partner is pregnant or breast-feeding when he enters the trial, the patient should use barrier method contraception to prevent the unborn baby or the baby being exposed to the trial drug.

The Investigator must ensure that all patients are aware at the start of a clinical trial of the importance of reporting all pregnancies (in themselves and their partners) that occur whilst being treated with IMP and occurring up to the time specified in the drug-specific appendix after the last IMP administration. The Investigator should offer counselling to the patient and/or the partner, and discuss the risks of continuing with the pregnancy and the possible effects on the foetus. Monitoring of the patient and the baby should continue until the conclusion of the pregnancy, if the patient or patient’s partner has consented to this.

If a patient becomes pregnant during the study the investigator is to stop dosing with atezolizumab and/or bevacizumab immediately.

Lactation

It is unknown whether atezolizumab or bevacizumab excreted in human breast milk. Because of the potential for serious adverse reactions in breastfed infants, lactating women should be advised not to breastfeed while taking atezolizumab and/or bevacizumab and for at least 6 months after the last dose.

13. PRIOR AND CONCOMITANT THERAPIES

Concomitant therapy includes any medication (e.g., prescription drugs, over-the-counter drugs, herbal or homeopathic remedies, nutritional supplements) used by a patient from 7 days prior to screening until the treatment discontinuation visit. All such medications should be reported to the investigator.

Premedication with antihistamines may be administered for any atezolizumab infusions after Cycle 1.

The following therapies should continue while patients are in the study:

- Oral contraceptives
- Hormone-replacement therapy
- Prophylactic or therapeutic anticoagulation therapy (such as low molecular−weight heparin, direct thrombin inhibitors, or warfarin at a stable dose level)
- Megestrol administered as an appetite stimulant
- Inhaled corticosteroids for chronic obstructive pulmonary disease
- Mineralocorticoids (e.g., fludrocortisone)
- Low-dose corticosteroids (≤10 mg prednisone equivalent) for patients with orthostatic hypotension or adrenocortical insufficiency

In general, investigators should manage a patient's care with supportive therapies as clinically indicated, as per local standards. Patients who experience infusion-associated symptoms may be treated symptomatically with acetaminophen, ibuprofen, diphenhydramine, and/or ranitidine or another H2 receptor antagonist as per standard practice.

Serious infusion-associated events manifested by dyspnea, hypotension, wheezing, bronchospasm, tachycardia, reduced oxygen saturation, or respiratory distress should be managed with supportive therapies as clinically indicated (e.g., supplemental oxygen and β2-adrenergic agonists).

The use of systemic corticosteroids is discouraged. A limited course of systemic steroids is permitted to treat immune-mediated adverse events when associated with Atezolizumab therapy, at the discretion of the treating physician.

13.1 Atezolizumab

Patients, who have received prior treatment with a PD-LI/PD-1 inhibitor including atezolizumab, are not eligible to participate in this study.

Atezolizumab is contraindicated for patients with a history of severe allergic anaphylactic reactions to chimeric, human or humanized antibodies, or fusion proteins because of the potential for severe reactions.

In addition atezolizumab is contraindicated in patients with a known hypersensitivity to CHO cell products or any component of the atezolizumab formulation.

During the study, supportive care (e.g., antiemetics; analgesics for pain control) may be used at the investigator’s discretion and in accordance with institutional procedures. All procedures performed (e.g., thoracentesis, etc.) and medications used during the study must be documented on the eCRF.

13.1.1 Concomitant, Anticancer or Experimental Therapy

No other anticancer therapies (including chemotherapy, radiation, hormonal treatment, antibody or other immunotherapy, gene therapy, vaccine therapy, angiogenesis inhibitors, or other experimental drugs) of any kind will be permitted while the patient is participating in the study, with the exception of hormonal treatment.

13.1.2 Excluded and cautionary therapy for atezolizumab treated patients

The following medications are excluded while the patient is receiving study treatment:

- Herbal medicines; these therapies are not fully studied and their use may result in unanticipated drug-drug interactions that may cause or confound the assessment of toxicity.
- RANKL inhibitor (denosumab)Immunomodulatory agents, including but not limited to interferons orinterleukin-2 during the entire study; these agents could potentially increase the risk of autoimmune conditions when received in combination with atezolizumab

- Immunosuppressive medications, including but not limited to cyclophosphamide, azathioprine, methotrexate, and thalidomide; these agents could potentially alter the activity and the safety of atezolizumab.
- Influenza vaccinations (inactivated forms only) should be given only during influenza season. Patients must agree not to receive live, attenuated influenza vaccines (such as FluMist®) 28 days prior to randomization, at any time during the study, or within 90 days following the last dose of atezolizumab but may receive inactivated influenza vaccines.
- In addition, patients treated with atezolizumab should not receive other immunomodulatory agents for 10 weeks after study treatment discontinuation.

13.1.3 Hematopoietic Growth Factors and Blood Products

Erythropoietin, darbepoetin alfa, and/or hematopoietic colony-stimulating factors for treatment of cytopenias should be administered according to institutional guidelines. Transfusion thresholds for blood product support will be in accordance with institutional guidelines.

13.1.4 Drug interactions

Cytochrome P450 enzymes as well as conjugation/glucuronidation reactions are not involved in the metabolism of atezolizumab. No drug interaction studies for atezolizumab have been conducted or are planned. There are no known interactions with other medicinal products or other form of interactions.

13.1.5 Bisphosphonates

Bisphosphonates are allowed for prevention of skeletal events, but not for treatment of symptomatic hypercalcemia.

13.1.6 Other Concomitant Medications

Therapies considered necessary for the patient's well-being may be given at the discretion of the investigator and should be documented on the eCRF. Other concomitant medications, except for analgesics, chronic treatments for concomitant medical conditions, or agents required for life threatening medical problems, should be avoided. Herbal and complementary therapies should not be encouraged because of unknown side effects and

potential drug interactions, but any taken by the patient should be documented appropriately on the eCRF.

13.2 Bevacizumab

Patients, who have received prior treatment with VEGF therapy including bevacizumab, are not eligible to participate in this study.

During the study, supportive care (e.g., antiemetics; analgesics for pain control) may be used at the investigator's discretion and in accordance with institutional procedures. All procedures performed (e.g., thoracentesis, etc.) and medications used during the study must be documented on the eCRF.

13.2.1 Concomitant, Anticancer or Experimental Therapy

No other anticancer therapies (including chemotherapy, radiation, hormonal treatment, antibody or other immunotherapy, gene therapy, vaccine therapy, angiogenesis inhibitors, or other experimental drugs) of any kind will be permitted while the patient is participating in the study, with the exception of hormonal treatment.

13.2.2 Other Concomitant Medications

Therapies considered necessary for the patient's well-being may be given at the discretion of the investigator and should be documented on the eCRF. Other concomitant medications, except for analgesics, chronic treatments for concomitant medical conditions, or agents required for life threatening medical problems, should be avoided. Herbal and complementary therapies should not be encouraged because of unknown side effects and potential drug interactions, but any taken by the patient should be documented appropriately on the eCRF.

14. DOSAGE, FORM, PACKAGING, AND LABELLING OF IMP.

Patients will be dispensed drug as prescribed. No dose modifications are permitted.

Atezolizumab drug product is provided in a single-use, 20-mL USP/Ph. Eur. Type 1 glass vial as a colourless-to-slightly-yellow, sterile, preservative-free clear liquid solution intended for IV administration. The vial is designed to deliver 20 mL (1200 mg) of atezolizumab solution, but may contain more than the stated volume to enable delivery of the entire 20 mL volume.

Bevacizumab is supplied in 400-mg (25-mg/mL) glass vial contains 16 mL of bevacizumab (25 mg/mL) and 100mg (25-mg/mL) glass vial containing 4 mL of bevacizumab (25 mg/mL) with a vehicle consisting of sodium phosphate, trehalose, polysorbate 20, and Sterile Water for Injection, USP.

14.1 Storage of study treatment

Atezolizumab must be refrigerated at 2°C–8°C (36°F–46°F) upon receipt until use.

Atezolizumab vials should not be used beyond the expiration date provided by the manufacturer. No preservative is used in the atezolizumab drug product; therefore, each vial is intended for single use only. Vial contents should not be frozen or shaken and should be protected from direct sunlight.

Bevacizumab vials contain no preservative and are for single use only. It should be stored in the vials, in a refrigerator (2°C-8°C). Do not freeze. Keep the vial in the outer carton in order to protect from light. Bevacizumab will be labelled according to local regulatory requirements.

14.2 Accountability of the Study Treatment

The study medication will be supplied by Roche/Genentech, Inc. to the participating sites pharmacy departments. Any unused medication and/or used vials/packaging will be returned to Roche/Genentech, Inc. at the end of the study. All movements of study medication between Roche/Genentech, Inc. and pharmacy will be documented.

The Investigator will use a standard prescription form and a member of the Investigator team will collect the medication at each clinic visit prior to each cycle of treatment.

15. SAFETY REPORTING

15.1 Serious Adverse Event

15.1.1 Definitions

An **Adverse Event (AE)** is defined as “any untoward medical occurrence in a participant associated with the use of an investigational medicinal product, whether or not considered related to this treatment.”

An **Adverse reaction (AR)** is defined as “any untoward and unintended occurrence response in a participant to an investigational medicinal product, related to any dose administered.”

A **Serious Adverse Event (SAE)** is defined as “any adverse event or adverse reaction in a participant that:

- Results in death
- Is life threatening (the subject was at risk of death at the time of event)
- Requires hospitalisation or prolongation of an existing hospitalisation
- Results in persistent or significant disability or incapacity
- Consists of a congenital anomaly or birth defect
- Other serious Important Medical Event - an event that may not be immediately life threatening or result in death or hospitalisation but may jeopardise the subject or may require intervention to prevent one of the outcomes listed above should be considered.

Note: In addition to the above criteria, the following adverse events are reportable to the drug providers in the same timeframe as SAEs:

- Is a new cancer (that is not a condition of the study);
- Is associated with an overdose.

Events or Outcomes Not Qualifying as Serious Adverse Events:

The following are not considered SAEs and therefore do not need to be reported as such:

- Pre-planned or elective hospitalisation including social and/ or convenience situations (e.g., respite care)

- Hospital visits of less than 24 hours duration (e.g., patient presents to the emergency room, but is not admitted to a ward)
- Overdose of atezolizumab and/or bevacizumab or concomitant medication unless the event meets SAE criteria (e.g. hospitalisation). However, the event should still be captured as a non-serious AE on the appropriate CRF page.
- Events of progression of the patient's underlying cancer as well as events clearly related to progression of the patient's cancer (signs and symptoms of progression) should not be reported as a serious adverse event unless the outcome is fatal during the study or within the safety reporting period. If the event has a fatal outcome during the study or within the safety reporting period, then the event of Progression of Disease must be recorded as an SAE with CTC Grade 5 (fatal outcome) indicated. For events of progression that do not result in death, they should be captured on the AE CRF as a NONSERIOUS event only.

A **Serious Adverse Reaction (SAR)** is defined as "an adverse reaction that in its nature is serious and which is consistent with the information about the medicinal product listed in the relevant reference documentation – Investigator Brochure (IB) or Summary of Product Characteristics (SmPC)".

A **Suspected Unexpected Serious Adverse Reaction (SUSAR)** is defined as "a serious adverse reaction, the nature and severity of which is not consistent with the applicable product information in the Investigator Brochure (IB) or Summary of Product Characteristics (SmPC)".

Adverse Event of Special Interest (AESIs)

An adverse event of special interest (AESI; serious or non-serious) is one of scientific and medical concern specific to the investigational medicinal product, for which ongoing monitoring and rapid communication by the investigator to the drug provider can be appropriate. Such an event might warrant further investigation in order to characterise and understand it. Depending on the nature of the event, rapid communication by the investigator or delegate to other parties (e.g., regulators) might also be warranted.

Adverse events of special interest for this study include the following:

Bevacizumab (Avastin®) List of AESI's:

- Bleeding/ Haemorrhage
- Congestive Heart Failure (CHF)
- Fistula/ Abscess (Non GI)
- Gastrointestinal perforation
- Hypertension
- Posterior Reversible Encephalopathy Syndrome (PRES)
- Proteinuria
- Thromboembolic Event - Arterial (ATE)
- Thromboembolic Event - Venous (VTE)
- Wound Healing Complications

Atezolizumab (Tecentriq®) List of AESI's:

- Cases of potential drug-induced liver injury that include an elevated ALT or AST in combination with either an elevated bilirubin or clinical jaundice, as defined by Hy's Law (https://www.fda.gov/downloads/guidances/UCM174090.pdf).
- Suspected transmission of an infectious agent by the study treatment, as defined below.
- Any organism, virus, or infectious particle (e.g., prion protein transmitting transmissible spongiform encephalopathy), pathogenic or non-pathogenic, is considered an infectious agent. A transmission of an infectious agent may be suspected from clinical symptoms or laboratory findings that indicate an infection in a patient exposed to a medicinal product. This term applies only when a contamination of study treatment is suspected.

- Pneumonitis
- Colitis
- Endocrinopathies: diabetes mellitus, pancreatitis, adrenal insufficiency, hyperthyroidism, and hypophysitis
- Hepatitis, including AST or ALT>10 >ULN
- Systemic lupus erythematosus
- Neurological disorders: Guillain-Barré syndrome, myasthenic syndrome or myasthenia gravis, and meningoencephalitis
- Events suggestive of hypersensitivity, infusion-related reactions, cytokine-release syndrome, influenza like illness, systemic inflammatory response syndrome, and systemic immune activation
- Nephritis
- Ocular toxicities (e.g., uveitis, retinitis, optic neuritis)
- Myositis
- Myopathies, including rhabdomyolysis
- Grade > 2 cardiac disorders (e.g., atrial fibrillation, myocarditis, pericarditis)
- Vasculitis
- Autoimmune haemolytic anaemia
- Severe cutaneous reactions (e.g., Stevens-Johnson syndrome, dermatitis bullous, toxic epidermal necrolysis)

Such events must be reported immediately and within 24 hours to Sponsor and 48 hours to Roche following first awareness as SAEs “Considered medically significant by the Investigator”. The requirement to report the specified AESI’s as SAEs continues with no time limit post administration of atezolizumab and/or bevacizumab or the end of the trial.

Special Situations:

Special situations are defined as:

- Accidental overdose: accidental administration of a drug in a quantity that is higher than the assigned dose.

- Medication error: accidental deviation in the administration of a drug
  In some cases, a medication error may be intercepted prior to administration of the drug.

Special situations are not in themselves adverse events, but may result in adverse events which must be recorded. Should Special Situations result in an AE's (for example; a headache), two adverse events should be listed (one for the overdose/medical error and one for the headache). If the associated adverse event fulfils seriousness criteria, the event should be reported to the Sponsor and Roche immediately (i.e., no more than 24 hours after learning of the event).

15.2 Safety Assessments

All Adverse events (AEs) will be recorded throughout the study whether serious or not. Members of the research team will ask the participants about AEs at each study time point and will complete the participant AE form, which will be a continuous log so as to capture end dates, and the study serious adverse event (SAE) log. This will be evaluated and classed according to the definitions in sections 15.1.

Severity of Adverse Event

The severity of each AE will be graded using the NCI CTCAE, Version 4.03 grading scale http://www.eortc.be/services/doc/ctc/ctcae_4.03_2010-06-14_quickreference_5x7.pdf
For AEs not covered by NCI CTCAE, the severity will be characterised as mild, moderate, severe, life-threatening or fatal according to the following definitions:

- Mild events are usually transient and do not interfere with the patient's daily activities.

- Moderate events introduce a low level of inconvenience or concern to the patient and may interfere with daily activities.

- Severe events interrupt the patient's usual daily activities and hospitalisation (or prolongation of hospitalisation) may be required.
- Life-threatening events require urgent intervention to prevent death.

15.3 Clinical Laboratory Assessments as Adverse Events

It is the responsibility of the investigator to assess the clinical significance of all abnormal values as defined by the list of reference ranges from the local laboratory. In some cases, significant changes in lab values within the normal range will require similar judgment.

An abnormal laboratory value that is not already associated with an AE is to be recorded as an AE only if any one of the following criteria is met:

- An action on one or both of the study drugs is made as a result of the abnormality
- Intervention for management of the abnormality is required.
- At the discretion of the investigator should the abnormality be deemed clinically significant.

15.4 Causality and Expectedness Assessments

15.4.1 Reference Safety information

Please refer to the relevant table of the current Investigator Brochure for atezolizumab and bevacizumab. These tables will be required to assess expectedness of an event and thus the SAE or SUSAR reporting requirements. Roche will be responsible for maintaining the Investigator's Brochures for atezolizumab and bevacizumab. Roche will be responsible for informing the Sponsor and/or site of any updates to the Investigator's Brochure during the trial.

15.4.2 Causality

For causal relationship of adverse events to atezolizumab and bevacizumab, medical judgement should be used to determine:

- The cause of the AE considering all relevant factors such as, but not limited to, the underlying study
- Indication, coexisting disease, concomitant medication, relevant history, pattern of the AE, temporal relationship to atezolizumab and bevacizumab, de-challenge or re-challenge.

Table 6: Causal Attribution Guidance for atezolizumab and bevacizumab

Not related to atezolizumab and bevacizumab	• An AE that is clearly due to extraneous causes (eg, concurrent disease, concomitant medications, disease under study, etc.)<br>• It does not follow a reasonable temporal sequence from administration of atezolizumab and bevacizumab.<br>• It does not follow a known pattern of response to atezolizumab and bevacizumab<br>• It does not reappear or worsen when atezolizumab and bevacizumab is restarted.<br>• An alternative explanation is likely, but not clearly identifiable.
Related to<br>atezolizumab and bevacizumab	• An AE that is difficult to assign to alternative causes.<br>• It follows a strong or reasonable temporal sequence from administration of atezolizumab and bevacizumab.<br>• It could not be reasonably explained by the patient's clinical state,<br>• concurrent disease, or other concomitant therapy administered to the patient.<br>• It follows a known response pattern to atezolizumab and bevacizumab.<br>• It is confirmed with a positive re-challenge or supporting laboratory data.

Outcome and Action Taken for an Adverse Event

The investigator will record the action taken and outcome for each AE according to the following criteria:

Action Taken with atezolizumab and bevacizumab:

- None
- atezolizumab and/or bevacizumab temporarily interrupted
- atezolizumab and/or bevacizumab permanently discontinued
- Other (specify)

Outcome

- Recovered
- Recovered with sequelae
- Recovering/ Resolving
- Ongoing
- Death
- Lost to follow-up

15.5 Recording and Reporting of AEs, ARs, SAEs, SARs, SUSARs, AESIs and Special Situations

15.5.1 AEs and ARs

AEs and ARs will be recorded from the time of consent until the participant has completed the study and they will be followed-up until they have resolved. Whenever possible, medical terminology should be used and the investigator should combine signs and symptoms that constitute a single disease entity or syndrome into a final diagnosis, if appropriate. For example, fever, cough, and shortness of breath may be reported as pneumonia, if that is a reasonable diagnosis. AE and AR assessments should be made by the PI. Each AE/AR is to be

evaluated for duration, severity, seriousness, and causal relationship to the study drugs. The action taken and the outcome must also be recorded.

15.5.2 SAEs, SARs, SUSARs, AESIs and Special Situations

For the time period beginning when the consent form is signed until treatment allocation, any serious adverse event (including AESI's and Special Situations), or follow up to a serious adverse event, including death due to any cause that occurs to any participant should be recorded on the Medical History CRF and must be reported immediately and within 24 to the MiSTsafety@le.ac.uk mailbox copying in uolsponsor@le.ac.uk if it causes the participant to be excluded from the study, or is the result of a protocol-specified intervention, including but not limited to washout or discontinuation of usual therapy or a procedure.

For the time period beginning at treatment allocation, 90 days following cessation of treatment OR 30 days following cessation of treatment if the participant initiates new anticancer therapy (whichever is earlier), any serious adverse event (including AESI's and Special Situations), or follow up to a serious adverse event, including death due to any cause whether or not related to the Roche product, must be reported immediately and within 24 hours to the MiSTsafety@le.ac.uk mailbox copying in uolsponsor@le.ac.uk, all events should be recorded in the AE CRF.

The MiST Programme Team will forward any reported serious adverse events onto Roche for review within 48 hours of knowing of the event. All events should be recorded in the AE CRF. SAEs brought to the attention of an investigator at any time outside of the time period specified above must be reported immediately to the MiST Programme Team (as delegates of the Sponsor) via the MiSTsafety@le.ac.uk mailbox if the event is considered to be drug-related. It is important that the PI or medically qualified delegate provide an assessment of the expectedness and causality of the SAE or SAR to atezolizumab and bevacizumab at the time of the initial report by consulting the approved Reference Safety Information.

The contact information for the reporting of SAEs can be found on the SAE Reporting Form. AEs/SAEs assessments should be made by the PI. If Roche decides to reclassify an SAE/SAR after it is sent from the MiST Programme Team, Roche will notify the Sponsor within 7 calendar days from the date of decision.

All SAEs or SARs assigned by the PI or delegate (or following central review) as both suspected to be related to IMP-treatment and unexpected will be classified as SUSARs and will be subject to expedited reporting to the Medicines and Healthcare Products Regulatory Agency (MHRA). The MiST Programme Team (as delegates of the Sponsor) will inform the MHRA and the REC of SUSARs within the required expedited reporting timescales. Fatal or life-threatening SUSARs must be reported within 7 days and all other SUSARs within 15 days.

In addition to the expedited reporting above, the Chief Investigator will submit once a year throughout the clinical study or on request a Developmental Safety Update Report (DSUR) to the MHRA and REC and includes SAE data. A copy of the Executive Summary will be provided to Roche within 5 working days of finalisation.

The MiST Programme Team (as Sponsor delegates) is required to provide Roche with a trial progress report each quarter. Roche will send the Sponsor/MiST Programme Team a monthly listing of any SAEs received from Sponsor. MiST Programme Team/Sponsor will review the listing and notify Roche of any omissions.

15.6 Follow-Up of Adverse Events and Serious Adverse Events

All AEs occurring during the study are to be followed up in accordance with good medical practice until Grade 1 or less; judged no longer clinically significant; or, if a chronic condition, until fully characterised through 30 days after the last dose of treatment. Any SAEs must be followed until resolution or stabilization, or until lost to follow-up. After the 30 day window, only treatment related SAE's need to be reported.

15.7 Pregnancy or Drug Exposure During Pregnancy

If a female participant (or female partner of a male participant) becomes pregnant during the study, the investigator is to stop dosing with atezolizumab and/or bevacizumab immediately. Although pregnancy and infant exposure during breast feeding are not considered adverse events, it is the responsibility of investigators or their designees to report any pregnancy or lactation in a participant (spontaneously reported to them) that occurs during the study. Such events must also be reported to the MiST Programme Team, Sponsor and Roche immediately and within 48 hours of knowing about the event.

Pregnancies and infant exposures during breastfeeding that occur after the consent form is signed but before treatment allocation must be reported by the investigator if they cause the participant to be excluded from the study, or are the result of a protocol-specified intervention, including but not limited to washout or discontinuation of usual therapy, diet or a procedure. Pregnancies and infant exposures during breastfeeding that occur from the time of treatment allocation through to 6 months following cessation of the treatment, or 30 days following cessation of treatment if the participant initiates new anticancer therapy, whichever is earlier, must be reported by the investigator. All reported pregnancies must be followed to the completion/termination of the pregnancy. Pregnancy outcomes of spontaneous abortion, missed abortion, benign hydatidiform mole, blighted ovum, foetal death, intrauterine death, miscarriage and stillbirth must be reported as serious events (Important Medical Events). If the pregnancy continues to term, the outcome (health of infant) must also be reported using the Roche approved Pregnancy Outcome Report Form to the sponsor and be forwarded to Roche. Where the foetus may have been exposed to the Product, this information shall be transmitted to Roche by the Sponsor within thirty (30) calendar days of the awareness date.

16. STATISTICS

Definition of Outcome Measures

16.1 Primary Outcome

12 week Disease Control Rate (12wDCR): The number of participants with disease control at 12 weeks, defined using the modified mesothelioma RECIST 1.1 criteria (see Appendix A) as stable disease (SD), partial response (PR) and complete response (CR) as a proportion of evaluable participants.

Secondary Outcome

Objective response rate: The number of participants with a best response defined using the modified mesothelioma RECIST 1.1 criteria as partial response (PR) or complete response (CR) as a proportion of evaluable participants within 24 weeks.

Disease control rate at 24 weeks: The number of participants with disease control at 24 weeks following the end of cycle 6, defined using the modified mesothelioma RECIST 1.1 criteria as stable disease (SD), partial response (PR) and complete response (CR) as a proportion of evaluable participants.

Safety and Toxicity profile: Adverse events presented by seriousness, relatedness, expectedness and severity for each cycle until the end of the trial.

16.2 Hypothesis Tested

The null hypothesis states the true DCR is 0.25 and will be tested against a one-sided alternative hypothesis that the DCR will be greater than 0.25.

16.3 Sample Size

A total of 26 evaluable patients (n) will be analysed.

16.4 Description of Statistical Methods

16.4.1 Study design

The study design will comprise of a single-stage design. The type 1 error rate (one sided) is 0.05, and power 0.8. The 12 week disease control probability for poor drug activity p0 (null hypothesis) is 0.25 and for a good drug, P_1 of 0.5 (Hern, Statist Med, 2001, Table1).

If disease control is seen in 10 patients or less then no further investigation of the drug is warranted. Conversely, if this disease control is 11 or greater than the null hypothesis H_o will be rejected.

16.4.2 Descriptive analyses

Baseline characteristics of the patients will be summarised using means (standard deviations), or medians (interquartile range) for continuous variables and numbers (with percentages) for binary and categorical variables. Missing data will be tabulated.

The baseline variables that will be summarised includes the following:

- Age
- Gender
- Medical history
- Vital signs (including height, weight, temperature, blood pressure heart rate and respiratory frequency)
- Blood count
- Biochemistry profile (including: Urea, Electrolytes, calcium, phosphate, Liver Function Test, Thyroid function test, LDH & CRP)
- ECG

16.4.3 Primary analyses

The primary analysis of the primary outcome, 12wDCR, will be analysed in the efficacy population (detailed in section 16.4). Proportion of patients with DCR at 12 weeks will be presented along with exact 95% confidence intervals.

16.4.4 Secondary analyses

All secondary end-points will be analysed using the intention to treat (ITT) population, with the exception of adverse events, for which we will analyse the safety population, consisting of all participants who received at least one dose of trial medication (detailed in section 16.4). Proportion of patients with DCR and ORR at 24 weeks will be presented along with exact 95% confidence intervals.

Serious adverse events and adverse events will be detailed with the following summarised: cycle, number, event term, frequency, outcome, treatment given, severity (grade), action, related to IMP and expected. Safety and toxicity outcomes will be summarised using the safety population.

16.4.5 Exploratory analyses

All exploratory outcomes will be analysed using appropriate statistical tests according to the nature of the variables. No formal hypotheses testing will be performed.

16.5 Procedures for Reporting any Deviation(s) from the Original Statistical Plan

Any changes to the original statistical analysis plan will be detailed and justified in an updated statistical analysis plan.

16.6 Inclusion in Analysis

The efficacy population will comprise all patients who have received at least one dose of study drug. Any patients not achieving this, will be replaced in the study until all 26 patients have been enrolled.

The safety population will compromise of all individuals in the study who received at least one dose of the study drug.

The intention to treat population will compromise of all individuals that were entered in to the study (as detailed in section 16.2).

17. DIRECT ACCESS TO SOURCE DATA/DOCUMENTS

The participating NHS Trust as the site and the University of Leicester as the Sponsor will act to preserve patient confidentiality and will not disclose or reproduce any information by which participants could be identified, except where specific consent is obtained. This includes collection of NHS number, name and postcode to register and trace participants with the NHSIC. This also includes collection of NHS number or equivalent to utilise NHS data for future research.

Participant data will be entered at each participating site and retained in accordance with the Data Protection Act (2018). The PI is responsible for ensuring the accuracy, completeness, and timeliness of the data entered.

The participant data is pseudonymised by assigning each participant a participant identifier code which is used to identify the participant during the study and for any participant- specific clarification between the Mesothelioma Programme Team, LCTU and the participating site. The site retains a participant identification code list which is only available to site staff. The informed consent form will specify the participant data to be collected and how it will be managed or might be shared; including handling of all patient identifiable data (PID) and sensitive PID adhering to relevant data protection law.

Study documents will be retained in a secure location during and after the trial has finished.

The PI or delegate must maintain adequate and accurate records to enable the conduct of the trial to be fully documented and the trial data to be subsequently verified. After trial closure the PI will maintain all source documents and trial related documents. All source documents will be retained for a period of 25 years following the end of the trial. Sites are responsible for archiving the investigator site file and participants' medical records. The Sponsor is responsible for archiving the TMF and other relevant trial documentation.

Direct access will be granted to authorised representatives from the sponsor, host institution, drug provider and the regulatory authorities to permit trial-related monitoring, audits and inspections.

Data will be stored in a secure manner and will be registered in accordance with all relevant legislation e.g. General Data Protection Regulations (GDPR) 2018 and Data Protection Act 2018. The MiST Programme Team and the translational sample custodian for this trial is the Chief Investigator Professor Anne Thomas.

18. QUALITY CONTROL AND QUALITY ASSURANCE PROCEDURES

The study will be conducted in accordance with the current approved protocol, ICH GCP, relevant regulations and standard operating procedures.

Regular monitoring will be performed according to ICH GCP and a written monitoring plan. Data will be evaluated for compliance with the protocol and accuracy in relation to source documents. Following written standard operating procedures, the monitors will verify that the clinical trial is conducted and data are generated, documented and reported in compliance with the protocol, GCP and the applicable regulatory requirements.

19. CODES OF PRACTICE AND REGULATIONS

19.1 Ethics

This protocol will be submitted to a NHS Research Ethics Committee (REC) that is legally "recognised" by the United Kingdom Ethics Committee Authority for review and approval of clinical trials of investigational medicinal products. The approval of the REC must be obtained before the sponsor will authorise the start of a clinical trial or any trial procedures by way of issuing Sponsor Green Light.

19.2 Sponsor Standard Operating Procedures

All relevant Sponsor and LCTU SOPs will be followed to ensure that this study complies with all relevant legislation and guidelines.

19.3 Declaration of Helsinki

The Investigator will ensure that this study is conducted in full conformity with the current revision of the Declaration of Helsinki (last amended October 2000, with additional footnotes added 2002 and 2004).

19.4 ICH Guidelines for Good Clinical Practice

The Investigator will ensure that this study is conducted in full conformity with relevant regulations and with the ICH Guidelines for Good Clinical Practice (CPMP/ICH/135/95) July 1996.

19.5 Approvals

Once sponsor authorisation has been confirmed, the protocol, informed consent form, participant information sheet and any proposed advertising material will be submitted to an appropriate research ethics committee (REC), health research authority (HRA), regulatory authorities (MHRA in the UK), and host institution(s) for written approval.

The study will not commence until sponsor green light has been given.

Once Sponsor authorisation has been confirmed, the Investigator will submit and, where necessary, obtain approval from the above parties for all substantial amendments to the original approved documents.

19.6 Participant Confidentiality

The trial staff will ensure that the participants' anonymity is maintained. The participants will be identified only by initials and a participants ID number on the CRF and any electronic database. All documents will be stored securely and only accessible by trial staff and authorised personnel. The study will comply with the General Data Protection Regulations (GDPR) 2018 which requires data to be anonymised as soon as it is practical to do so.

20. SPONSORSHIP

The MiST study is being sponsored by University of Leicester. The University of Leicester shall be responsible for ensuring that the study is performed in accordance with all applicable regulatory guidelines.

20.1 Insurance & Indemnity

Insurance and/or indemnity to meet potential legal liability of the sponsor(s) and or employers(s) for harm to participants arising from the design and management of research, the University of Leicester insurance applies.

Insurance and/or indemnity to meet potential legal liability of investigators/ collaborators arising from harm to participants in the conduct of research, NHS indemnity applies.

21. UNIVERSITY OF LEICESTER DELEGATED RESPONSIBILITIES

21.1 University of Leicester has delegated the following responsibilities to the Chief Investigator:

The roles and responsibilities of the Chief investigator are documented in a signed and dated roles and responsibilities document.

21.2 Responsibilities Delegated to the University Hospitals of Leicester NHS Trust and the University of Leicester Laboratory

Storage and processing of blood and formalin fixed mesothelioma tissue.

22. DATA HANDLING AND RECORD KEEPING

All study data will be captured on paper or electronic CRFs and entered by staff at the site. Data will be captured on a validated MACRO database developed and maintained by the Leicester Clinical Trials Unit (LCTU).

The participants will be identified by a study specific participants number and/or code in any database. The name and any other identifying detail will NOT be included in any study data electronic file.

All data will be kept in locked filing cabinets at the research site whist patients undergo follow up and the final report is compiled. As soon as possible after publication of the final report all the documentation will be transferred to a designated GCP compliant offsite archive facility and kept for at least 25 years. Direct access will be granted to authorised representatives from the sponsor, host institution and the regulatory authorities to permit trial-related monitoring, audits and inspections. Data will be stored in a secure manner and will be registered in accordance with all relevant legislation e.g. General Data Protection Regulations (GDPR) 2018.

23. STUDY GOVERNANCE

23.1 Trial Management Group (TMG)

The TMG will comprise the Chief Investigator, and sub-investigators, clinical trial manager, trial statistician, lead research nurse, trial pharmacist, and research scientists/technicians and will convene every 3 months to assess the conduct of the study including recruitment, safety, and translational research.

23.2 Trial Steering Committee (TSC)

The TSC will comprise of the Chief Investigator and Co-Principal Investigators, with an Independent Chair and with at least one other independent clinician with expertise in a relevant field. The statistical team, and the trial management team may attend as required. This committee will be responsible for the overall management and oversight of the study and will normally meet within 1 month of the Data Safety Monitoring Committees (DSMCs) (although additional meetings may be called by the CI, TSC or DSMC chair) to review and approve protocol amendments and any sub-study proposals, review recruitment rates, protocol adherence, retention, compliance, safety issues, planned analyses and reports and act on recommendations of the DSMC. Minutes from the TSC will be copied to the Sponsor.

23.3 Data Safety Monitoring Committee (DSMC)

The DSMC will be appointed by the CI and the LCTU Statistical Team and will comprise members who are independent of the study, to include at least one statistician and at least one clinician with at least 3 members in total. The CI and/or trial manager will be invited to attend to provide specific input by the DSMC Chair. The meeting will consist of open and closed sections, with the CI and/or TM attending only the open section to discuss trial procedures and recruitment updates. The closed section will discuss unblinded data accrued to date and only the trial statistician will attend alongside the DSMC members.

The DSMC will review safety data regularly and make recommendations as to whether the study should continue, be modified or terminated. A log of all AEs will be provided to the DSMC for this purpose. The DSMC will review the statistical analysis plan prior to commencement of any analysis. The DSMC will first meet approximately 3 months from the first patients' first visit.

24. TRANSLATIONAL RESEARCH

Patients will be asked for consent for collection of tissue blocks before study entry. For patients giving consent for which no tissue is available, optional re-biopsy will be offered. In patients treated with atezolizumab and bevacizumab, at progression, optional consent for re-biopsy will be offered.

All patients' plasma samples will be collected before study entry and at disease progression.

The MiST4 atezolizumab and bevacizumab study tissue and blood collection will allow future studies to identify and validate predictive biomarkers and:

- Establish the correlation of loss of PDL1 expression (1-49% versus >50%) with response to atezolizumab and bevacizumab in patients with relapsed mesothelioma;

A test result will be available for PDL1 will be available on pre-screening and results will be available as a report to enable informing of the patient.

Archival Tissue collected at biopsy will be used to conduct tumour mutation burden analysis as a basis for identifying genomic correlates of exceptional response.

Formalin fixed paraffin embedded (FFPE); archival diagnostic mesothelioma tissue will be collected **on all patients entered onto the study**. Tumour mutation burden analysis will be conducted and correlated with response for all patients enrolled.

Patients whose tumours have responded to atezolizumab and bevacizumab (according to modified RECIST) will also be asked to donate an optional tissue sample upon progression in order to investigate correlates of acquired resistance. Consent for this will be sought at their first visit at study entry.

Analysis of the gut microbiome composition to correlate with response

25. FINANCE

The study is funded primarily by the British Lung Foundation (BLF).

Roche/Genentech, Inc. is providing free IMP (atezolizumab and bevacizumab) until disease progression, loss of clinical benefit, symptomatic deterioration, unacceptable toxicity, patient withdrawal or death, whichever comes first.

The Leicester Clinical Trials Unit has also been funded via the University of Leicester to support this trial. The trial is in the National Cancer Research Institute (NCRI) and National Institute for Health (NIHR) portfolio. Local CRN support should be available to support the entry of participants into this trial.

The HOPE Clinical Trials Facility based at the University Hospitals of Leicester NHS Trust has received funding via the University of Leicester to support the staff resource required to conduct this trial.

Support for research costs will be available to participating sites upon request.

26. PUBLICATION POLICY

Results of this study will be published as both an abstract and manuscript. The TMG will form the basis of the writing committee and advise on the nature of publications, subject to the Sponsor's requirements. Roche will need to see any proposed publication 30 days in advance of the publication release date.

All publications will include the list of named authors and will include the CI, Co-Investigators, Trial Manager, and Statistician(s) involved in the trial, as agreed by the CI. If there are no named authors then a writing committee will be identified.

27. REFERENCES

1. Burt, B.M., et al., Malignant pleural mesothelioma and the Society of Thoracic Surgeons Database: an analysis of surgical morbidity and mortality. J Thorac Cardiovasc Surg, 2014. 148(1): p. 30-5.

2. Fennell, D.A. and R.M. Rudd, Defective core-apoptosis signalling in diffuse malignant pleural mesothelioma: opportunities for effective drug development. Lancet Oncol, 2004. 5(6): p. 354-62.

3. Vogelzang, N.J., et al., Phase III study of pemetrexed in combination with cisplatin versus cisplatin alone in patients with malignant pleural mesothelioma. J Clin Oncol, 2003. 21(14): p. 2636-44.

4. Fennell, D.A., et al., Advances in the systemic therapy of malignant pleural mesothelioma. Nat Clin Pract Oncol, 2008. 5(3): p. 136-47.

5. Blayney, J.K., et al., Response to chemotherapy is predictive in relation to longer overall survival in an individual patient combined-analysis with pleural mesothelioma. Eur J Cancer, 2012. 48(16): p. 2983-92.

6. Lynch, T.J., et al., Activating mutations in the epidermal growth factor receptor underlying responsiveness of non-small-cell lung cancer to gefitinib. N Engl J Med, 2004. 350(21): p. 2129-39.

7. Mok, T.S., et al., Gefitinib or carboplatin-paclitaxel in pulmonary adenocarcinoma. N Engl J Med, 2009. 361(10): p. 947-57.

8. Maemondo, M., et al., Gefitinib or chemotherapy for non-small-cell lung cancer with mutated EGFR. N Engl J Med, 2010. 362(25): p. 2380-8.

9. Shaw, A.T., et al., Crizotinib versus chemotherapy in advanced ALK-positive lung cancer. N Engl J Med, 2013. 368(25): p. 2385-94.

10. Shaw, A.T., et al., Ceritinib in ALK-rearranged non-small-cell lung cancer. N Engl J Med, 2014. 370(13): p. 1189-97.

11. Motzer, R.J., et al., Nivolumab versus Everolimus in Advanced Renal-Cell Carcinoma. N Engl J Med, 2015. 373(19): p. 1803-13.

12. Robert, C., et al., Nivolumab in previously untreated melanoma without BRAF mutation. N Engl J Med, 2015. 372(4): p. 320-30.

13. Robert, C., et al., Pembrolizumab versus Ipilimumab in Advanced Melanoma. N Engl J Med, 2015. 372(26): p. 2521-32.

14. Larkin, J., et al., Combined Nivolumab and Ipilimumab or Monotherapy in Untreated Melanoma. N Engl J Med, 2015. 373(1): p. 23-34.

15. Garon, E.B., et al., Pembrolizumab for the treatment of non-small-cell lung cancer. N Engl J Med, 2015. 372(21): p. 2018-28.

16. Brahmer, J., et al., Nivolumab versus Docetaxel in Advanced Squamous-Cell Non-Small-Cell Lung Cancer. N Engl J Med, 2015. 373(2): p. 123-35.

17. Borghaei, H., et al., Nivolumab versus Docetaxel in Advanced Nonsquamous Non-Small-Cell Lung Cancer. N Engl J Med, 2015. 373(17): p. 1627-39.

18. Ansell, S.M., et al., PD-1 blockade with nivolumab in relapsed or refractory Hodgkin's lymphoma. N Engl J Med, 2015. 372(4): p. 311-9.

19. Wolchok, J.D., et al., Nivolumab plus ipilimumab in advanced melanoma. N Engl J Med, 2013. 369(2): p. 122-33.

20. Hamid, O., et al., Safety and tumor responses with lambrolizumab (anti-PD-1) in melanoma. N Engl J Med, 2013. 369(2): p. 134-44.

21. Nishimura, H., et al., Development of lupus-like autoimmune diseases by disruption of the PD-1 gene encoding an ITIM motif-carrying immunoreceptor. Immunity, 1999. 11(2): p. 141-51.

22. Freeman, G.J., et al., Engagement of the PD-1 immunoinhibitory receptor by a novel B7 family member leads to negative regulation of lymphocyte activation. J Exp Med, 2000. 192(7): p. 1027-34.

23. Iwai, Y., et al., Involvement of PD-L1 on tumor cells in the escape from host immune system and tumor immunotherapy by PD-L1 blockade. Proc Natl Acad Sci U S A, 2002. 99(19): p. 12293-7.

24. Dong, H., et al., Tumor-associated B7-H1 promotes T-cell apoptosis: a potential mechanism of immune evasion. Nat Med, 2002. 8(8): p. 793-800.

25. Topalian, S.L., et al., Safety, activity, and immune correlates of anti-PD-1 antibody in cancer. N Engl J Med, 2012. 366(26): p. 2443-54.

26. Ribas, A., Releasing the Brakes on Cancer Immunotherapy. N Engl J Med, 2015. 373(16): p. 1490-2.

27. Sznol, M. and D.L. Longo, Release the hounds! Activating the T-cell response to cancer. N Engl J Med, 2015. 372(4): p. 374-5.

28. Ivanov, S.V., et al., Genomic events associated with progression of pleural malignant mesothelioma. Int J Cancer, 2009. 124(3): p. 589-99.

29. Zalcman, G., et al., Bevacizumab for newly diagnosed pleural mesothelioma in the Mesothelioma Avastin Cisplatin Pemetrexed Study (MAPS): a randomised, controlled, open-label, phase 3 trial. Lancet, 2016. 387(10026): p. 1405-14.

30. Oxnard, G.R., et al., Response Rate as a Regulatory End Point in Single-Arm Studies of Advanced Solid Tumors. JAMA Oncol, 2016. 2(6): p. 772-9.

31. A'Hern, R. P. (2001). "Sample size tables for exact single-stage phase II designs." Stat Med 20(6): 859-866.

28. APPENDIX A:

Modified RECIST v1.1 Criteria to assess response to treatment

Tumour measurements for this study use modified mesothelioma RECIST 1.1 criteria (Byrne 2004, Eisenhauer EA 2009), which allow a combination of pleural and non-pleural disease to be measured and assessed.

Measurable disease is determined by at least one soft tissue malignant lesion with either 1) pleural or omental thickness of 10mm or more, or 2) long axis diameter of 10mm or more.

Target lesions

When present, pleural disease will be measured by taking uni-dimensional measurements of tumour thickness perpendicular to the chest wall or mediastinum. This will be at two positions at three different levels on CT scan. Transverse CT scan images should be used and levels chosen for measurements must be at least 1 cm apart. The measurement positions at each level should also be at least 1cm apart. The levels and positions used should be documented. The target lesions will be measured in such a way as to maximize the reproducibility of the measurement. At reassessment, pleural thickness must be measured at the same position and level. Only measurements 10mm or greater should be recorded at baseline. Where there is no lesion at position with a thickness 10mm or greater, the position should be recorded as “no measurement” and measurement recorded as “0”.

On each follow-up CT, target lesions will be assessed by making the measurements that correspond to those made at baseline. If there is tumour response, thickness at a position may measure less than 10mm, and these measurements should be recorded as accurately as possible. If a lesion is too small to be measured accurately, it can be recorded as 5mm. The sum of the (up to) six measurements defines the “pleural uni-dimensional measurement”.

Other disease sites, such as lymphadenopathy, contralateral pleural nodules, chest wall disease, abdominal disease, and metastases may be present. These should be recorded as per standard RECIST 1.1 guidelines, with a maximum of five measurements (maximum 2 of any type or in any organ system). Long-axis diameters should be recorded, except for lymph nodes (short axis diameter), and contralateral pleural disease or omental disease (maximum thickness). The sum of these (up to) five measurements defines the “non-pleural measurement”.

The “pleural uni-dimensional measurement” is added to the “non-pleural measurement” if present, to create the total tumour measurement.

Lesions that have received radiotherapy treatment are not allowed as target lesions unless they have shown subsequent progression.

Bone lesions are not allowed as target lesions unless they have a measurable soft tissue component.

<u>Target lesion response assessment:</u>
On subsequent scans, the combination of pleural and non-pleural target lesion measurements should be evaluated as:
Complete Response (CR): Disappearance of all target lesions,
Partial Response (PR): ≥30% decrease in SLD compared to the baseline measurements.
Progressive Disease (PD): ≥20% increase in SLD <u>AND</u> at least a 5mm overall increase, both compared to the minimum SLD (best response scan to-date)
Stable disease (SD): Between 30% decrease AND 20% increase in SLD (i.e. between (PR and PD)

<u>Non target lesions</u>
Tumour lesions that are not chosen for quantitative assessment will be documented and followed qualitatively as "non-target lesions", including other disease sites, lesions less than 10mm, and malignant lymph nodes between 10-15mm short-axis.

<u>Non-target lesion assessment</u>
On subsequent scans, "Non-target lesions" should be qualitatively evaluated as **Complete Response (CR):** Disappearance of all non-target lesions, **Progressive Disease (PD):** "Unequivocal"* progression of existing non-target lesions. The appearance of one or more "significant"* new lesions is also considered progression, **Non-CR/Non-PD:** Persistence of one or more non-target lesions(s).

Pleural effusions are considered non-evaluable for response assessment. The occurrence of pleural effusion or ascites can be considered as progressive disease if this is substantiated by positive cytology.

<u>Overall assessment</u>
- **Complete response (CR)**: the disappearance of all target and non-target lesions with no evidence of tumour elsewhere
- **Partial response (PR)**: A reduction of at least 30% in the sum of all measurements, with no evidence of significant disease progression in non-target lesions or new lesions. [In addition individuals are required to have no unequivocal progression of existing non-target lesions and no appearance of new lesions]
- Responses should be confirmed on the next trial CT, or a further CT scan at least 4 weeks after the first response.

- **Progressive disease (PD):** 1: an increase of at least 20% in the sum of all measurements compared with the previous best response (the smallest sum of

measurements on study to date), 2: the appearance of one or more significant* new lesions, 3: a significant* increase in target lesions

- **Stable disease (SD):** Neither sufficient shrinkage to qualify for Partial Response, nor sufficient increase to qualify for Progressive Disease, taking as reference both the baseline scan and best response to date.

* “Significant” or “unequivocal” are defined as new lesions greater than 5mm in size with progression that appears significant in light of the overall of disease (i.e. a qualitative assessment of an overall 20% increase in tumour diameters including new and non-target lesions.

29. APPENDIX B:

The New York Heart Association (NYHA) Functional Classification in a Patient with Heart Disease

Overview: The NYHA developed a functional classification for patients with heart disease.

Patients: Heart disease must be present.

Parameters:

- Limitations on physical activity
- Symptoms (undue fatigue palpitations dyspnea and/or anginal pain) with ordinary physical activity

Status at rest

Limitations on Physical Activity	Symptoms with Ordinary Physical Activity	Status at Rest	Class
none	none	comfortable	I
slight	symptomatic with ordinary activities	comfortable	II
marked	symptomatic at less than ordinary levels of activity	comfortable	III
unable to perform any activity	discomfort with any activity	symptomatic at rest	IV

From The Criteria Committee of the New York Heart Association. Nomenclature and Criteria for Diagnosis of Diseases of the Heart and Great Vessels. (1994). (9th ed.). Boston: Little, Brown & Co. 253 – 256.
