# Supplementary material for: A gut microbiota rheostat forecasts responsiveness to PD-L1 and VEGF blockade in mesothelioma
Source: Nat Commun. 2024 Aug 21;15:7187. doi: 10.1038/s41467-024-49842-5 (PMC11339264; doi:10.1038/s41467-024-49842-5)
Supplement: Supplementary file 3 — Reporting Summary [file 41467_2024_49842_MOESM3_ESM.pdf]

nature portfolio

Corresponding author(s): Professor Dean A. Fennell

Last updated by author(s): 24.02.2024

Reporting Summary

Nature Portfolio wishes to improve the reproducibility of the work that we publish. This form provides structure for consistency and transparency in reporting. For further information on Nature Portfolio policies, see our Editorial Policies and the Editorial Policy Checklist.

Statistics

For all statistical analyses, confirm that the following items are present in the figure legend, table legend, main text, or Methods section.

n/a	Confirmed	
☐	☒	The exact sample size (*n*) for each experimental group/condition, given as a discrete number and unit of measurement
☐	☒	A statement on whether measurements were taken from distinct samples or whether the same sample was measured repeatedly
☐	☒	The statistical test(s) used AND whether they are one- or two-sided<br>*Only common tests should be described solely by name; describe more complex techniques in the Methods section.*
☒	☐	A description of all covariates tested
☒	☐	A description of any assumptions or corrections, such as tests of normality and adjustment for multiple comparisons
☐	☒	A full description of the statistical parameters including central tendency (e.g. means) or other basic estimates (e.g. regression coefficient) AND variation (e.g. standard deviation) or associated estimates of uncertainty (e.g. confidence intervals)
☐	☒	For null hypothesis testing, the test statistic (e.g. *F*, *t*, *r*) with confidence intervals, effect sizes, degrees of freedom and *P* value noted<br>*Give P values as exact values whenever suitable.*
☒	☐	For Bayesian analysis, information on the choice of priors and Markov chain Monte Carlo settings
☒	☐	For hierarchical and complex designs, identification of the appropriate level for tests and full reporting of outcomes
☐	☒	Estimates of effect sizes (e.g. Cohen's *d*, Pearson's *r*), indicating how they were calculated

Our web collection on statistics for biologists contains articles on many of the points above.

Software and code

Policy information about availability of computer code

Data collection	*Provide a description of all commercial, open source and custom code used to collect the data in this study, specifying the version used OR state that no software was used.*
Data analysis	*Provide a description of all commercial, open source and custom code used to analyse the data in this study, specifying the version used OR state that no software was used.*

For manuscripts utilizing custom algorithms or software that are central to the research but not yet described in published literature, software must be made available to editors and reviewers. We strongly encourage code deposition in a community repository (e.g. GitHub). See the Nature Portfolio guidelines for submitting code & software for further information.

Data

Policy information about availability of data

All manuscripts must include a data availability statement. This statement should provide the following information, where applicable:

- Accession codes, unique identifiers, or web links for publicly available datasets
- A description of any restrictions on data availability
- For clinical datasets or third party data, please ensure that the statement adheres to our policy

Patient-related data related to this clinical trial shall remain confidential to the sponsor organisation (The University of Leicester) and will not be disclosed except where disclosure might be required in accordance with pharmacovigilance duties of the parties involved. Individual participant data can be made available, after

deidentification to investigators who provide written request in accordance with General Data Protection Regulation and following authorisation from the sponsor organisation, starting immediately and ending 3 years after publication. Requests for data and materials will be reviewed by the sponsor and any implications regarding intellectual property or confidentiality considered. The raw sequencing is available in SRA Run Selector via [https://www-ncbi-nlm-nih-gov.ezproxy3.lib.le.ac.uk/sra/?term=XXXXXXXXX] or [https://www-ncbi-nlm-nih-gov.ezproxy3.lib.le.ac.uk/sra/?term= XXXXXXXXX] which is hosted by the national Centre for Biotechnology Information, under accession number XXXXXXXXX. The data is searchable in Entrez via [https://www-ncbi-nlm-nih-gov.ezproxy3.lib.le.ac.uk/sra/?term= XXXXXXXXX]. All of the other data supporting the findings of this study are available within the article and its supplementary information files and from the corresponding author upon reasonable request.

Research involving human participants, their data, or biological material

Policy information about studies with human participants or human data. See also policy information about sex, gender (identity/presentation), and sexual orientation and race, ethnicity and racism.

Reporting on sex and gender	not applicable
Reporting on race, ethnicity, or other socially relevant groupings	not applicable
Population characteristics	Patients were eligible for the study if they were aged over 18, had evidence of radiologically progressing, histologically confirmed malignant mesothelioma after at least one course of systemic treatment for mesothelioma that included standard first-line pemetrexed and either cisplatin or carboplatin. Patients could be enrolled irrespective of the histological subtype and localisation of their primary mesothelioma—ie, pleural, peritoneal, or other. Any line of treatment was permitted (excluding any prior immunotherapy) with prior therapy completing no less than 14 days before treatment was initiated. Patients were required to have measurable disease by modified Response Evaluation Criteria in Solid Tumours for malignant mesothelioma (mRECIST1.1), predicted life expectancy of 12 weeks or more, Eastern Cooperative Oncology Group performance status score of 0–1, adequate haematological (full blood count including total white cell count, neutrophils, platelets and haemoglobin), renal (urea and electrolytes), and liver function tests (including bilirubin, alkaline phosphatase, alanine transaminase) and willingness to undertake research blood tests and optional tissue re-biopsy for translational research (please refer to the protocol in the supplementary materials).<br>Exclusion criteria included diagnosis or treatment of any other cancer within the 5 years before study entry, treatment with any agent with no marketing authorisation within 30 days before study entry, and palliative radiotherapy in the 4 weeks before baseline computerised tomography (CT) scan, uncontrolled brain metastases, and cardiac, respiratory, hepatic or renal insufficiency
Recruitment	patients with the above key inclusion criteria
Ethics oversight	Sponsor - University of Leicester

Note that full information on the approval of the study protocol must also be provided in the manuscript.

Field-specific reporting

Please select the one below that is the best fit for your research. If you are not sure, read the appropriate sections before making your selection.

☐ Life sciences ☐ Behavioural & social sciences ☐ Ecological, evolutionary & environmental sciences

For a reference copy of the document with all sections, see nature.com/documents/nr-reporting-summary-flat.pdf

Life sciences study design

All studies must disclose on these points even when the disclosure is negative.

Sample size	N=26<br><br>We used a single-stage A'Hern design with a type 1 error rate (one-sided) of 0·05, and power of 80%. The 12-week disease control rate parameters were set at p_0=0·25 (ie, a true disease control rate of 25% at 12 weeks would be too low, requiring no further evaluation therefore accepting the null hypothesis) and p_1=0·50 (ie, a true disease control rate of 50% at 12 weeks would be sufficient to warrant further evaluation). These parameters required a total of 26 evaluable patients to be analysed. On the basis of these assumptions, if 11 or more of the 26 enrolled patients achieved disease control at 12 weeks, we would conclude that the criteria for success had been met.
Data exclusions	not applicable
Replication	not applicable
Randomization	not applicable
Blinding	not applicable

Behavioural & social sciences study design

All studies must disclose on these points even when the disclosure is negative.

Study description	not applicable
Research sample	not applicable
Sampling strategy	not applicable
Data collection	not applicable
Timing	not applicable
Data exclusions	not applicable
Non-participation	not applicable
Randomization	not applicable

Ecological, evolutionary & environmental sciences study design

All studies must disclose on these points even when the disclosure is negative.

Study description	not applicable
Research sample	not applicable
Sampling strategy	not applicable
Data collection	not applicable
Timing and spatial scale	not applicable
Data exclusions	not applicable
Reproducibility	not applicable
Randomization	not applicable
Blinding	not applicable

Did the study involve field work? ☐ Yes ☒ No

Reporting for specific materials, systems and methods

We require information from authors about some types of materials, experimental systems and methods used in many studies. Here, indicate whether each material, system or method listed is relevant to your study. If you are not sure if a list item applies to your research, read the appropriate section before selecting a response.

Materials & experimental systems

n/a	Involved in the study	
☐	☒	Antibodies
☒	☐	Eukaryotic cell lines
☒	☐	Palaeontology and archaeology
☒	☐	Animals and other organisms
☐	☒	Clinical data
☒	☐	Dual use research of concern
☒	☐	Plants

Methods

n/a	Involved in the study	
☒	☐	ChIP-seq
☒	☐	Flow cytometry
☒	☐	MRI-based neuroimaging

Antibodies

Antibodies used

CD8 C8/144B 1:200 Dako Opal 480 (1:150) ; CD4 4B12 1:50 Dako Opal 520 (1:200) ; TIM-3 D5D5r 1:200 Cell Signalling Technology Opal 570 (1:250) ; TIGIT E5Y1W 1:50 Cell Signalling Technology Opal 620 (1:250) ; PD1 EH33 1:200 Cell Signalling Technology
Opal 690 (1:300)
CD19 EPR5906 1:300 Abcam Opal 780 (1:50)

Validation

Primary antibody list

CD4 4B12
target T-cell surface glycoprotein CD4 (Human); UniProt: P01730 - CD4_HUMAN
clone 4B12
host mouse
clonality monoclonal
conjugates unconjugated
specificity human
applications recommended by supplier IHC
manufacturer/supplier no available from Dako/Agilent, the same clone available from other suppliers
manufacturer website link concentrate discontinued, only version for Autostainer link 48 available
Antibodypedia no records
CiteAb https://www.citeab.com/antibodies/2390680-m7310-cd4-concentrate?des=e9cbc89d780af1ac
Human Protein Atlas no records
citations 142 citations listed in CiteAb records
additional info if available available in DAKO atlas of controls: https://www.agilent.com/cs/library/catalogs/public/00230-d58532-02-atlas-of-controls-2nd-edition-agilent.pdf

antibody used in paper for fluorescent multiplex immunohistochemistry in dilution 1:50 (data for discontinued concentrate version)

CD8 C8_144B
target T-cell surface glycoprotein CD8 alpha chain (Human); UniProt
P01732 - CD8A_HUMAN
clone C8/144B
host mouse
clonality monoclonal
conjugates unconjugated
specificity human
applications recommended by supplier IHC
manufacturer or supplier Dako/Agilent
manufacturer website link https://www.agilent.com/en/product/immunohistochemistry/antibodies-controls/primary-antibodies/cd8-(concentrate)-76631
Antibodypedia no records
CiteAb https://www.citeab.com/antibodies/2414791-m7103-cd8-concentrate?des=993ff4335ded26b6
Human Protein Atlas https://www.proteinatlas.org/ENSG00000153563-CD8A/summary/antibody
citations 634 citations listed in CiteAb records
additional info if available CE-IVD;

antibody used in paper for fluorescent multiplex immunohistochemistry in dilution 1:200

CD19 EPR5906
target B-lymphocyte antigen CD19 (Human); UniProt: P15391 - CD19_HUMAN
clone EPR5906
host rabbit
clonality recombinant monoclonal
conjugates unconjugated
specificity human
applications recommended by supplier flow cytometry, WB, IHC-P, ICC/IF, IHC-Fr
manufacturer or supplier Abcam
manufacturer website link https://www.abcam.com/products/primary-antibodies/cd19-antibody-epr5906-ab134114.html
Antibodypedia no records
CiteAb https://www.citeab.com/antibodies/719468-ab134114-anti-cd19-antibody-epr5906?des=550792dd45395dd9
Human Protein Atlas no records
citations 64 citations listed in CiteAb records
additional info if available

antibody used in paper for fluorescent multiplex immunohistochemistry in dilution 1:300

CD45RA 4KB5
target Receptor-type tyrosine-protein phosphatase C (Human); UniProt: P08575 - PTPRC_HUMAN

clone 4KB5
host mouse
clonality monoclonal
conjugates unconjugated
specificity human
applications recommended by supplier WB, IP, IF, IHC-P and flow cytometry
manufacturer or supplier Santa Cruz
manufacturer website link https://www.scbt.com/p/cd45ra-antibody-4kb5
Antibodypedia no records
CiteAb https://www.citeab.com/antibodies/809717-sc-20057-cd45ra-antibody-4kb5?des=a4a116238441a7a1
Human Protein Atlas no records
citations 2 citations listed in CiteAb records
additional info if available

antibody used in paper for fluorescent multiplex immunohistochemistry in dilution 1:1000

CD45RO UCHL1
Target Receptor-type tyrosine-protein phosphatase C (Human); UniProt: P08575 - PTPRC_HUMAN
Clone UCHL1
Host mouse
Clonality monoclonal
Conjugates unconjugated
Specificity human
applications recommended by supplier flow cytometry
manufacturer or supplier Dako/Agilent
manufacturer website link concentrate discontinued, only IVD version for flow cytometry available
Antibodypedia no records
CiteAb https://www.citeab.com/antibodies/2390694-m0742-cd45r0-concentrate
Human Protein Atlas https://www.proteinatlas.org/ENSG00000081237-PTPRC/summary/antibody
Citations 88 citations listed in CiteAb records
additional info if available

antibody used in paper for fluorescent multiplex immunohistochemistry in dilution 1:600

CD68 KP-1
Target
Macrosialin (Human); UniProt:
P34810 - CD68_HUMAN
Clone KP-1
Host mouse
Clonality monoclonal
Conjugates unconjugated
Specificity human
applications recommended by supplier IF, IHC, WB
manufacturer or supplier Dako/Agilent
manufacturer website link https://www.agilent.com/en/product/immunohistochemistry/antibodies-controls/primary-antibodies/cd68-(concentrate)-76535
Antibodypedia https://www.antibodypedia.com/explore/KP-1
CiteAb https://www.citeab.com/antibodies/2414857-m0814-cd68-concentrate?des=5ead40eb07739774
Human Protein Atlas no records
Citations 626 citations listed in CiteAb records
additional info if available

antibody used in paper for fluorescent multiplex immunohistochemistry in dilution 1:500

CD86 E2G8P
target T-lymphocyte activation antigen CD86 (Human): UniProt:
P42081 - CD86_HUMAN
clone E2G8P
host Rabbit
clonality monoclonal
conjugates unconjugated
specificity human, monkey
applications recommended by supplier IF, IHC, WB
manufacturer or supplier Cell Signalling Technology
manufacturer website link https://www.cellsignal.com/product/productDetail.jsp?productId=91882&utm_medium=b2b&utm_campaign=general
Antibodypedia https://www.antibodypedia.com/explore/E2G8P
CiteAb https://www.citeab.com/antibodies/6306439-91882-cd86-e2g8p-rabbit-mab?des=a1a0a6adecdada88
Human Protein Atlas no records
citations 34 citations listed in CiteAb records
additional info if available

antibody used in paper for fluorescent multiplex immunohistochemistry in dilution 1:75

CD103 EPR166(2)
target Integrin alpha-E (Human); UniProt:
P38570 - ITAE_HUMAN
clone EPR4166(2)
host Rabbit
clonality monoclonal
conjugates unconjugated
specificity human
applications recommended by supplier IF, IHC
manufacturer or supplier Abcam
manufacturer website link https://www.abcam.com/products/primary-antibodies/cd103-antibody-epr41662-ab129202.html
Antibodypedia no records
CiteAb https://www.citeab.com/antibodies/767425-ab129202-recombinant-anti-cd103-antibody-epr4166-2?des=f4ee304fe871ee79
Human Protein Atlas no records
citations 44 citations listed in CiteAb records
additional info if available

antibody used in paper for fluorescent multiplex immunohistochemistry in dilution 1:750

CD163 HPA046404
target Scavenger receptor cysteine-rich type 1 protein M130 (human); UniProt: Q86VB7
C163A_HUMAN
clone HPA046404
host rabbit
clonality polyclonal
conjugates unconjugated
specificity human
applications recommended by supplier IF, IHC
manufacturer or supplier Merck/Sigma
manufacturer website link https://www.sigmaaldrich.com/GB/en/search/hpa046404?focus=products&page=1&perpage=30&sort=relevance&term=HPA046404&type=product
Antibodypedia no records
CiteAb https://www.citeab.com/antibodies/10353902-hpa046404-anti-cd163-antibody-produced-in-rabbit?des=d9bde0e149986f56
Human Protein Atlas https://www.proteinatlas.org/ENSG00000177575-CD163/summary/antibody
citations 3 citations listed in CiteAb records
additional info if available

antibody used in paper for fluorescent multiplex immunohistochemistry in dilution 1:300

FOXP3 236_E7
target Forkhead box protein P3, Scurfin (human); UniProt: Q9BZS1
FOXP3_HUMAN
clone 236A/E7
host Mouse / IgG1, kappa
clonality monoclonal
conjugates unconjugated
specificity Human, Non-human primate, Rhesus monkey
applications recommended by supplier IHC, IP, Flow
manufacturer or supplier Invitrogen/ThermoFisher
manufacturer website link https://www.thermofisher.com/antibody/product/FOXP3-Antibody-clone-236A-E7-Monoclonal/14-4777-82
Antibodypedia https://www.antibodypedia.com/gene/485/FOXP3/antibody/3590841/14-4777-80
CiteAb https://www.citeab.com/antibodies/2041729-14-4777-foxp3-monoclonal-antibody-236a-e7-ebiosci?des=d05e92acbf0df1c1
Human Protein Atlas no records
citations 212 citations listed in CiteAb records
additional info if available

antibody used in paper for fluorescent multiplex immunohistochemistry in dilution 1:100

Granzyme B BLR22E
target Granzyme B (Human); UniProt: P10144 - GRAB_HUMAN
clone BLR022E
host Rabbit
clonality Recombinant Monoclonal
conjugates unconjugated
specificity human
applications recommended by supplier IHC, ICC, IF, WB
manufacturer or supplier Bethyl
manufacturer website link https://www.fortislife.com/products/primary-antibodies/rabbit-anti-granzyme-b-recombinant-monoclonal-antibody-blr022e/BETHYL-A700-022
Antibodypedia https://www.antibodypedia.com/gene/187/GZMB/antibody/5182771/A700-022
CiteAb https://www.citeab.com/antibodies/15803788-a700-022-rabbit-anti-granzyme-b-recombinant-monoclo?des=76ea8326b07c2c31

Human Protein Atlas no records
citations 0 citations listed in CiteAb records
additional info if available

antibody used in paper for fluorescent multiplex immunohistochemistry in dilution 1:500

Ki67 MIB-1
target Proliferation marker protein Ki-67 (Human); UniProt: P46013 - KI67_HUMAN
clone MIB-1
host Mouse
clonality monoclonal
conjugates unconjugated
specificity human
applications recommended by supplier IHC, IF
manufacturer or supplier Dako/Agilent
manufacturer website link https://www.agilent.com/en/product/immunohistochemistry/antibodies-controls/primary-antibodies/ki-67-antigen-(concentrate)-76646
Antibodypedia no records
CiteAb https://www.citeab.com/antibodies/2390690-m7240-ki-67-antigen-concentrate?des=c2b752b590fcb3ed
Human Protein Atlas https://www.proteinatlas.org/ENSG00000148773-MKI67
citations 2433 citations listed in CiteAb records
additional info if available

antibody used in paper for fluorescent multiplex immunohistochemistry in dilution 1:1000

PD1 EH33
target Programmed cell death protein 1 (Human); UniProt:
Q15116 - PDCD1_HUMAN
clone EH33
host Mouse
clonality monoclonal
conjugates unconjugated
specificity human
applications recommended by supplier IHC
manufacturer or supplier Cell Signalling Technology
manufacturer website link https://www.cellsignal.com/product/productDetail.jsp?productId=43248&utm_medium=b2b&utm_campaign=general
Antibodypedia https://www.antibodypedia.com/gene/20331/PDCD1/antibody/2871505/43248
CiteAb https://www.citeab.com/antibodies/3368996-43248-pd-1-eh33-mouse-mab?des=31d3d485b0544f88
Human Protein Atlas no records
citations 25 citations listed in CiteAb records
additional info if available

antibody used in paper for fluorescent multiplex immunohistochemistry in dilution 1:200

PDL1 E1L3N
target Programmed cell death 1 ligand 1 (Human); UniProt:
Q9NZQ7 - PD1L1_HUMAN
clone E1L3N
host Rabbit
clonality monoclonal
conjugates unconjugated
specificity human
applications recommended by supplier IHC, IP, Flow cytometry, WB
manufacturer or supplier Cell Signalling Technology
manufacturer website link https://www.cellsignal.com/product/productDetail.jsp?productId=13684&utm_medium=b2b&utm_campaign=general
Antibodypedia https://www.antibodypedia.com/gene/24139/CD274/antibody/1286946/13684
CiteAb https://www.citeab.com/antibodies/2043262-13684-pd-l1-e1l3n-xp-rabbit-mab?des=6d4a8ab1dea31948
Human Protein Atlas no records
citations 915 citations listed in CiteAb records
additional info if available

antibody used in paper for fluorescent multiplex immunohistochemistry in dilution 1:200

VISTA D1L2G
target V-type immunoglobulin domain-containing suppressor of T-cell activation (Human); UniProt:
Q9H7M9 - VISTA_HUMAN
clone D1L2G
host Rabbit
clonality monoclonal
conjugates unconjugated
specificity human
applications recommended by supplier IHC, WB, Flow
manufacturer or supplier Cell Signalling Technology
manufacturer website link https://www.cellsignal.com/product/productDetail.jsp?

productId=64953&utm_medium=b2b&utm_campaign=general
Antibodypedia https://www.antibodypedia.com/gene/2331/VSIR/antibody/2871564/64953
CiteAb https://www.citeab.com/antibodies/3368839-64953-vista-d1l2g-tm-xp-r-rabbit-mab?des=00d225121b26a75a
Human Protein Atlas no records
citations 14 citations listed in CiteAb records
additional info if available

antibody used in paper for fluorescent multiplex immunohistochemistry in dilution 1:200

Clinical data

Policy information about clinical studies
All manuscripts should comply with the ICMJE guidelines for publication of clinical research and a completed CONSORT checklist must be included with all submissions.

Clinical trial registration	NCT03654833
Study protocol	MIST4 protocol v1.0_14.06.2019 (protocol is provided in the supplementary materials
Data collection	January 2020 and June 2021, 30 patients were consented to participate
Outcomes	primary endpoint was 12 week progression free survival, secondary outcomes were 24 week progression free survival, objective response (mRECIST1.1), safety and tolerability

Plants

Seed stocks	not applicable
Novel plant genotypes	not applicable
Authentication	not applicable
